# Broad-spectrum CRISPR-Cas13a enables efficient phage genome editing

**DOI:** 10.1038/s41564-022-01258-x

**Published:** 2022-10-31

**Authors:** Benjamin A. Adler, Tomas Hessler, Brady F. Cress, Arushi Lahiri, Vivek K. Mutalik, Rodolphe Barrangou, Jillian Banfield, Jennifer A. Doudna

**Affiliations:** 1 California Institute for Quantitative Biosciences (QB3), University of California, Berkeley, CA USA; 2 Innovative Genomics Institute, University of California, Berkeley, CA USA; 3 Department of Earth and Planetary Science, University of California, Berkeley, CA USA; 4 Environmental Genomics and Systems Biology Division, Lawrence Berkeley National Laboratory, Berkeley, CA USA; 5 Department of Molecular and Cell Biology, University of California, Berkeley, CA USA; 6 Department of Food, Bioprocessing and Nutrition Sciences, North Carolina State University, Raleigh, NC USA; 7 Environmental Science, Policy and Management, University of California, Berkeley, CA USA; 8 University of Melbourne, Melbourne, Australia; 9 Howard Hughes Medical Institute, University of California, Berkeley, CA USA; 10 Department of Chemistry, University of California, Berkeley, CA USA; 11 MBIB Division, Lawrence Berkeley National Laboratory, Berkeley, CA USA

**Keywords:** Bacteriophages, Genetic engineering, Transcription, Microbiology techniques

## Abstract

CRISPR-Cas13 proteins are RNA-guided RNA nucleases that defend against incoming RNA and DNA phages by binding to complementary target phage transcripts followed by general, non-specific RNA degradation. Here we analysed the defensive capabilities of LbuCas13a from *Leptotrichia buccalis* and found it to have robust antiviral activity unaffected by target phage gene essentiality, gene expression timing or target sequence location. Furthermore, we find LbuCas13a antiviral activity to be broadly effective against a wide range of phages by challenging LbuCas13a against nine *E. coli* phages from diverse phylogenetic groups. Leveraging the versatility and potency enabled by LbuCas13a targeting, we applied LbuCas13a towards broad-spectrum phage editing. Using a two-step phage-editing and enrichment method, we achieved seven markerless genome edits in three diverse phages with 100% efficiency, including edits as large as multi-gene deletions and as small as replacing a single codon. Cas13a can be applied as a generalizable tool for editing the most abundant and diverse biological entities on Earth.

## Abstract (web summary)

A CRISPR-Cas13 enzyme is shown to have potent anti-phage activity that is harnessed to produce a phage genome engineering method with broad utility.

## Main

CRISPR-Cas systems confer diverse RNA-guided antiviral and anti-plasmid adaptive immunity in prokaryotes^1^. CRISPR genomic loci record phage infections over time in the form of sequence arrays comprising foreign DNA sequences (spacers) flanked by direct repeats. Array transcription and processing generate CRISPR RNAs (crRNAs) that associate with one or more cognate Cas proteins to form ribonucleoprotein complexes capable of recognizing crRNA-complementary DNA or RNA^2^. Upon target binding, Cas effectors disrupt phage infection using DNA cleavage^3–5^, RNA cleavage^6^, secondary messenger production^7,8^ or transcriptional silencing^9^. These programmable biochemical activities have been applied as genome editing tools in bacteria and eukaryotes^10^.

Due to the coevolutionary arms race between phages and their target bacteria, phages encode direct and indirect inhibitors of CRISPR-Cas systems^11–14^, employ DNA compartmentalizing or masking strategies^15–19^ and manipulate DNA-repair systems^20,21^. In addition, phages use population-level strategies to overwhelm^22,23^ and even destroy native CRISPR pathways^24^. This suite of active and passive DNA defence mechanisms has made it very difficult to generalize the use of any single DNA-targeting CRISPR effector as a sequence-guided phage genome-editing tool^25–28^.

Cas13 (formerly C2c2) effectors are RNA-guided RNA nucleases whose catalytic activity resides in two higher eukaryotic and prokaryotic nucleotide binding (HEPN) domains^6,29^. Distinct from other single-effector CRISPR-Cas systems, Cas13 can confer individual- and population-level defence against phage infection^30^. Upon target RNA binding, Cas13 unleashes general, non-specific RNA degradation that arrests growth of the virocell (infected cell^31^) to block infection progression, thereby limiting infection of neighbouring cells^30^. Four Cas13 subtypes (a–d) have been identified and differ by primary sequence and size as well as auxiliary gene association and extent of *cis*- versus *trans*-RNA cleavage activity^2^. Since all known viruses produce RNA^32^, Cas13 is capable of inhibiting double-stranded DNA (dsDNA) phages, primarily shown through studies investigating temperate^30,33,34^ and nucleus-forming^19,35^ phages. Class 2 CRISPR effectors tested so far have limitations and guide variability in overcoming the diversity of genetic content encoded in phages^12,19,20,27,36–38^. It remains unclear whether an RNA-targeting Cas13 can broadly protect bacteria from a range of dsDNA phages.

Here we characterized the ability of a single Cas13a variant to restrict wide-ranging phage infections in model bacterium *Escherichia coli*. Phage infection assays show that LbuCas13a is a robust inhibitor of phage infections across the *E. coli* phage phylogeny. Further, Cas13-mediated phage restriction is robust across a diversity of phage genome-protection strategies, lifestyles, genes and transcript features, enabling direct and specific phage interference. We demonstrate that Cas13’s potent broad-spectrum antiviral activity can be applied as a sequence-specific counterselection system suitable for recovering phage variants with edits as minimal as single codon replacement. Our results highlight the vulnerability of phage RNA molecules during phage infection and provide a robust generalizable strategy for phage genome engineering.

## Results

### Cas13 homologues are rare across bacterial phyla

**Fig. 1:**
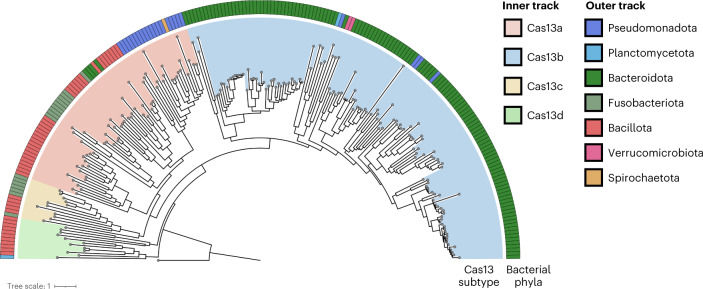
Maximum-likelihood phylogeny of Cas13 proteins and their distribution across the bacterial tree of life. The four known subtypes, Cas13a–d, each form their clade (inner track) with a skewed distribution across bacterial taxa (outer track). A *Vibrio cholerae* Cas9 (UIO88932.1) was used as the outgroup. Cas13 subtypes and microbial taxa that encode Cas13 are denoted in the colour bar. Source data

Phages encode diverse anti-defence strategies against the bacterial defence systems they are likely to encounter^13,33,39,40^, which in turn can render these systems ineffective for either phage immunity or phage engineering. To determine whether Cas13 might be useful as both a broad-spectrum phage defence and a phage genome editing tool, we began by investigating the distribution of Cas13 effectors across bacterial phyla. We performed a bioinformatic search for Cas13 proteins across NCBI and Genome Taxonomy Database (GTDB) genomes, culminating in a non-redundant set of 224 Cas13 protein sequences (Fig. 1 and Supplementary Fig. 1). Consistent with previous classification efforts^2^, Cas13 subtypes cluster into four clades 13a–d. We found Cas13b to be most widespread, yet predominantly found within Bacteroidota. In contrast, Cas13c and Cas13d subtypes appeared least common, primarily found in Fusobacteriota and Bacillota (formerly Firmicutes), respectively. We found Cas13a to be phylogenetically more widely dispersed, although relatively limited in total number of homologues, spread across Pseudomonadota (previously Proteobacteria), Bacillota, Bacteroidota and Fusobacteriota.

Our results are consistent with previous CRISPR search endeavours, suggesting that Cas13 effectors are some of the rarest Cas proteins currently identified^2^. Although RNA-targeting type-III CRISPR-Cas systems are relatively abundant in bacterial phyla^2^, we wondered whether the sparse occurrence of Cas13 effectors means that generalized resistance (for example, through RNA recycling^41^) or specialized resistance (for example, through anti-CRISPR^33^) to Cas13 is relatively rare as well.

### LbuCas13a is a potent anti-phage effector against phage T4

Two parsimonious explanations for the phylogenetic distribution of Cas13 effectors are that either Cas13 effectors are relatively ineffective anti-phage systems, limiting their phylogenetic spread owing to evolutionary pressure, or that Cas13 effectors are potent anti-phage systems, but the fitness cost of their abortive-infection-like effects^30,34^ causes selection against *cas13* loci. To explore these possibilities, we tested the anti-phage activity of the most- and least-widely dispersed Cas13 effectors on the basis of our analysis of bacterial phylogeny—Cas13a and Cas13d, respectively (Fig. 1). We selected LbuCas13a from *Leptotrichia buccalis* and RfxCas13d from *Ruminococcus flavefaciens* due to their extensive biochemical characterization^29,42–45^. We additionally selected an engineered variant of LbuCas13a (eLbuCas13a) that was recently reported to have lower basal *trans*-RNA cleavage activity and, thus, reduced toxicity when expressed in *E. coli*^45^. Notably, none of the Cas13 orthologues here have been investigated for anti-phage activity. While a Cas13a orthologue from *Listeria seeligeri* has been used to restrict temperate and nucleus-forming phages^19,30,33,35^, LbuCas13a comes from a phylogenetically distinct sub-clade of Cas13a effectors (Supplementary Fig. 1).

**Fig. 2:**
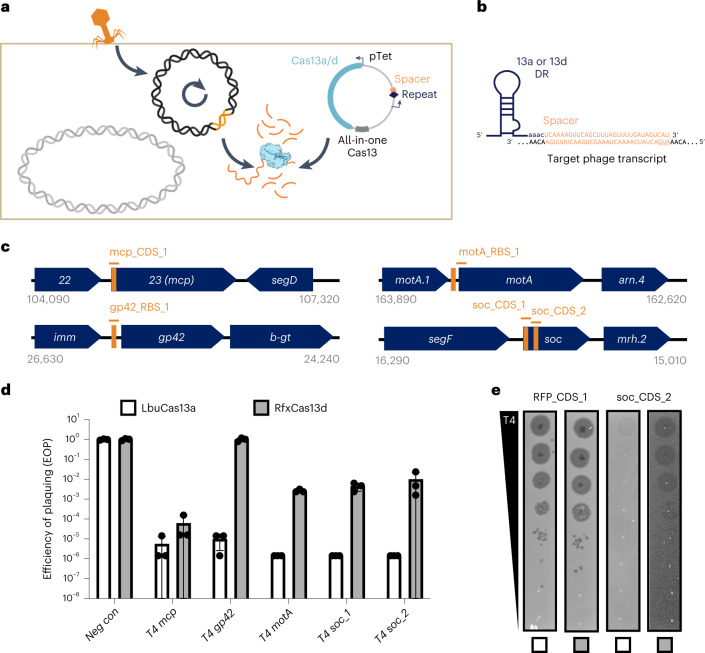
Comparison of Cas13a and Cas13d in *E. coli* phage challenge assays with lytic phage T4. **a**, Experimental architecture of Cas13 phage defence. Cas13 is expressed under aTc control alongside a crRNA. During phage infection, Cas13 unleashes toxic *cis*- and *trans*-cleavage if Cas13 detects its crRNA target. **b**, crRNA architecture employed in this study. **c**, Overview of T4 genes and transcript locations targeted by Cas13 in T4 phage challenge experiments. Approximate gene architecture is shown in forward orientation. crRNA locations are highlighted in orange. **d**, T4 phage infection in bacteria expressing phage-targeting crRNA and either LbuCas13a or RfxCas13d. EOP values represent the average of three biological replicates for a single crRNA. EOP data are presented as mean ± s.d. **e**, T4 phage plaque assays comparing the efficacy of Cas13a and toxicity of Cas13d. A representative plaque assay from three biological replicates is shown. An RFP-targeting crRNA is shown as a negative control. Source data

To establish an *E. coli* phage challenge assay for LbuCas13a and RfxCas13d, we created ‘all-in-one’ plasmids for inducible expression of *cas13* using anhydrotetracycline (aTc) alongside a constitutively expressed crRNA (direct repeat-spacer) (Fig. 2a,b). During phage infection, phage RNAs are transcribed, including a crRNA-targeted transcript (orange, Fig. 2a). Upon recognition, Cas13 activates HEPN-mediated RNA cleavage, although the extent of *trans*-cleavage may be reduced for Cas13d relative to Cas13a^43^. Depending on the extent of Cas13-mediated RNA cleavage, phage-encoded Cas13 resistance, protospacer mutation rate and phage-encoded function containing the protospacer, phage may overcome the resulting general transcript degradation.

To test the phage-restriction capacity of LbuCas13a and RfxCas13d outside their native context, we individually targeted a small panel of genes in phage T4. Phage T4 is a classical virulent dsDNA phage with a 169 kb genome and well-characterized genetic content^46,47^. From the perspective of phage genome editing, T4 represents an empirical challenge, displaying considerable variability in Cas-restriction efficacy for Cas9 and Cas12a, owing in part to modified glucosyl-5-hydroxymethylcytosine nucleotides^26,27,36^ and endogenous DNA-repair mechanisms^20^. For these reasons, we hypothesized that RNA targeting could be a superior strategy to inhibit T4 and related phages.

We designed a panel of Cas13 crRNAs targeting T4 transcripts with diverse design criteria (Fig. 2c)^46^. Targeted regions of T4^46^ RNA sequences included essential genes (major capsid protein (*mcp*), transcriptional activator *motA*), a conditionally essential gene (deoxycytidylate hydroxymethylase *gp42*), a non-essential gene (accessory capsid protein *soc*), an early-infection gene (*motA*), a middle-infection gene (*gp42*), late-infection genes (*mcp*, *soc*), encompassing regions early in coding sequences (CDSs) (*mcp*, *soc*), middle in CDS (*soc*) and untranslated regions around the ribosome binding site (RBS) (*gp42*, *motA*) (Supplementary Table 1 and Fig. 2). We included a red-fluorescent protein (RFP)-targeting crRNA as a negative control. Broadly, this panel of crRNAs represents a careful exploration of Cas13 targeting the diversity of feature types present in a phage transcriptome.

Remarkably, in phage infection experiments, we observed robust phage restriction for all crRNAs tested using LbuCas13a (Fig. 2d). Independent of gene essentiality, timing of expression or position on transcript, we found that crRNA-guided LbuCas13a could restrict phage T4 over 100,000× when targeting *mcp*, *gp42*, *motA* or *soc* (Supplementary Fig. 2). In contrast, crRNA-guided RfxCas13d exhibited highly variable and less-efficient phage restriction. Further, RfxCas13d exhibited phage-independent *E. coli* growth inhibition during RfxCas13d expression (Supplementary Figs. 2–4), and we also observed a high degree of phage escape for RfxCas13d relative to LbuCas13a (Fig. 2e and Supplementary Fig. 2). It is possible that RfxCas13d lacks crucial components required for full phage defence or reduced toxicity, such as the WYL domain-containing proteins that appear in its native gene neighbourhood (Supplementary Fig. 5). Our results suggest that LbuCas13a is a remarkably potent single-protein defence system of phage T4 relative to other CRISPR-Cas systems^20,26,27,36^.

### Cas13a confers resistance to diverse *E. coli* phages

**Fig. 3:**
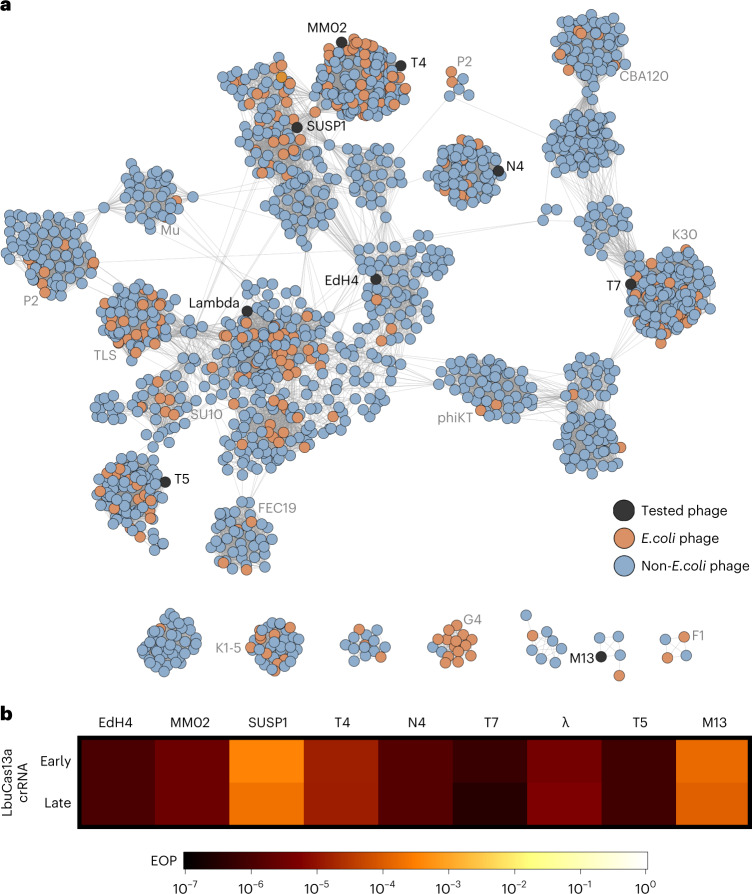
Comparison of LbuCas13a anti-phage activity across dsDNA *E. coli* phage phylogeny. **a**, Network graph representation of *E. coli* phages and their relatives. Nodes represent phage genomes that are connected by edges if they share significant similarity as determined by vContact2^76^ (protein similarity). Nodes are shaded red if they are classified as an *E. coli* phage and blue if they only share similarity. Nodes are shaded black if they were assessed for sensitivity to LbuCas13a. **b**, EOP experiments for Cas13a designed to target an early or late transcript. EOP values represent the average of three biological replicates for a single crRNA compared to an RFP-targeting negative control crRNA. Phages T4, EdH4, λ, T5 and T7 have additional crRNAs that were tested and are presented in Supplementary Figs. 2, 8, 10, 14 and 15, respectively. Source data

To the best of our knowledge, no single Cas effector (or antiviral defence protein) has been shown to confer broad-spectrum phage resistance against diverse dsDNA phages. To uncover the phage phylogenetic limits of Cas13a anti-phage activity, we challenged *E. coli* expressing LbuCas13a with a phylogenetically diverse panel of dsDNA *E. coli* phages. To generate a representative sampling of *E. coli* phages, we constructed a protein-sharing network from 2,307 phage genomes visualizing the relatedness of currently known *E. coli* phages (Fig. 3a). From this network, we assembled a panel of eight dsDNA and one single-stranded DNA (ssDNA) *E. coli* phages scattered across the *E. coli* phage phylogeny (Fig. 3a, Supplementary Figs. 6 and 7 and Table 2). This panel includes both model *E. coli* phages (T4, T5, T7, λ and M13) and non-model *E. coli* phages (EdH4, MM02, N4 and SUSP1). With the sole exception of phages T4 and MM02, these phages bear minimal nucleotide sequence similarity to each other (Fig. 3a and Supplementary Fig. 7). Furthermore, these phages have diverse lifestyles and reflect a realistic model sampling of the genetic diversity found among known *E. coli* phages. One of these phages displays temperate (λ), another displays chronic infection (M13^48^), while the remaining seven display obligately lytic life cycles. They comprise diverse lifestyles including documented plasmid-transfer-promoting (that is, ‘superspreader’)^49^, DNA compartmentalization^16^ and pseudolysogeny^50^ phenotypes. In aggregate, these phages not only represent genotypic diversity but also encompass a mixture of host-takeover strategies, modes of entry and degrees of previous characterization.

For each phage, we designed a pair of Cas13a crRNAs targeting either a putative early gene (DNA polymerase (*dnap*)), RNA polymerase (*rnap* (T7)), a lytic regulator (*cro*), replication protein (*II* (*rep*) (M13)) or a putative late gene (major capsid protein (*mcp* *VIII*) (M13)). An overview of Cas13-mediated phage restriction can be found in Supplementary Table 2, diversity of crRNAs tested in Supplementary Fig. 6 and a by-phage summary of results in Supplementary Figs. 2, 8–15. In aggregate, we observed substantial anti-phage activity for all 18 guides across the nine phages tested (Fig. 3b and Supplementary Table 2). Most crRNAs reduced phage infectivity 10^5^﻿–10^6^-fold, with the sparse observation of mature plaque-forming units (p.f.u.). Across this entire study, we observed no mature p.f.u.s above 0.01% frequency in wildtype (wt) phage lysates (Supplementary Figs. 2, 8–15). We observed a single guide (targeting T5 *dnap*) to yield general toxicity and growth inhibition during LbuCas13a induction (Supplementary Fig. 16). This constraint required us to perform assays in the absence of induction, achieving a mere 10^2^-fold restriction (Supplementary Fig. 14). However, employing the reduced-toxicity LbuCas13a mutant, eLbuCas13a^45^, we observed both phage restriction at 10^6^-fold (Supplementary Fig. 14) and slightly reduced toxicity in the absence of phage (Supplementary Fig. 16). Thus, we believe that the subpar phage restriction by LbuCas13a was attributed to elevated background toxicity of the T5*pol* spacer rather than an inability to target this phage gene.

Interestingly, SUSP1 and M13 consistently displayed a small degree of resistance to Cas13a (Fig. 3b). Both early- and late- transcript targeting guides only decreased phage infectivity 5,000–10,000-fold compared with all other phages showing 10^5^–10^6^-fold infectivity reduction. We further investigated the efficacy of SUSP1-targeting crRNAs in a plate-reader assay at a wide range of multiplicities of infection (MOIs) (Supplementary Fig. 17). Compared to a non-targeting crRNA control, we found that both SUSP1*dnap-* and SUSP1*mcp*-targeting guides conferred phage resistance at all MOIs tested, including MOIs >10. These results indicate that Cas13a targeting not only conferred substantial population-level protection against SUSP1 infection, but also single-cell protection^30^. Potentially, this discordance with the abortive-infection model of Cas13 protection observed previously^30^ reflects a feature of LbuCas13a, a feature of fitness in a non-native host for Cas13 or a feature of SUSP1 and should be investigated further. Overall, we find that LbuCas13a is capable of anti-phage activity with no identified limits across the tested coliphage phylogeny.

### A generalizable markerless method for editing phage genomes

The editing of virulent phage genomes has remained a major challenge for phage engineering and reverse genetics, largely due to the lack of universally applicable genetic tools or reliance on a native CRISPR-Cas system^25,26,28,37,51–54^. While the introduction of foreign gene content into phages is relatively straightforward to perform with homologous recombination (HR), ultimately the selection or screening for these rare recombinants is limiting even in well-characterized phages^53^. Given that LbuCas13a phage-restriction efficacy appears to have very little variability in terms of guide (Fig. 2), target (Figs. 2 and 3) and phage choice (Fig. 3), we suspected that Cas13a-mediated phage restriction would be an ideal tool for counterselection during phage genome editing. The high counterselection stringency observed earlier in this study obviates the need for selection markers, creating opportunities for multi-loci editing. Furthermore, the absence of protospacer-adjacent motif (PAM) requirements for LbuCas13a targeting^29^ suggests that virtually any position within or nearby a phage transcript could be edited and selected through LbuCas13a counterselection.

**Fig. 4:**
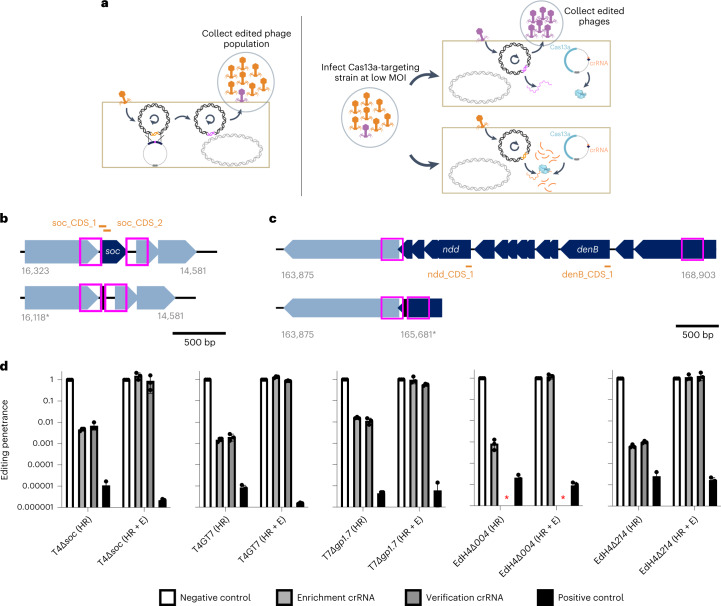
Cas13 facilitates a robust engineering strategy across diverse phages. **a**, Overview of a simple two-step editing process. Wildtype phage T4 infects homology vector-containing strain at a low MOI, yielding a mixed population of wt (orange) and edited (purple) phages (‘HR’). This population is diluted and infects a LbuCas13a-expressing strain targeting the wt locus, enriching for edited phages relative to wt (‘HR+E’). **b**, Example gene deletion design for T4∆*soc*. Top: gene organization of wt T4*soc* locus shown with approximate locations of *soc* protospacers (orange) and homology arms (pink box). Bottom: gene organization of edited T4∆*soc* locus. The encoded deletion removes both *soc* protospacers, enabling enrichment of edited phages. **c**, Example large multi-gene deletion design from T4*gp52.1* to T4*rIIB* (T4wtGT7). Top: gene organization of wt T4GT7 locus shown with approximate locations of T4*ndd* and T4*denB* protospacers (orange) and homology arms (pink box). Bottom: gene organization of edited T4GT7 locus. The encoded deletion removes both *soc* protospacers, enabling enrichment of edited phages. **d**, Editing penetrance (Methods) from three engineering replicates of the editing and enrichment process shown in **a** for T4*∆soc*, T4GT7, T7*∆gp1.7*, EdH4*∆gp004* and EdH4∆*gp214*. In all cases, ‘negative control crRNA’ refers to an RFP-targeting crRNA, ‘positive control crRNA’ refers to the corresponding phage’s *mcp*-targeting crRNA, ‘enrichment crRNA’ refers to the crRNA used during the enrichment step shown in **a** and ‘verification crRNA’ refers to the deletion-targeting crRNA not used during enrichment. The ‘verification crRNA’ for EdH4 yielded a very toxic phenotype to establish a titre and is denoted with a red asterisk. Editing penetrance data are presented as mean ± s.d. Source data

In principle, edits in the phage genome introduced through homologous recombination can escape LbuCas13a targeting, while wildtype phage cannot (Fig. 4a). To introduce and select for edits, we performed a simple two-stage homologous recombination and enrichment process (Fig. 4a, Supplementary Fig. 18 and Methods). Briefly, we employed two strains per edit: an editing strain containing a homologous recombination vector hosting a verification-primer binding site as well as 250 bp flanking phage homology arms, and a counterselection strain containing LbuCas13a and crRNA targeting the transcript carrying the locus to be edited. A pair of locus-specific examples are shown in Fig. 4b,c. We first infected the editing strain with wildtype phage at low MOI and collected the lysate consisting of a mixture of wildtype and edited phages (‘HR’ phage lysate) (Fig. 4a and Supplementary Fig. 18a). Then we diluted this lysate, infected the counterselection strain at low MOI and collected the resultant lysate (‘HR+E’ phage lysate) (Fig. 4a and Supplementary Fig. 18b).

As a proof of concept that such an editing approach is immediately applicable to reverse genetics in a diversity of phages, we designed a small panel of edits across phages T4, T7 and EdH4. In particular, we designed four single deletions (for example, Fig. 4b) encoding for edited phages T4∆*soc*, T7∆*gp1.7*, EdH4∆*gp004* and EdH4∆*gp214*. While T4*soc* and T7*gp1.7* (a nucleotide kinase) are known non-essential genes^46,55^ under standard laboratory conditions, phage EdH4 has neither been edited previously, nor is there any knowledge of its genes’ essentialities before this study. Thus, EdH4 represents a pressure test for how extensible this editing strategy is to other non-model phages. As an example of more complex edits, we also designed a large edit originally identified during forward genetic screens on T4 mutant T4GT7^36^ (hereafter, this edit in the wildtype T4 background is referred to as ‘T4wtGT7’). This edit consists of a large 3.2 kb deletion in T4, fully deleting 12 genes and truncating T4*gp52.1* and T4*rIIB* (Fig. 4c).

We designed two crRNAs disrupted by the edited phage locus of interest as well as an additional verification guide to confirm the entire gene deletion (examples for T4*soc* and T4wtGT7 are shown in Fig. 4b,c, respectively). When tested against wildtype phages T4, T7 and EdH4, candidate crRNAs for T4*soc*, T4*ndd* (nucleoid disruption protein), T4*denB* (endonuclease IV), T7*gp1.7*, EdH4*gp004* (hypothetical protein) and EdH4*gp214* (hypothetical protein) were approximately as effective in phage restriction as crRNAs targeting definitively essential genes such as *mcp* (Supplementary Figs. 2, 8 and 15). However, when targeting these putatively non-essential genes, we observed that plaques emerge at 10^−3^–10^−4^% frequency, potentially reflecting a low rate of mutative escape permitted by the genes’ non-essentiality. In line with the model of Cas13a primarily imparting phage defence through RNA *trans*-cleavage activity, these results indicate that the primary counterselection pressure does not depend on the essentiality of the crRNA target. One of these crRNAs, the verification crRNA for the EdH4*gp004* deletion, displayed elevated toxicity upon expression and is the only crRNA in this study we could not get to function. Ostensibly, LbuCas13a’s auto-toxicity is due to the extensive self-complementarity within the spacer of the mature crRNA (Supplementary Fig. 19) and potentially reveals a design constraint to be explored in future studies.

After each stage of editing (Fig. 4a), lysates were collected and titred against counterselection strains expressing LbuCas13a targeting the wildtype version of the edited locus (‘enrichment crRNA’ and ‘verification crRNA’), targeting an unedited locus (‘positive control crRNA’ (*mcp* crRNA)) and targeting a non-existent locus (‘negative control crRNA’ (RFP crRNA)) (Supplementary Figs. 20–24). By comparing the estimated titre against the non-targeting crRNA, we obtained a phenotypic estimate of the relative prevalence of edits within the population (that is, editing penetrance). Before enrichment (‘HR’), we observed targeted Cas13a-resistant infectious centres at 0.01–1% frequency for all five edits (Fig. 4d). Of particular note, edits for EdH4 and T4wtGT7 were generally lower in abundance, suggesting lower HR frequencies of editing for EdH4, as well as larger modifications. Importantly, after enrichment (‘HR+E’), targeted Cas13a-resistant infectious centre edited phages comprised nearly 100% of the population, while emergent general Cas13 resistance remained low (Fig. 4d and Table 1). In addition to confirming edits using the verification crRNA, we also PCR-verified nine plaques from the ‘HR+E’ lysates spotted on the counterselection crRNA for each edit (Supplementary Figs. 25–29). Unbiased PCR-derived Sanger sequencing further confirmed that the nature of Cas13 resistance was due to the designed edit in all cases.

**Table 1:** Summary of Cas13a-mediated phage genome editing

Edit name	Phage	Edited locus	Edit style and scope	Survivors detected?	Plaques screened	Mutant success rate (%)
T4*∆soc*	T4	*soc*	Deletion	Yes	9	100
T7*∆gp1.7*	T7	*gp1.7*	Deletion	Yes	9	100
*EdH4∆gp004*	EdH4	*gp004*	Deletion	Yes	9	100
*EdH4∆gp214*	EdH4	*gp214*	Deletion	Yes	9	100
T4wtGT7	T4	*gp52.1-rIIB*	Large deletion	Yes	9	100
*soc*-C	T4	*soc*	SNP (1)	No	N/A	N/A
*soc*-S	T4	*soc*	SNP (3)	No	N/A	N/A
*soc*-F	T4	*soc*	SNP (11)	Yes	9	100
*dnap*-C	T4	*dnap*	SNP (3)	Yes	9	100
*dnap*-S	T4	*dnap*	SNP (5)	Yes	9	100
*dnap*-F	T4	*dnap*	SNP (9)	Yes	9	100

Following specific editing experiments, no plaques were detected. Thus, screening plaques and calculating efficiency were not applicable (N/A).

### PAMless Cas13a enables minimal edits in phage genomes

**Fig. 5:**
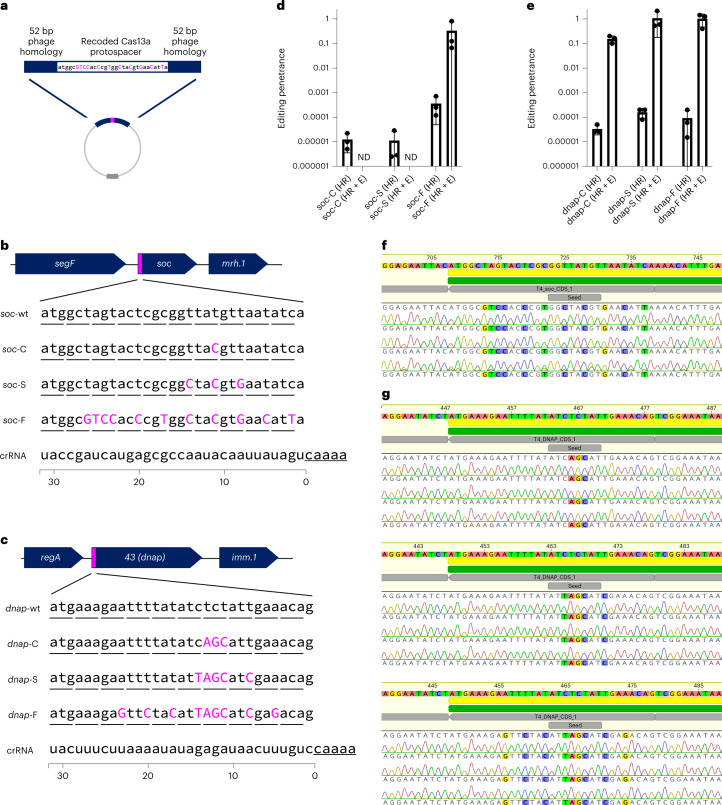
Minimal edits in phage T4 enabled by Cas13a counterselection. **a**, Homologous recombination vector design consists of a recoded Cas13a protospacer flanked by 52 bp of homology to the phage genome. **b**, Recoding design for a T4 non-essential gene, *soc*, with introduced silent mutations shown in magenta. Three designs with differing mutations were tested (*soc*-C, *soc*-S, *soc*-F). Underlined nucleotides represent the edge of the Cas13a CRISPR repeat. **c**, Recoding design for a T4 essential gene, *dnap*, with introduced silent mutations shown in magenta. Three designs with differing degrees of mutations were tested (*dnap*-C, *dnap*-S, *dnap*-F). Underlined nucleotides represent the edge of the Cas13a CRISPR repeat. **d**, Editing penetrance from three biological replicates of the editing and enrichment process shown in **b** for *soc*-C, *soc*-S and *soc*-F. Edited phage lysates with no detectable plaques are noted with ND. **e**, Editing penetrance from three biological replicates of the editing and enrichment process shown in **b** for *dnap*-C, *dnap*-S and *dnap*-F. Editing penetrance in **d** and **e** are presented as mean ± s.d. **f**, Unbiased sequencing of T4*soc* loci from individual plaques from three independent editing attempts. Deviations from wildtype are highlighted. **g**, Unbiased sequencing of T4dnap loci from individual plaques after editing attempts dnap-C (top), dnap-S (middle), and dnap-F (bottom), each with three independent editing attempts. Deviations from wildtype are highlighted. Sanger sequencing traces for all verified plaques including those shown in **f** and **g** can be found in Supplementary Figs. 16 and 20. Source data

We aimed to further take advantage of the flexibility enabled by Cas13a’s PAMless nature by creating and enriching minimal edits that only Cas13a could easily select for^20,26,27,36^, using T4 as a model virulent phage. We designed six mutants at either the non-essential *soc* gene or essential *dnap* using silent mutations, thus ‘recoding’ the target gene (Fig. 5). We designed these mutants to recode only a single codon (*soc*-C, *dnap*-C), recode the entire seed region (*soc-*S, *dnap*-S)^44^ or recode the full target (*soc-*F, *dnap*-F) (Fig. 5a–c). To facilitate homologous recombination-mediated edits, we flanked the intended mutation with 52 bp of native phage homology (Fig. 5a). Full phenotypic results from these recoding experiments for *soc* and *dnap* can be found in Supplementary Figs. 30–35.

For four of the six edits (*soc*-F, *dnap*-C, *dnap*-S, *dnap-*F), we observed that plaques emerge at 0.1–1% percent frequency in the ‘HR’ lysate (Supplementary Figs. 33–35). After enrichment on the Cas13a counterselection strain, targeted Cas13a-resistant infectious centres consisted of almost all of the phage population, suggesting high editing penetrance (Fig. 5d,e and Supplementary Figs. 33–35). In contrast, lysates containing *soc*-C and *soc*-S mutations went to extinction following enrichment, suggesting that the *soc*-C and *soc*-S mutations were insufficient to evade Cas13a activation (Fig. 5d and Supplementary Figs. 31 and 32). Comparing the design of *soc*-C, *soc-*S and *dnap*-C, multiple contiguous mutations within the seed region appear necessary to evade Cas13a activation during phage infection. Potentially, one of the reasons we observed very few escape mutants in wildtype phage lysates is that multiple contiguous mutations are necessary to evade Cas13a activation.

To verify that targeted Cas13a-resistant infectious centres were the result of intended edits, we performed unbiased PCRs at the wildtype locus for all editing attempts yielding plaques (Supplementary Figs. 33–35). In total, these consisted of 36 plaques across 4 unique edits (*soc-F*, *dnap-C*, *dnap-S*, *dnap-F*) and 12 independent editing processes. Strikingly, we found all 36 analysed plaques to have the intended mutation (Table 1 and Supplementary Figs. 36 and 37). It should be noted that for one engineering replicate of *soc-*F, we found two of the three plaques to encode a single nucleotide mutation (SNP) just outside of the site of the HR arm, probably reflecting an HR-induced source of error. Nonetheless, this editing process represents a simple straightforward route for enriching phage genome edits as small as one codon, as illustrated in the case of *dnap-C*.

## Discussion

We report that LbuCas13a transcript targeting is a broadly applicable, programmable phage counterselection pressure that can readily be converted into a phage genome editing tool. Despite belonging to one of the rarest CRISPR-Cas systems, we found LbuCas13a to be a potent RNA-guided anti-phage system. We challenged *E. coli* expressing Cas13a with nine diverse phages scattered across the *E. coli* phage phylogeny and found Cas13a to be effective at restricting all of them (>5,000-fold) (Fig. 3). While we anticipated that many phages would not harbour specific type-VI anti-CRISPR systems due to Cas13’s relative scarcity across bacterial phyla (Fig. 1), these results also suggest that it is rare to encode mechanisms to broadly recover from or prevent RNA degradation in phages. Furthermore, we observed very high crRNA efficacy and consistency between these phages and designed targets. Cas13a anti-phage activity was consistent and effective across gene essentiality, gene expression timing and target location within transcribed phage loci. In addition, LbuCas13a has no PAM requirements, has limited anti-tag inhibition^56^ and, as inferred from failed recoding attempts *soc*-C and *soc-*S, is tolerant to small mutations within its protospacer^44^ (Fig. 5d). Given these flexible target site constraints for LbuCas13a, we found the primary constraint on crRNA design to be spacer-specific auto-toxicity independent of the phage (Supplementary Figs. 3, 4 and 19). In *E. coli*, we found this toxicity readily circumventable by decreasing expression levels, using a reduced toxicity variant eLbuCas13a or by designing an alternative crRNA. Based on these observations, it appears that phages are generally vulnerable to Cas13a targeting.

Leveraging the broad vulnerability of phages to Cas13a, we demonstrated how this robust counterselection could be employed to enrich markerless genome edits in multiple *E. coli* phages. Most Cas-based counterselection methods show extensive crRNA or phage variability^25,28,35,52^, rely on native CRISPR host biology^51–54^ and/or yield a high rate of escape mutants^35,51^. Interestingly, during preparation of this manuscript, Guan et al. reported the use of LseCas13a and its cognate anti-CRISPR as a positive-selection strategy against nucleus-forming phages^35^. While they observed greater crRNA variability and a lower on-target editing penetrance, it is possible that a combination of use in a different host with different mutation rates and expression systems (*Pseudomonas aeruginosa*) and use of a different Cas13a orthologue (LseCas13a) is responsible for the crRNA variability. In contrast, we observed little variability in Cas13a counterselection efficacy across the 9 phages and 31 crRNAs tested in this study (with only one crRNA failing entirely; Supplementary Fig. 19). When applied to markerless genome editing, we measured a mutational penetrance of 100%—81/81 plaques across nine unique edits, three diverse phages and three independent editing attempts (Figs. 4 and 5 and Table 1). Through HR-mediated deletions or disruptions in the wildtype protospacer sequence, we proved that a wide diversity of phage genome edits are experimentally tractable, ranging from large deletions down to a single codon replacement. Due to the combination of flexibility and efficacy of phage targeting enabled by Cas13a, we anticipate that this phage selection strategy can enrich nearly any viable edit at transcribed loci in phages whose hosts can harbour and express LbuCas13a.

Possibly, the highly potent anti-phage activity observed in Cas13a is related to the relative scarcity of type-VI CRISPR-Cas systems. All known type-VI systems are thought to facilitate anti-phage activity through mechanisms similar to abortive infection^30,34^. Although the use of crRNA confers specificity for the activation of Cas13, in the absence of phage, we noticed toxicity upon LbuCas13a expression and substantial toxicity with RfxCas13d expression (Supplementary Figs. 3, 4 and 19). Potentially, the simultaneously increased toxicity and reduced phage restriction seen with RfxCas13d could be remedied by expression of additional proteins from its native locus, such as a pair of WYL domain-containing genes nearby (Supplementary Fig. 5), which has augmented orthologue RspCas13d in plasmid-restriction contexts previously described^57^. Additionally, we observed substantial auto-toxicity for LbuCas13a in conjunction with two crRNAs (targeting T5 *dnap* and EdH4*gp004*_2). The former was remedied by using a reduced *trans*-cleavage variant of LbuCas13a^45^ (Supplementary Figs. 14 and 16). However, we could not reduce the toxicity of the second crRNA targeting EdH4*gp004*, ostensibly due to intense secondary structure of the mature crRNA, raising a possible constraint on spacer design and natural selection (Supplementary Fig. 19). Nonetheless, from a phage restriction perspective, the high reliability of crRNA efficacy we observe in tandem with flexible crRNA design afforded by Cas13a means that these occasional limitations are easily circumventable. Perhaps the genetic stability and performance of this phage counterselection system would be more limited as it is applied in more diverse bacteria, phages with higher mutation rates and extends to weakly transcribed target sites.

In some respects, the seemingly universal efficacy of Cas13a against phages is surprising. RNA-cleaving HEPN domains, such as those in Cas13a^6,29^, are widely found across the tree of life, including *E. coli* and related bacteria^58–60^. Although phages encode inhibitors against HEPN domains^33,61^ and other endogenous RNAses, their ability to mitigate the toxic and anti-phage effects of Cas13a-mediated RNA *trans*-cleavage are relatively limited. Potentially, this reflects a conflicting role of RNA degradation as the infecting phage wrestles with the host for control of the transcriptome^41,62^. In contrast, phages encode a diversity of mechanisms to mitigate the effects of dsDNA cleavage, including nuclease inhibitors^11,13,14,63^, DNA modifications^15,17^, DNA-repair mechanisms^20,21^ and nucleic acid compartmentalization^16,18,19^. This comparative vulnerability to degenerate RNA cleavage we observe for phages at large highlights the centrality of RNA for viral infection^32^.

## Methods

### Bacterial strains and growth conditions

Cultures of *E. coli* were grown in lysogeny broth (LB Lennox) at 37 °C and 250 r.p.m. unless stated otherwise. When appropriate, 34 µg ml^−1^ chloramphenicol (+Ch) or 50 µg ml^−1^ kanamycin (+K) sulfate was supplemented to media. All bacterial strains were stored at −80 °C for long-term storage in 25% sterile glycerol (Sigma). Cloning and assays were primarily performed in DH10b genotype cells (NEB, Intact Genomics). For constructs targeting phage M13, cloning and assays were performed in DH5α F’I^q^ genotype cells (NEB).

### Phage propagation and scaling

Phages were propagated through commonly used protocols in LB media or LB top agar overlays (0.7%)^64^. Unless stated otherwise, phages were propagated on *E. coli* BW25113 (*lacI*^+^*rrnB*_T14_ Δ*lacZ*_WJ16_ *hsdR*514 Δ*araBAD*_AH33_ Δ*rhaBAD*_LD78_ *rph-1* Δ(*araB–D*)*567* Δ(*rhaD–B*)*568* Δ*lacZ4787*(::*rrnB-3*) *hsdR514 rph-1*). Phages N4, T4, T5 and T7 were scaled on *E. coli* BW25113^65^. Phage SUSP1 was a gift from Dr Sankar Adhya and scaled on *E. coli* BW25113^49^. Phages EdH4 and MM02 were obtained from DSMZ culture collection and scaled on *E. coli* BW25113 (DSM 103295 and DSM 29475, respectively)^66^. Phage λ cI857 *bor*::*kanR* was a gift from Dr Drew Endy and scaled as described previously^67^. All phages were titred through 2 µl spots of 10× serial dilution of phage in SM buffer (Teknova) on *E. coli* BW25113 in a 0.7% top agar overlay. Phage M13 was obtained from ATCC (15669-B1) and propagated on DH5α F’Iq genotype cells (NEB).

### Plasmid construction

A description of all plasmids, associated plasmid accessions and oligonucleotides to build them can be found in Supplementary Tables 3 and 4. Design of plasmids was performed manually, with visualization and sequence alignment performed in SnapGene. All plasmids used in this study were verified using whole-plasmid sequencing services offered by the UC Berkeley DNA Sequencing Facility. All plasmids were maintained as strains and maintained at −80 °C in 25% glycerol (Sigma).

All-in-one LbuCas13a, eLbuCas13a and RfxCas13d plasmids were designed to include a Cas13 effector under tetR-pTet control and a crRNA placeholder under constitutive expression on a p15a-CmR backbone. The crRNA placeholder sequence consisted of a constitutive promoter followed by the corresponding CRISPR direct repeat, a BsaI dropout site (aaacA**GAGACC**TCGTTTACCTATC**GGTCTC**atgct; BsaI sites shown in bold, flanking regions in lower case, and BsaI overhangs underlined), and a terminator. LbuCas13a, eLbuCas13a and RfxCas13d entry vectors were constructed through Gibson assembly (NEB, E2611L)^68^, yielding plasmids pBA559, pBA560 and pBA562, respectively. Assembly of pBA559, pBA560 and pBA562 used PCRs derived from pEJC 1.2 Lbu, pEJC 1.2 Lbu A12 and pEJC 1.5 CasRX vectors that were gifts from Drs Emeric Charles and David Savage^45^. Gibson reactions were purified with DNA Clean & Concentrator-5 (Zymo Research) and electroporated into DH10b (NEB, Intact Genomics).

crRNA spacers were introduced to pBA559, pBA560 and pBA562 through BsaIHFv2 (NEB, R3733L) golden-gate assembly^69^. Spacers were ordered as two complementary oligonucleotides with 4 bp 5′ overhangs matching the BsaI-digested destination plasmid staggered ends, phosphorylated with T4 PNK (NEB) at 37 °C for 30 min and duplexed (10 uM) by melting at 100 °C for 5 min, followed by slow cooling to room temperature over 15 min. PNK-annealed spacer duplex (100 fmol) were used as insert template in each golden-gate reaction. Golden-gate reactions for crRNA assembly were purified with DNA Clean & Concentrator-5 (Zymo Research), electroporated into DH10b (NEB, Intact Genomics) and plated on LB+Ch at 37 °C.

HR donor vectors were assembled through BbsI (NEB, R0539L) golden-gate assembly^69^. For HR vectors, pBA707 was used as an entry vector. pBA707 contains a BBR1-KanR backbone with an RFP dropout cassette. Briefly, the RFP dropout cassette consists of an RFP expression cassette flanked by BbsI restriction sites revealing 3′-ATAG-5′ and 5′-AGGA-3′ overhangs. Upon successful digestion and ligation with an appropriate insert containing 5′-TATC-3′ and 3′-TCCT-5′ overhangs, both the RFP expression cassette and BbsI sites are lost, revealing RFP colonies.

For gene deletion vector designs, gene fragments (ordered from TWIST Biosciences) consisting of BbsI cut sites compatible with pBA707 flanking sequences encoding for the corresponding gene deletion (gbBA086, gbBA089, gbBA102 and gbBA103) (Supplementary Table 5; for design details, see ‘Homologous recombination donor vector design’). For recoding vector designs, 5′-phosphorylated (with T4 PNK; NEB) and annealed oligonucleotides were used for UP-homology (oBA1761/oBA1762 or oBA1765/oBA1766), DN-homology (oBA1763/oBA1764 or oBA1767/oBA1768) and mutated protospacer (oBA1769/oBA1770, oBA1771/oBA1772, oBA1773/oBA1774, oBA1775/oBA1776, oBA1777/oBA1778 or oBA1779/oBA1780). Golden-gate reactions were purified with DNA Clean & Concentrator-5 (Zymo Research), electroporated into DH10b (NEB, Intact Genomics) and plated on LB+K at 37 °C. In all cases, RFP-negative colonies were chosen for sequence verification.

### crRNA design

A complete summary of the spacers used in this study can be found in Supplementary Table 1. We manually designed all Cas13a/d crRNAs as 31 nt spacers complementary to target phage transcripts (that is, a 31 nucleotide spacer identical to the reverse-complement of a phage gene). Spacers were chosen with no substantial bias against or towards any protospacer flanking sequence and minimal additional heuristics. Spacers were exclusively chosen to target predicted phage transcripts or a non-targeting control on the basis of published genome sequences for phage λ (J02459.1), EdH4 (MK327930.1), M13 (NC_003287.2), MM02 (MK373784.1), N4 (NC_008720.1), SUSP1 (NC_028808.2), T4 (NC_000866.4), T5 (NC_005859.1) and T7 (NC_001604.1). Because DH10b harbours λ-like prophage, φ80lacZΔM15, spacers were designed to avoid similarity to the DH10b genome (NC_010473.1)^70^.

When targeting the CDS of a phage gene, the transcript corresponding to the first 31 nucleotides of a CDS was targeted by default. For verification of gene deletions, an additional guide targeting 0–15 nucleotides downstream of the transcript was chosen. When targeting ‘RBS’ sequences of T4*gp42* and T4*motA*, spacers were designed to target transcripts beginning with the -4 to -6 positions of the gene. For all negative controls, an RFP-targeting spacer (‘AACTCTTTGATAACGTCTTCGCTACTCGCCA’) was used as it represented a functional spacer targeting an RNA transcript absent within our experiments.

### Homologous recombination donor vector design

Gene deletions in phages T4, T7 and EdH4 HR vectors were designed with 250 bp phage homology (‘UP’), a common primer binding region for verification and 250 bp phage homology (‘DN’). For T4*soc*, T7*gp1.7* and EdH4*gp0214* gene deletions, phage homology was chosen leaving both the native start and stop codons intact. For re-creation of the T4 ‘GT7-like’ large deletion^36^, homology was inferred from alignment of T4GT7 (KJ477686.1) to wildtype T4 (NC000866.4). This design yielded a 3,254 bp deletion of T4 from positions 165,257–168,510, entirely removing 12 genes and partially removing T4*52.1* and T4*rIIB*.

For minimal recoding edits, HR donor vectors were designed with 52 nt of homology upstream (UP) and downstream (DN) of a targeted protospacer on the phage genome. To encode minimal edits, predicted codons were converted to silent mutations in a single codon (-C), seed region (-S) or full protospacer (-F) using a coding table for *E. coli*. When possible, codons were maximally altered and rare codons avoided to minimize non-Cas13 phenotypic consequence. The seed region was estimated as previously observed in vitro^44^.

### Efficiency of plaquing assays

Bacteriophage assays were conducted using a modified double agar overlay protocol. For each Cas13-crRNA-phage combination, a strain of DH10b (NEB, Intact Genomics) containing a Cas13-crRNA plasmid (Supplementary Table 3) was grown overnight at 37 °C and 250 r.p.m. To perform plaque assays, 100 µl of saturated overnight culture was mixed with molten LB Lennox top agar supplemented with appropriate inducer and antibiotics and decanted onto a corresponding LB Lennox agar plate (to final overlay concentrations of 0.7% (w/v) agar, 5 nM aTc and 34 µg ml^−1^ chloramphenicol). For all phage experiments in this study, no supplementary CaCl_2_ or MgSO_4_ salts were added. For pBA675 and pBFC1053, toxicity was apparent at 5 nM aTc, so lower levels of aTc were used (0 and 1 nM aTc, respectively). For pBA769, assays were performed at 10 nM aTc to achieve restriction against phage SUSP1. Overlays were left to dry for 15 min under microbiological flame. For each Cas13-crRNA-phage combination, 10X serial dilutions of the appropriate phage were performed in SM buffer (Teknova), and 2 µl of each dilution were spotted onto the top agar and allowed to dry for 10 min. Plaque assays were incubated at 37 °C for 12–16 hours. After overnight incubation, plaques were scanned using a standard photo scanner and plaque-forming units (p.f.u.s) enumerated. In cases where individual p.f.u.s were not enumerable but clearings were observed at high phage concentrations, we interpreted these cases as ‘lysis from without’ and indicated a lack of productive phage infection^71^. As an estimate of an upper bound of phage infection for these cases, the most concentrated dilution at which no individual plaques were observed was approximated as 1 p.f.u. Efficiency of plaquing (EOP) calculations for a given condition were performed by normalizing the mean p.f.u. for a condition to the mean p.f.u. of a non-targeting control: mean(p.f.u._condition_)/mean(p.f.u._negativecontrol_). All plaque assays were performed in biological triplicate. Calculations were performed using GraphPad Prism.

### Liquid growth curve assays

Liquid phage experiments were performed in a Biotek plate reader at determined levels of aTc induction. Briefly, for each Cas13-crRNA combination, a strain of DH10b (NEB, Intact Genomics) containing a Cas13-crRNA plasmid (Supplementary Table 3) was grown overnight at 37 °C and 250 r.p.m. Strains were seeded in wells at 8 × 10^6^ colony-forming units (c.f.u.), and 200 µl of LB+Ch media containing 0, 1 nM, 10 nM or 100 nM aTc was added to each well. Infection was monitored in a Biotek Cytation 5 plate reader for 16 hours, with 200 r.p.m. shaking at 37 °C, with optical density (OD)_600_ readings every 5 min. All growth assays were performed in biological triplicate beginning from three independent overnight bacterial cultures. Data were plotted using the seaborn package in Python.

### Liquid phage infection assays

Liquid phage experiments were performed in a Biotek plate reader at determined MOIs. Briefly, for each Cas13-crRNA-phage combination, a strain of DH10b (NEB, Intact Genomics) containing a Cas13-crRNA plasmid (Supplementary Table 3) was grown overnight at 37 °C and 250 r.p.m. Strains were seeded in fresh media (LB+Ch+10 nM aTc) to an OD_600_ of 0.04 and 200 µl transferred to a 96-well plate (Corning 3904), achieving a final cell count of ~8 × 10^6^ c.f.u. per well. Appropriate phages were diluted in SM buffer (Teknova) to a maximal titre of 10^11^ p.f.u. per ml and 10X serially diluted 7 times. To begin phage infection, 1 µl of phage was added to achieve MOIs of 1.25*10-6 to 12.5. Infection was monitored in a Biotek Cytation 5 plate reader for 16 hours, with 200 r.p.m. shaking at 37 °C, with OD_600_ readings every 5 min. All infection assays were performed in biological triplicate beginning from three independent overnight bacterial cultures. Data were plotted using the seaborn package in Python.

### Phage genome editing experiments

A graphical overview of the phage genome editing experiments is shown in Supplementary Fig. 18. All assays were performed in biological triplicate beginning from three independent overnight bacterial cultures. All editing workflows occurred in parallel processes (that is, ‘editing’ replicates).

To create genome-edited phage lysates, a phage-editing strain consisting of DH10b (NEB, Intact Genomics) containing a homologous recombination vector (pBA1015 (T7∆*gp1.7*), pBA1018 (T4wtGT7), pBA1030 (EdH4∆*gp004*), pBA1031 (EdH4∆*gp214*), pBA1032 (T4∆*soc*) or pBA787-pBA792 (recoding experiments)) (Supplementary Table 3) was grown overnight in LB+K media at 37 °C and 250 r.p.m. Strains were diluted into fresh media (LB+K) to an OD_600_ of 0.04 and 200 µl transferred to a 96-well plate (Corning 3904), achieving a final cell count of ~8 × 10^6^ c.f.u. per well. Wildtype phage was added to each well to achieve an MOI of 0.01 (~8 × 10^4^ p.f.u. of phage). Infection was monitored in a Biotek Cytation 5 plate reader at 200 r.p.m. shaking at 37 °C, with OD_600_ readings every 5 min. Infection was allowed to proceed until there was a visible population crash (~4.5–7 hours depending on the phage). Lysates were transferred to a 96-well block (Greiner 780271-FD), and one drop of chloroform (Sigma) was added to lyse remaining bacteria. These lysates comprise a mixture of homologous recombination-edited phage and wildtype phage and comprised the ‘HR’ phage lysate. Blocks were covered with an aluminum seal (Corning 6570). ‘HR’ phage lysates were stored at 4 °C until use and titred before enrichment.

To enrich genome-edited phage lysates, a phage counterselection strain consisting of DH10b (NEB, Intact Genomics) containing an ‘enrichment’ Cas13a vector (pBA1034 for T7∆*gp1.7*, pBA1038 for T4wtGT7, pBA1042 for EdH4∆*gp004*, pBA1044 for EdH4∆*gp214*, pBA691 for T4∆*soc* and T4*soc* recoding, or pBA778 for T4*dnap* recoding) (Supplementary Table 3) was grown overnight in LB+Ch media at 37 °C and 250 r.p.m. Strains were diluted into fresh media (LB+Ch+10 nM aTc) to an OD_600_ of 0.04 and 200 µl transferred to a 96-well plate (Corning 3904), achieving a final cell count of ~8 × 10^6^ c.f.u. per well. ‘HR phage lysate’ was added to each well to achieve an MOI of 0.01 (~8 × 10^4^ p.f.u. of total phage titre). Infection was monitored in a Biotek Cytation 5 plate reader at 200 r.p.m. shaking at 37 °C, with OD_600_ readings every 5 min. Infection was allowed to proceed until there was a visible population crash (~7 hours). Lysates were transferred to a 96-well block (Greiner 780271-FD), and one drop of chloroform (Sigma) was added to lyse remaining bacteria. These lysates comprise an enriched mixture of homologous recombination-edited phage and wildtype phage and comprised the ‘HR+E’ phage lysate. Blocks were covered with an aluminum seal (Corning 6570). ‘HR+E’ phage lysates were stored at 4 °C until use.

### Determination of phage genome editing penetrance

Phage-editing penetrance was determined by plaque assay of ‘HR’ and ‘HR+E’ lysates on non-selective and wt-phage-counterselective strains. For T7∆*gp1.7* experiments, pBA1034 was used as the ‘enrichment crRNA’, pBA1035 as the ‘verification crRNA’, pBA678 as the ‘positive control crRNA’ and pBA620 as the ‘negative control crRNA’. For EdH4∆*gp004* experiments, pBA1042 was used as the ‘enrichment crRNA’, pBA1043 as the ‘verification crRNA’, pBA823 as the ‘positive control crRNA’ and pBA620 as the ‘negative control crRNA’. For EdH4∆*gp214* experiments, pBA1044 was used as the ‘enrichment crRNA’, pBA1045 as the ‘verification crRNA’, pBA823 as the ‘positive control crRNA’ and pBA620 as the ‘negative control crRNA’. For T4∆*soc* experiments, pBA673 was used as the ‘enrichment crRNA’, pBA674 as the ‘verification crRNA’, pBA647 as the ‘positive control crRNA’ and pBA620 as the ‘negative control crRNA’. For T4wtGT7 experiments, pBA1038 was used as the ‘enrichment crRNA’, pBA1039 as the ‘verification crRNA’, pBA647 as the ‘positive control crRNA’ and pBA620 as the ‘negative control crRNA’. For *soc* recoding edits, 10 nM aTc induction was used for strains containing pBA620 as a negative control and pBA691 as an ‘enrichment crRNA’ Cas13 vector. For *dnap* edits, 5 nM aTc induction was used for strains containing pBA620 as a negative control and pBA778 as an ‘enrichment crRNA’ Cas13 vector. For all edited phages, penetrance was defined as p.f.u._enrichment_/p.f.u._negative_. Average penetrance was calculated across independent editing attempts. Penetrance calculations were performed in Graphpad Prism.

To confirm the genotype of edits, we performed unbiased PCRs followed by Sanger sequencing. In addition, for gene deletions we performed N-terminal and C-terminal PCRs. Primers for unbiased PCRs were designed to amplify from the phage genome 150–300 bp outside of the UP and DN homology arms supplied from the editing vectors. For N-terminal and C-terminal PCRs, reverse (oBA2074:) and forward (oBA2075) facing primers touchdown on the small primer binding site provided on HR vectors (GATAAGAGACGGCTCAACGCCCGTCTCACAGC). PCRs were performed on three individual plaques from each ‘HR+E’ lysate after plaquing on the ‘enrichment crRNA’ strain. Plaques were picked into 50 µl SM buffer (Teknova) and allowed to diffuse out of the plaque plug at 4 °C overnight. To prepare for PCR and denature phage virions, 10 µl of these samples were transferred to PCR tubes and boiled at 100 °C for 10 min. PCRs were visualized using 1% agarose gels stained with SYBR-SAFE and imaged using a BioRad gel imager using auto-exposure settings. Sanger sequencing traces were visualized using Geneious.

### Cas13 phylogenetic tree

Cas13-annotated protein sequences were compiled from NCBI and were identified in GTDB r95 using custom cas13 Hidden Markov Models. All sequences that did not contain two R/Q/N/K/H/****H sequence motifs were removed. CD-HIT v4.8.1^72^ was used to cluster sequences, with a length cut-off of 0.9 and sequence similarity of 0.9. Sequences were then independently aligned using MUSCLE v3.8.31 and manually trimmed in Geneious^73,74^. A maximum-likelihood phylogenetic tree was built from the alignment using IQ-TREE v1.6.12^75^ with the following parameters: -st AA -nt 48 -bb 1000 -m LG+G4+FO+I. Accession numbers used to construct the Cas13 phylogenetic tree are provided in Supplementary Table 6.

### Phage genome comparisons network

Protein-protein phage genome comparisons were performed with VConTACT2^76^ MCL clustering (rel-mode Diamond, vcs-mode ClusterONE) of the protein sequences of the Prokaryotic Viral RefSeq 201 phage database and the phages used during experiments in this study. Produced viral clusters that neither contained *E. coli* phage nor shared an edge with a viral cluster containing any *E. coli* phage were removed together with singletons to simplify the network.

Average nucleotide identity phage genome comparisons were performed with Gepard^77^ using a word length of 10 bp. For source genomes, we used a concatenation of the nine phage genomes used in this study: T4 (NC_000866.4), MM02 (MK373784.1), SUSP1 (NC_028808.2), EdH4 (MK327930.1), N4 (NC_008720.1), T7 (NC_001604.1), λ (J02459.1), T5 (NC_005859.1) and M13 (NC_003287.2).

### Reporting summary

Further information on research design is available in the Nature Research Reporting Summary linked to this article.

## Supplementary information

Supplementary Information Supplementary Figs. 1–37 and overview of Supplementary tables.

Reporting Summary

Supplementary Table Supplementary Tables 1–6.

Supplementary Data Source Data for Supplementary figures. Supplementary Figs. 1, 25, 26, 27, 28, 29, 36, 37 contain Excel-incompatible data formats.

## Data Availability

Cas13 entry, Cas13 negative control and homologous recombination plasmids are available from Addgene (addgene.org) (Addgene 186235–186247, 189580, 189582, 189584, 189587, 189589). All phage genome sequences, plasmids, oligonucleotides, gene fragments and DNA sequences can be found in Supplementary Tables 2–6, respectively. Source data are provided with this paper.

## Source data

Source Data Fig. 1 Unprocessed Newick format data (Excel format incompatible).
Source Data Fig. 2 Source Data for Fig. 2.
Source Data Fig. 3 Source data for Fig. 3a Network graph (Excel format incompatible).
Source Data Fig. 3 Source data for Fig. 3b Heat map.
Source Data Fig. 4 Source Data for Fig. 4.
Source Data Fig. 5 Source Data for Fig. 5.

## Code availability

None to report.
