# Supplementary material for: Broad-spectrum CRISPR-Cas13a enables efficient phage genome editing
Source: Nat Microbiol. 2022 Oct 31;7(12):1967–79. doi: 10.1038/s41564-022-01258-x (PMC9712115; doi:10.1038/s41564-022-01258-x)
Supplement: Supplementary file 1 — Supplementary Figs. 1–37 and overview of Supplementary tables. [file 41564_2022_1258_MOESM1_ESM.pdf]

nature microbiology

Article https://doi.org/10.1038/s41564-022-01258-x

Broad-spectrum CRISPR-Cas13a enables efficient phage genome editing

In the format provided by the authors and unedited

Broad-spectrum CRISPR-Cas13a enables efficient phage genome editing

Benjamin A Adler, Tomas Hessler, Brady F Cress, Arushi Lahiri, Vivek K. Mutalik, Rodolphe Barrangou, Jillian Banfield, Jennifer A Doudna

Supplementary Fig. 1. Unrooted graph of Cas13 phylogeny.

Supplementary Fig. 2. Summary of results for LbuCas13a and RfxCas13d interference against phage T4.

Supplementary Fig. 3. Growth curves of Cas13 negative control guides.

Supplementary Fig. 4. Spacer-associated toxicity more pronounced for RfxCas13d.

Supplementary Fig. 5. Genetic diagram of the native RfxCas13d locus.

Supplementary Fig. 6. Phage-restriction LbuCas13a-crRNAs are scattered across diverse phage genomes.

Supplementary Fig. 7. Nucleotide similarity of phages targeted in this study.

Supplementary Fig. 8. Summary of results for LbuCas13a interference against phage EdH4.

Supplementary Fig. 9. Summary of results for LbuCas13a interference against phage MM02.

Supplementary Fig. 10. Summary of results for LbuCas13a interference against phage λ.

Supplementary Fig. 11. Summary of results for LbuCas13a interference against ssDNA(+) phage M13.

Supplementary Fig. 12. Summary of results for LbuCas13a interference against phage N4.

Supplementary Fig. 13. Summary of results for LbuCas13a interference against phage SUSP1.

Supplementary Fig. 14. Summary of results for LbuCas13a interference against phage T5.

Supplementary Fig. 15. Summary of results for LbuCas13a interference against phage T7.

Supplementary Fig. 16. Restoration of LbuCas13a interference against T5*pol* with eLbuCas13a due to reduced autotoxicity.

Supplementary Fig. 17. SUSP1 displays sensitivity to LbuCas13a targeting during liquid infection.

Supplementary Fig. 18. Method overview for Cas13a Phage Editing.

Supplementary Fig. 19. Elevated auto-toxicity observed with EdH4_gp004_CDS_2 spacer.

Supplementary Fig. 20. Overview of phenotypic results for editing attempt T4Δ*soc*.

Supplementary Fig. 21. Overview of phenotypic results for editing attempt T4wtGT7.

Supplementary Fig. 22. Overview of phenotypic results for editing attempt T7Δ*gp1.7*.

Supplementary Fig. 23. Overview of phenotypic results for editing attempt EdH4Δ*gp004*.

Supplementary Fig. 24. Overview of phenotypic results for editing attempt EdH4Δ*gp214*.

Supplementary Fig. 25. Overview of genotyping results for editing attempt T4Δ*soc*.

Supplementary Fig. 26. Overview of genotyping results for editing attempt T4wtGT7.

Supplementary Fig. 27. Overview of genotpying results for editing attempt T7Δ*gp1.7*.

Supplementary Fig. 28. Overview of genotyping results for editing attempt EdH4Δ*gp004*.

Supplementary Fig. 29. Overview of genotyping results for editing attempt EdH4Δ*gp214*.

Supplementary Fig. 30. Overview of phenotypic results for editing attempt *soc*-C.

Supplementary Fig. 31. Overview of phenotypic results for editing attempt *soc*-S.

Supplementary Fig. 32. Overview of phenotypic results for editing attempt *soc*-F.

Supplementary Fig. 33. Overview of phenotypic results for editing attempt *dnap*-C.

Supplementary Fig. 34. Overview of phenotypic results for editing attempt *dnap*-S.

Supplementary Fig. 35. Overview of phenotypic results for editing attempt *dnap*-F.

Supplementary Fig. 36. Overview of genotyping results for *soc* editing attempts.

Supplementary Fig. 37. Overview of genotyping results for *dnap* editing attempts.

Supplementary Table 1 - Summary of crRNA Design

Supplementary Table 2 - Summary of crRNAs Used in Cas13a Phage Restriction Assays

Supplementary Table 3 - Plasmids Used in This Study

Supplementary Table 4 - Oligonucleotides Used in This Study

Supplementary Table 5 - Gene Fragments Used in this Study

Supplementary Table 6 - Cas13 Phylogeny Source Sequences

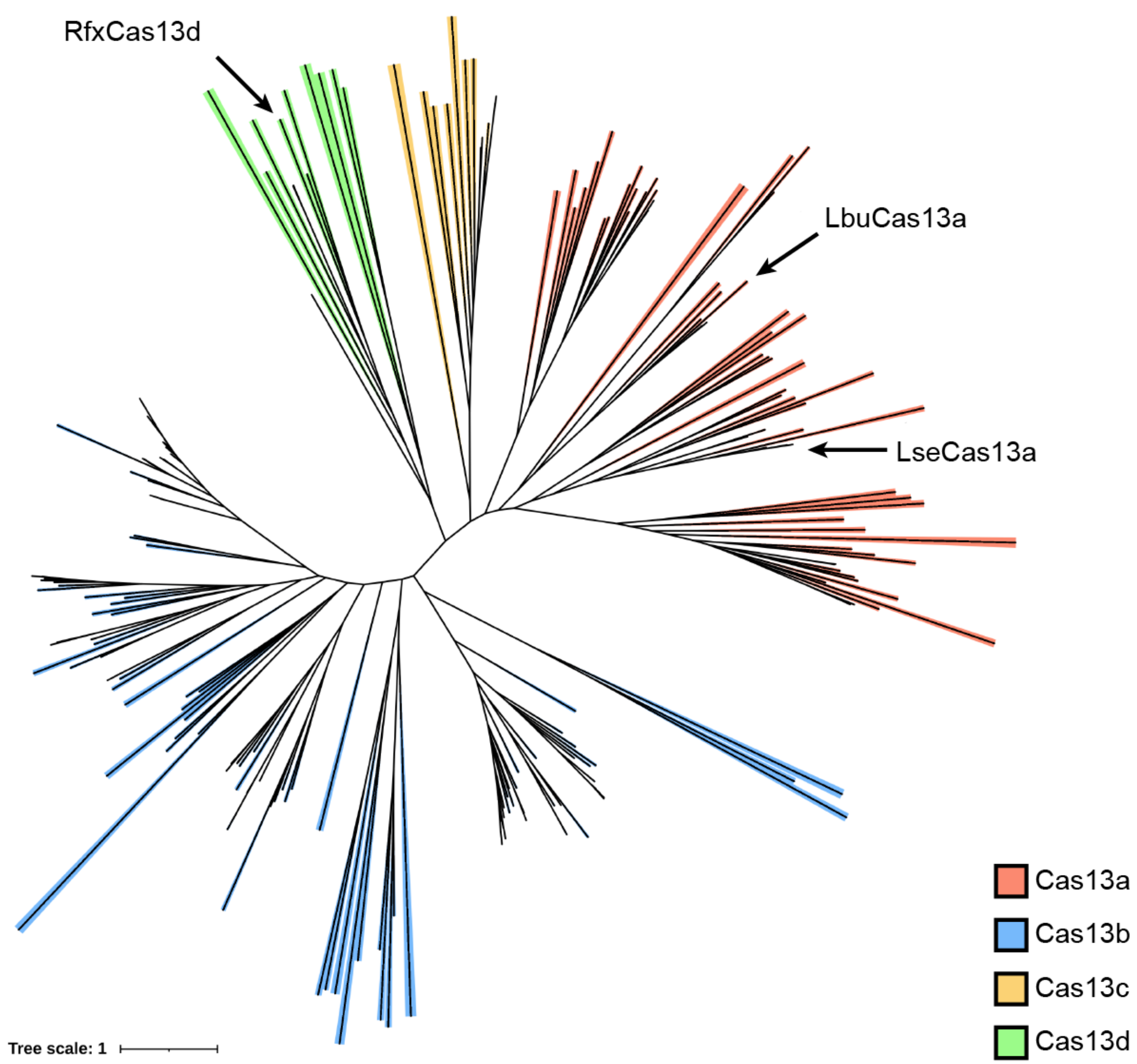

Supplementary Fig. 1. Unrooted graph of Cas13 phylogeny. Unrooted tree of Cas13 effectors colored by subtype. Cas13 effectors from this study (LbuCas13a, RfxCas13d) and prior studies (LseCas13a) investigating anti-phage activity are shown.

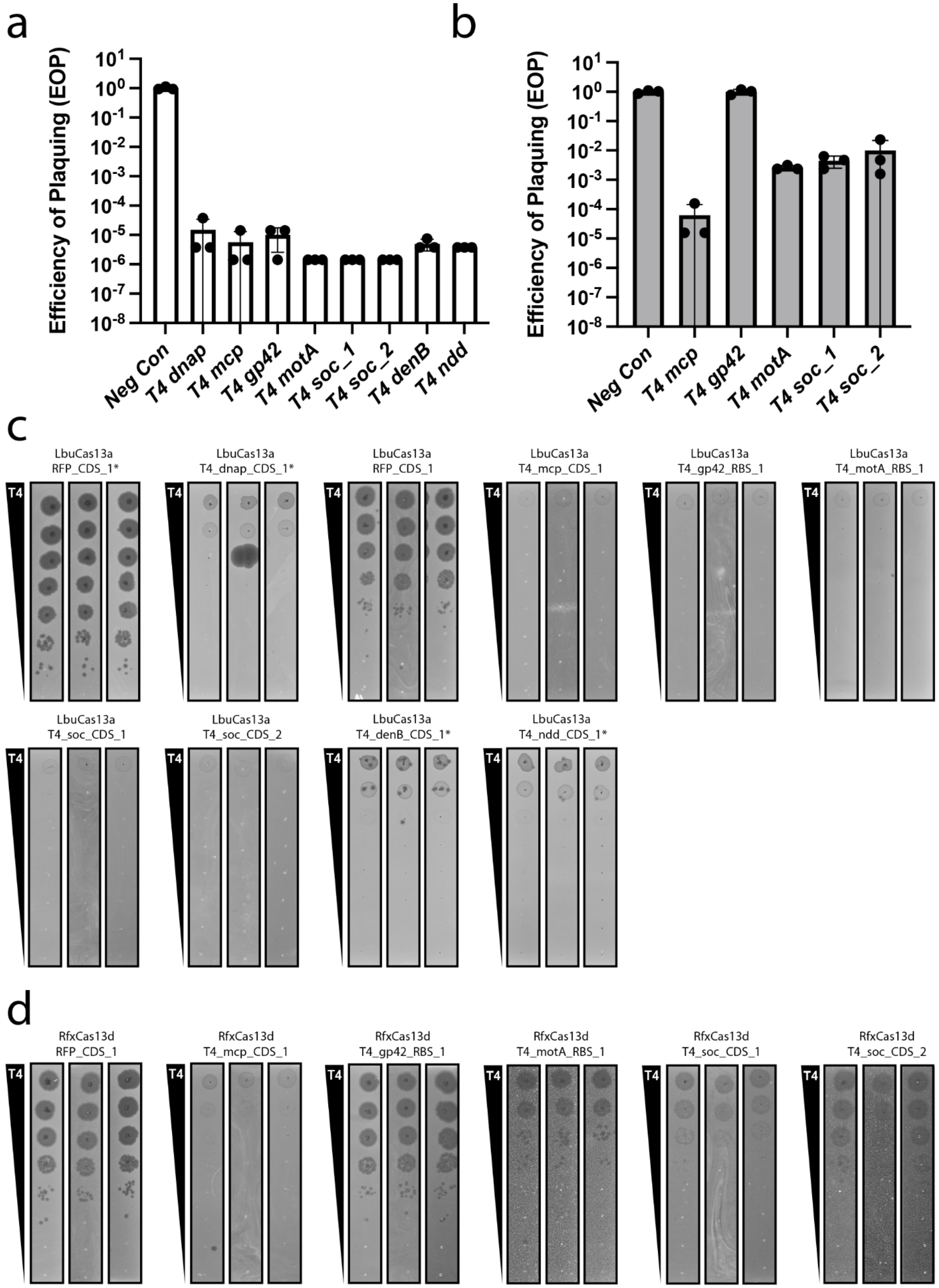
a
Efficiency of Plaquing (EOP)
10^1
10^0
10^-1
10^-2
10^-3
10^-4
10^-5
10^-6
10^-7
10^-8
Neg Con
T4 dnap
T4 mcp
T4 gp42
T4 motA
T4 soc_1
T4 soc_2
T4 denB
T4 ndd
b
Efficiency of Plaquing (EOP)
Neg Con
T4 mcp
T4 gp42
T4 motA
T4 soc_1
T4 soc_2
c
LbuCas13a RFP_CDS_1*
LbuCas13a T4_dnap_CDS_1*
LbuCas13a RFP_CDS_1
LbuCas13a T4_mcp_CDS_1
LbuCas13a T4_gp42_RBS_1
LbuCas13a T4_motA_RBS_1
LbuCas13a T4_soc_CDS_1
LbuCas13a T4_soc_CDS_2
LbuCas13a T4_denB_CDS_1*
LbuCas13a T4_ndd_CDS_1*
T4
d
RfxCas13d RFP_CDS_1
RfxCas13d T4_mcp_CDS_1
RfxCas13d T4_gp42_RBS_1
RfxCas13d T4_motA_RBS_1
RfxCas13d T4_soc_CDS_1
RfxCas13d T4_soc_CDS_2
T4

Supplementary Fig. 2. Summary of results for LbuCas13a and RfxCas13d interference against phage T4. (a) EOP estimation for T4 infection against all crRNAs tested at 5 nM aTc induction of LbuCas13a. (b) EOP estimation for T4 infection against all crRNAs tested at 5 nM aTc induction of RfxCas13d. (c) Plaque assay results for T4 infection against all crRNAs tested at 5 nM aTc induction of LbuCas13a. Experiments denoted with a * refer to a different preparation of T4 lysate. (d) Plaque assay results for T4 infection against all crRNAs tested at 5 nM aTc induction of RfxCas13d. For EOP graphs, results are represented as mean values ± SD. “Neg Con” in EOP graphs refers to non-targeting spacer control RFP_CDS_1. All assays were performed in biological triplicate.

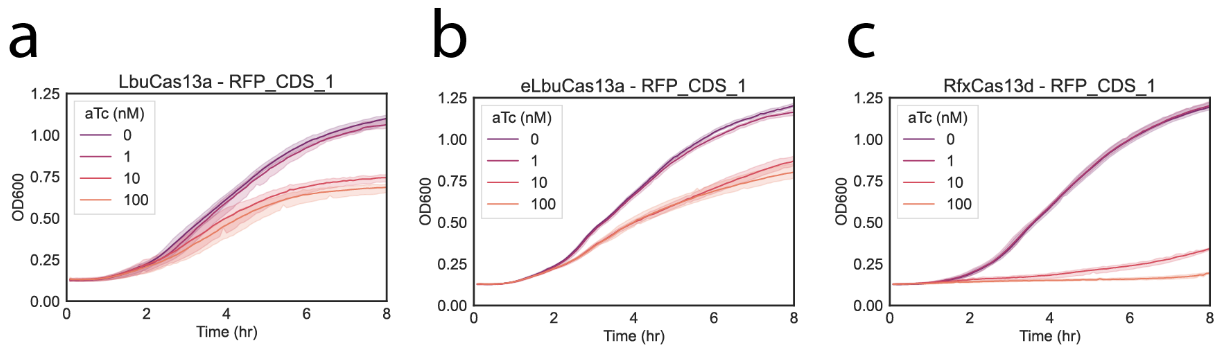

Supplementary Fig. 3. Growth curves of Cas13 negative control guides. Growth curves of *E. coli* expressing (a) LbuCas13a, (b) eLbuCas13a, and (c) RfxCas13d with increasing induction (0, 1, 10, 100 nM aTc) in conjunction with a RFP-targeting negative control spacer. A given spacer target reflects the same sequence between LbuCas13a, eLbuCas13a, and RfxCas13d. All assays were performed with 3 biological replicates. Data are plotted using mean values surrounded by the 95%CI.

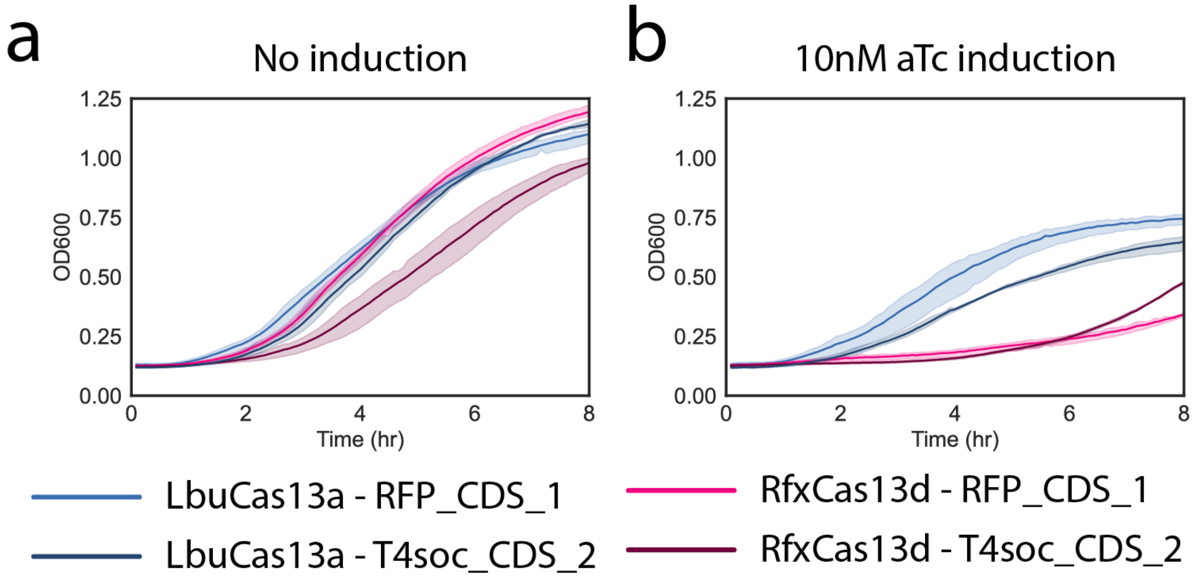

Supplementary Fig. 4. Spacer-associated toxicity more pronounced for RfxCas13d. Growth curves of *E. coli* strains expressing LbuCas13a or RfxCas13d with crRNAs targeting either RFP or T4*soc* at (a) 0 or (b) 10nM aTc induction. A given spacer target reflects the same sequence between LbuCas13a and RfxCas13d. All assays were performed with 3 biological replicates. Data are plotted using mean values surrounded by the 95%CI.

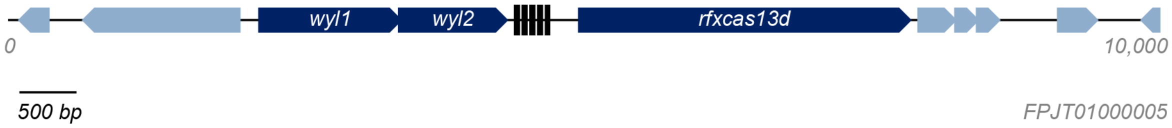

Supplementary Fig. 5. Genetic diagram of the native RfxCas13d locus. Gene diagram overview of RfxCas13d's native locus (FPJT01000005 (0-10,000bp)). RfxCas13d and potential auxiliary split WYL-domain-containing effector genes are highlighted in dark blue. CRISPR direct repeats are shown in black.

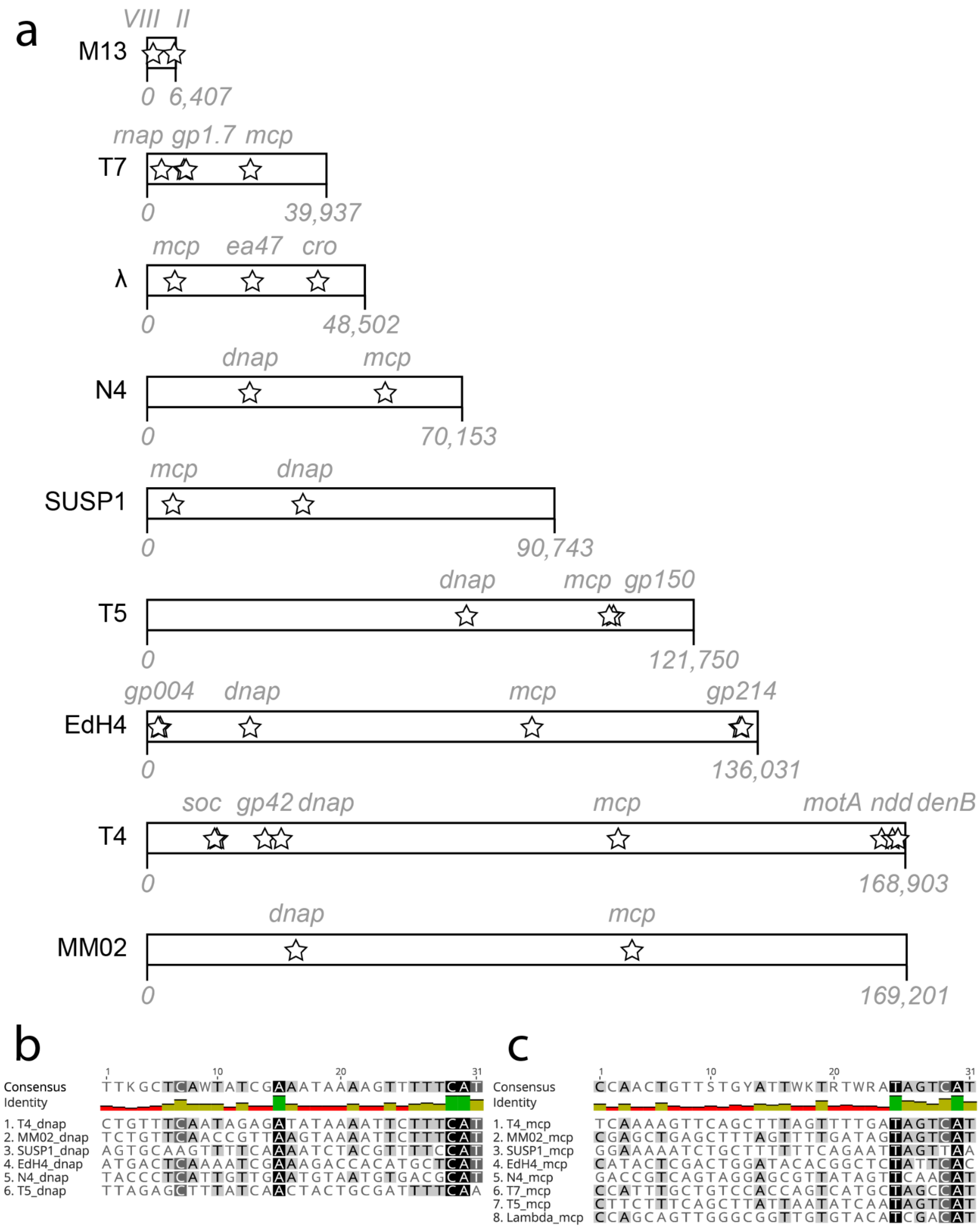

Supplementary Fig. 6. Phage-restriction LbuCas13a-crRNAs are scattered across diverse phage genomes. (a) Summary of phage's genomes used in this study and locations of

LbuCas13a crRNA targets (stars) mapped to scale. Target transcript's closest gene is annotated. (b) Only distant similarity observed between spacers targeting the same gene, *dnap* across the dsDNA phages investigated in this study. (c) Only distant similarity observed between spacers targeting the same gene, *mcp* across the dsDNA phages investigated in this study.

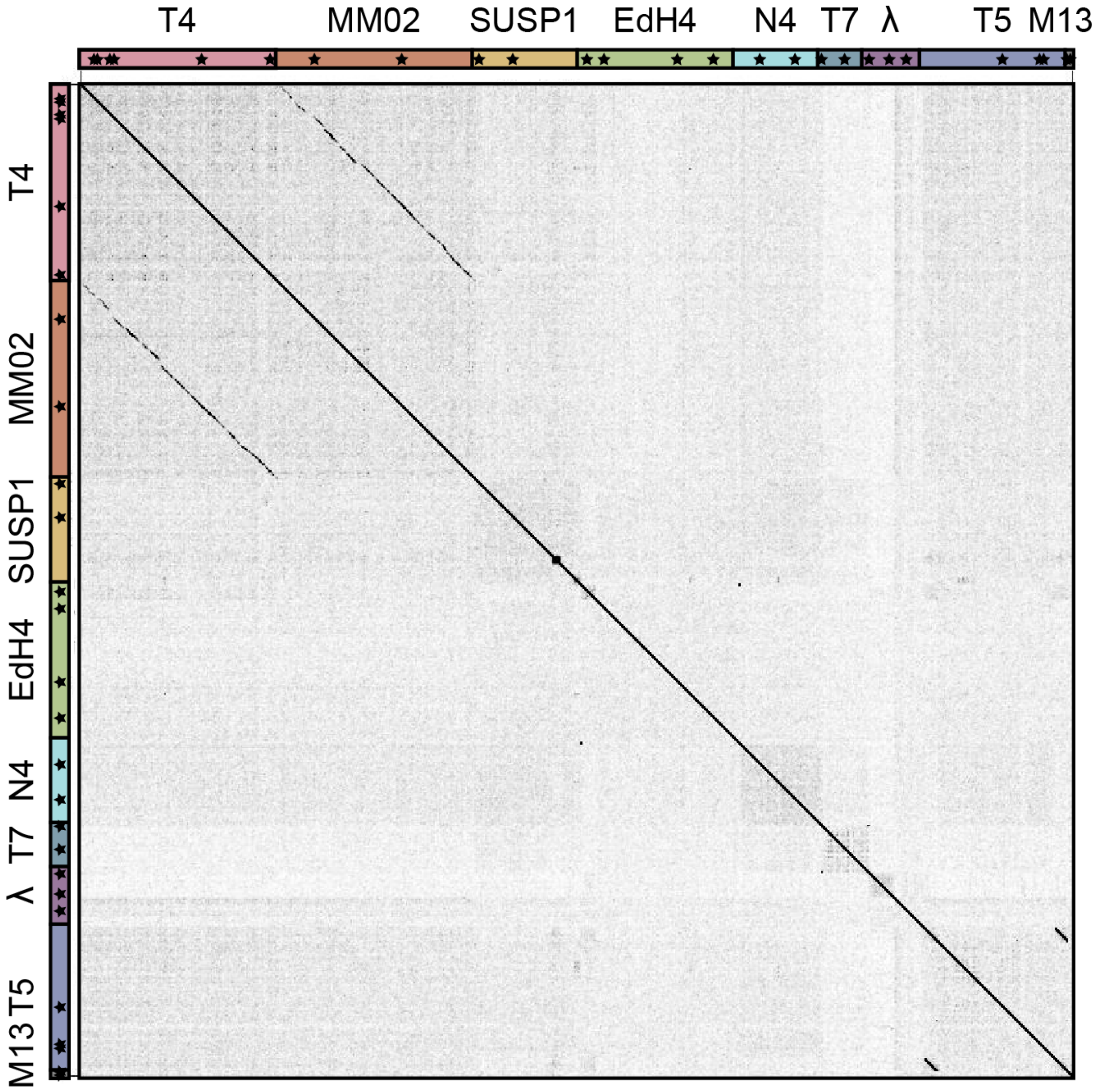

Supplementary Fig. 7. Nucleotide similarity of phages targeted in this study. Gepard dot plot comparing the average nucleotide identity of phage genomes and the location of crRNAs used in this study. Higher regions of similarity are shown with darker shade. Phage genomes are concatenated and annotated on the axes.

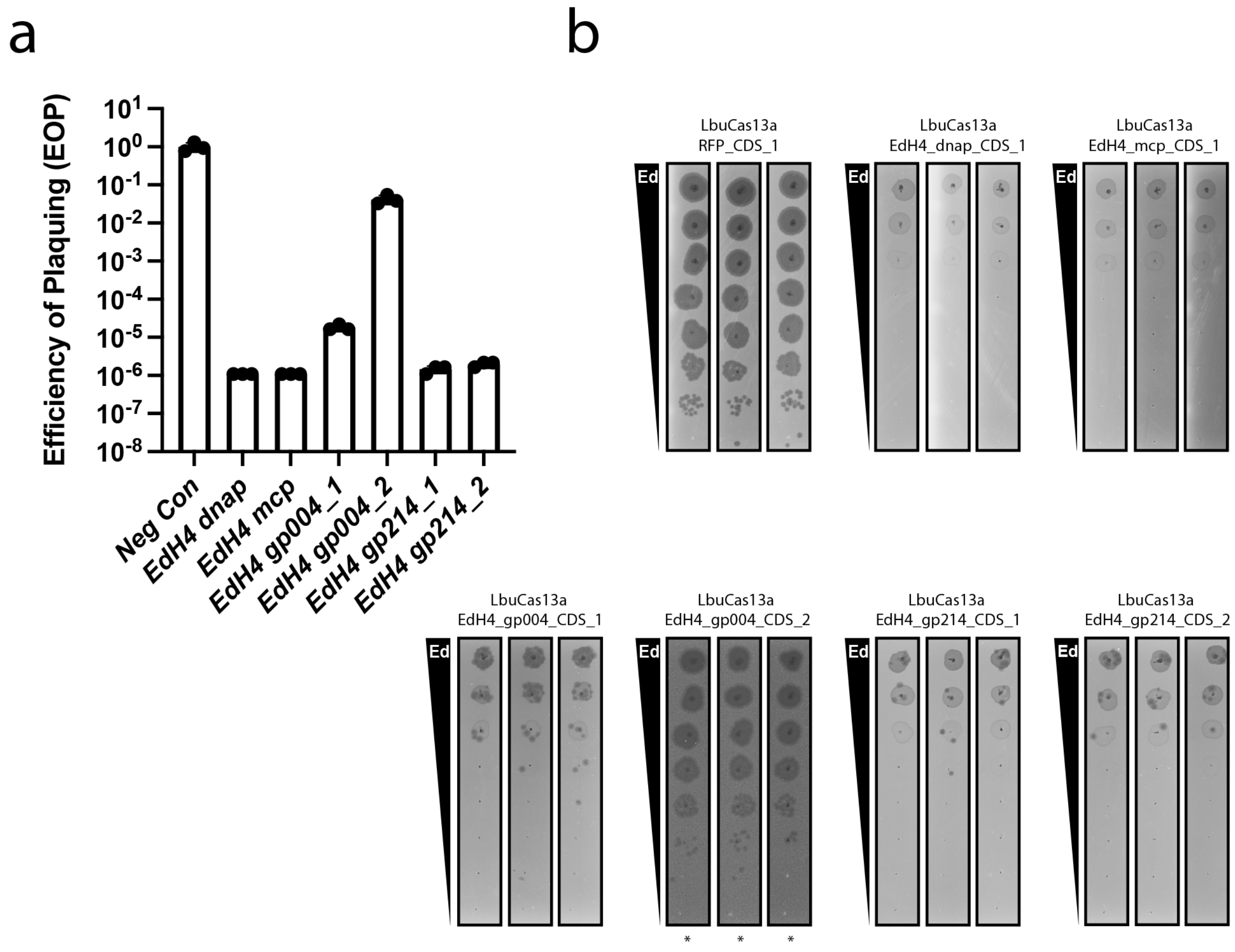

Supplementary Fig. 8. Summary of results for LbuCas13a interference against phage EdH4. (a) EOP estimation and (b) plaque assay results for EdH4 infection against all crRNAs tested at 5 nM aTc induction of LbuCas13a. For EOP graphs, results are represented as mean values ± SD. “Neg Con” in EOP graphs refers to non-targeting spacer control RFP_CDS_1. All assays were performed in biological triplicate.

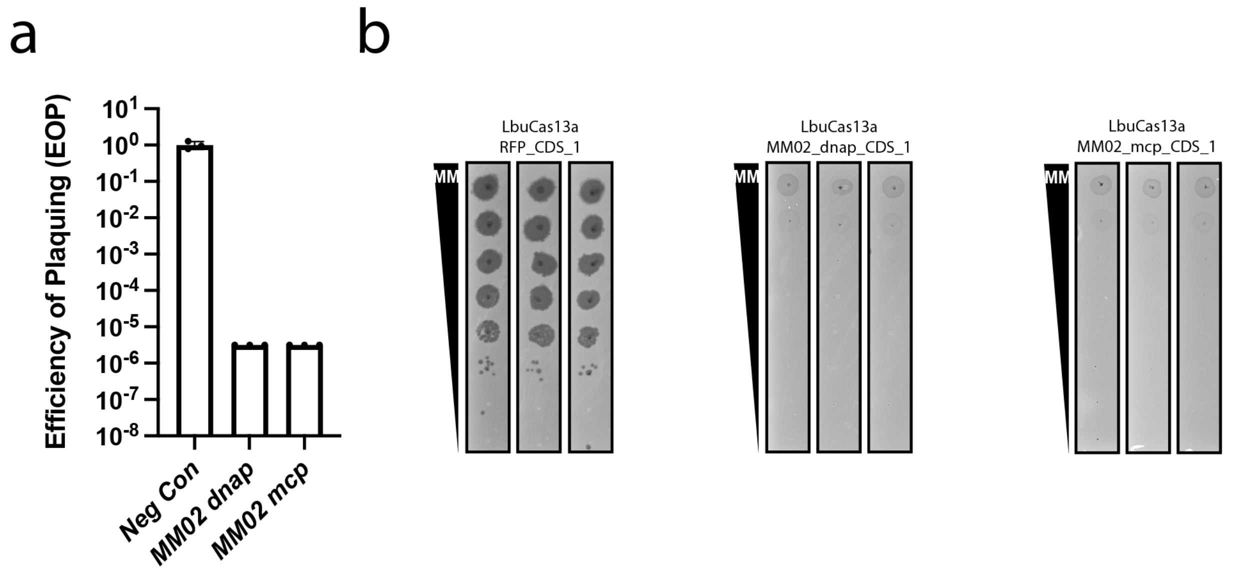

Supplementary Fig. 9. Summary of results for LbuCas13a interference against phage MM02. (a) EOP estimation and (b) plaque assay results for MM02 infection against all crRNAs tested at 5 nM aTc induction of LbuCas13a. “Neg Con” in EOP graphs refers to non-targeting spacer control RFP_CDS_1. For EOP graphs, results are represented as mean values ± SD. All assays were performed in biological triplicate.

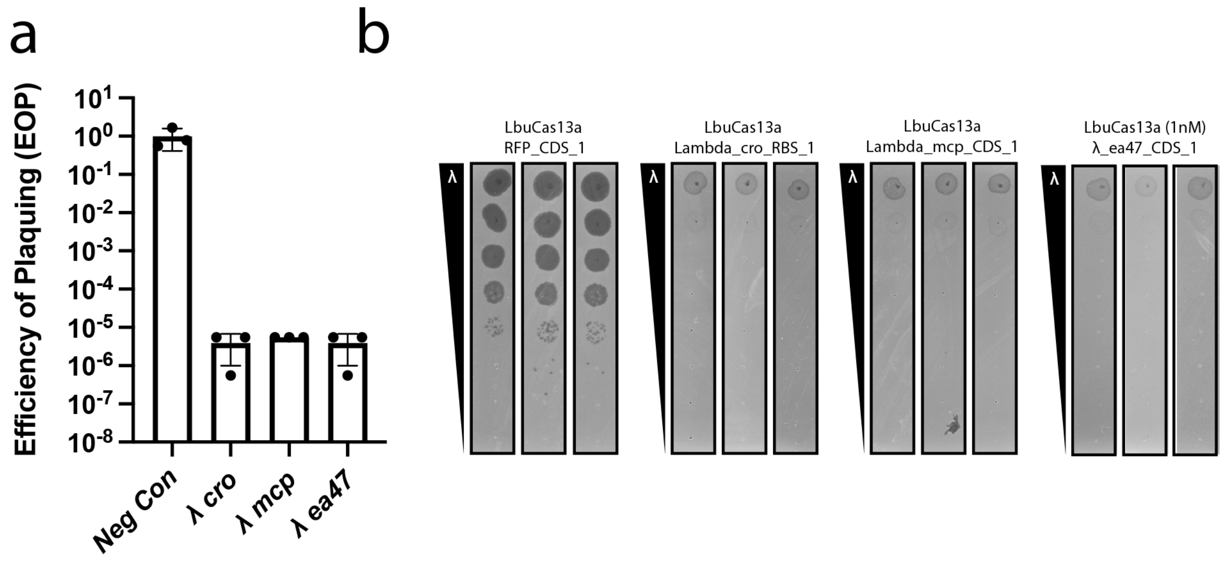

Supplementary Fig. 10. Summary of results for LbuCas13a interference against phage λ. (a) EOP estimation and (b) plaque assay results for λ infection against all crRNAs tested at 5 nM aTc induction of LbuCas13a. Due to toxicity at 5 nM aTc induction with LbuCas13a, crRNA Lambda_ea47_CDS_1 assays were performed at 1 nM aTc. “Neg Con” in EOP graphs refers to non-targeting spacer control RFP_CDS_1. For EOP graphs, results are represented as mean values ± SD. All assays were performed in biological triplicate.

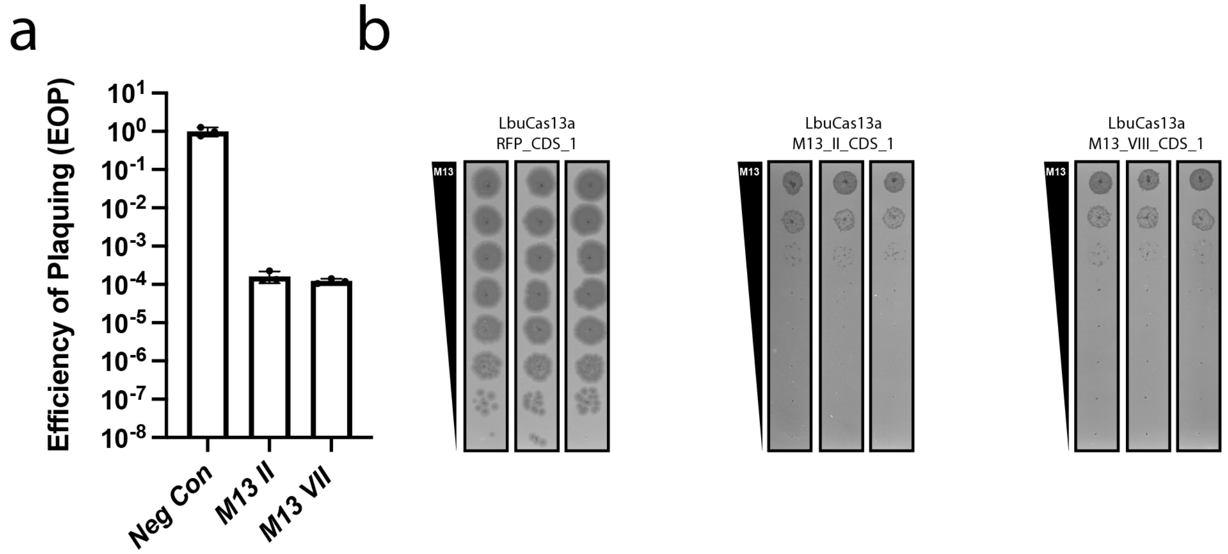

Supplementary Fig. 11. Summary of results for LbuCas13a interference against ssDNA(+) phage M13. (a) EOP estimation and (b) plaque assay results for M13 infection against all crRNAs tested at 5 nM aTc induction of LbuCas13a. “Neg Con” in EOP graphs refers to non-targeting spacer control RFP_CDS_1. For EOP graphs, results are represented as mean values ± SD. All assays were performed in biological triplicate.

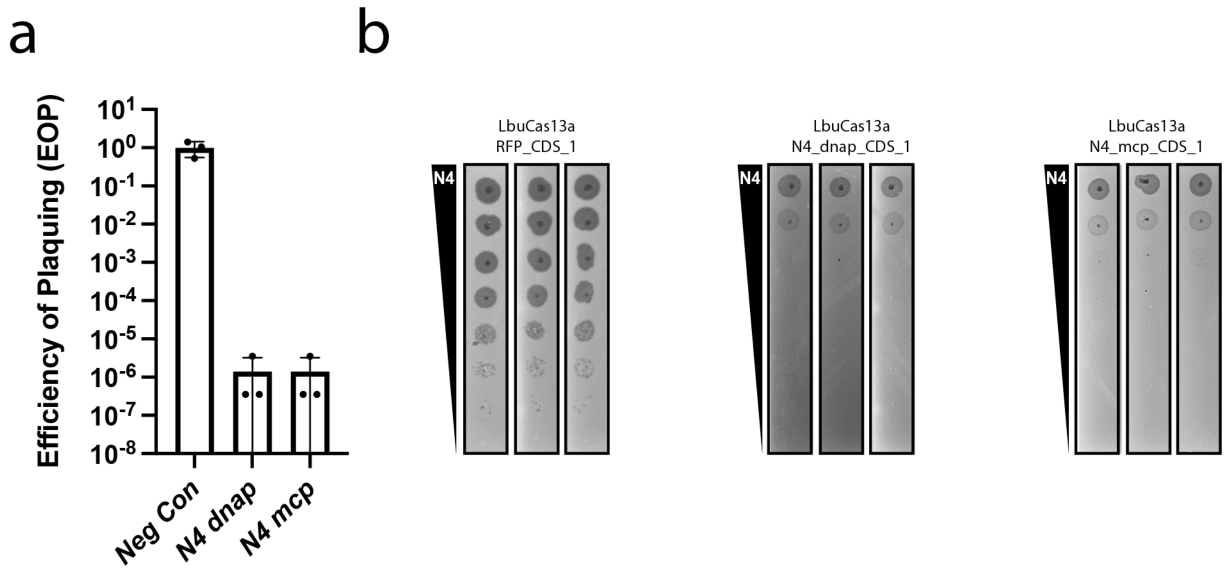

Supplementary Fig. 12. Summary of results for LbuCas13a interference against phage N4. (a) EOP estimation and (b) plaque assay results for N4 infection against all crRNAs tested at 5 nM aTc induction of LbuCas13a. “Neg Con” in EOP graphs refers to non-targeting spacer control RFP_CDS_1. For EOP graphs, results are represented as mean values ± SD. All assays were performed in biological triplicate.

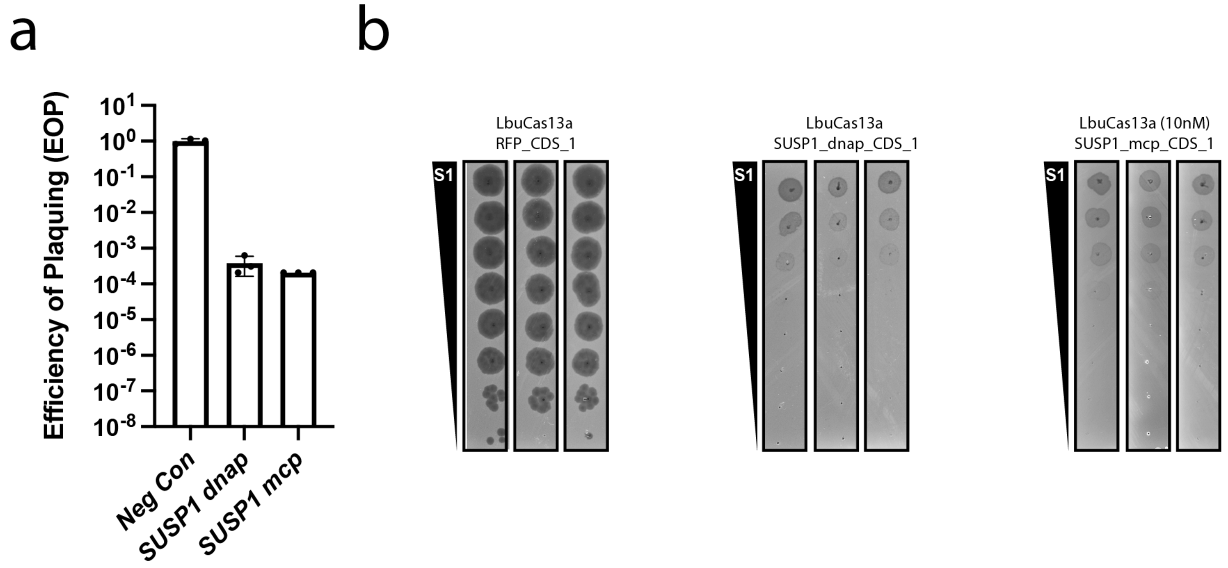

Supplementary Fig. 13. Summary of results for LbuCas13a interference against phage SUSP1. (a) EOP estimation and (b) plaque assay results for SUSP1 infection against all crRNAs tested at 5 nM aTc induction of LbuCas13a.“Neg Con” in EOP graphs refers to non-targeting spacer control RFP_CDS_1. For EOP graphs, results are represented as mean values ± SD. All assays were performed in biological triplicate.

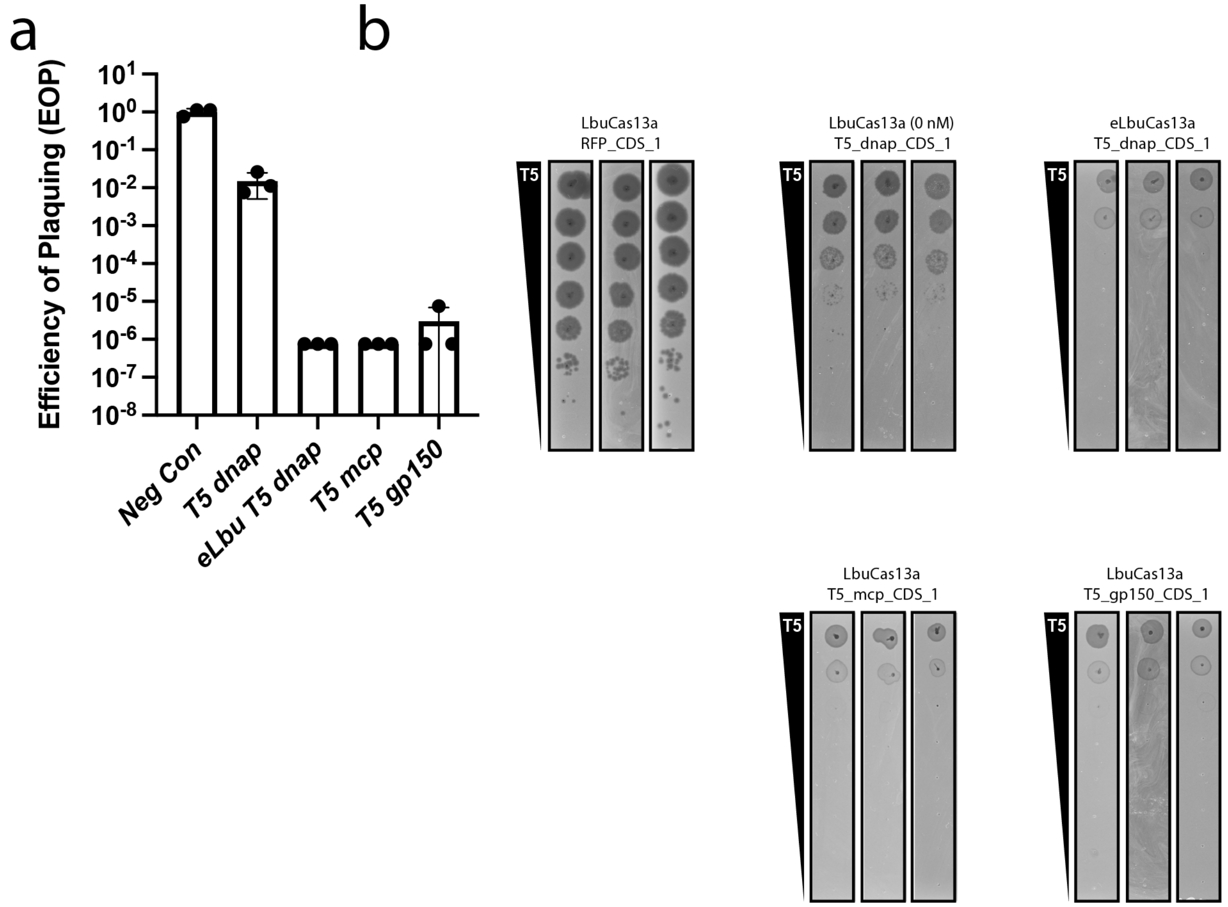

Supplementary Fig. 14. Summary of results for LbuCas13a interference against phage T5. (a) EOP estimation and (b) plaque assay results for T5 infection against all crRNAs tested at 5 nM aTc induction of LbuCas13a unless stated otherwise. Due to toxicity at 5 nM aTc induction with LbuCas13a, crRNA T5_dnap_CDS_1 assays were performed at 0 nM aTc. Additionally crRNA_dnap_CDS_1 was cloned into a reduced toxicity variant of LbuCas13a referred to as eLbuCas13a (Methods), which is also shown at 5 nM aTc induction.“Neg Con” in EOP graphs refers to non-targeting spacer control RFP_CDS_1. For EOP graphs, results are represented as mean values ± SD. All assays were performed in biological triplicate.

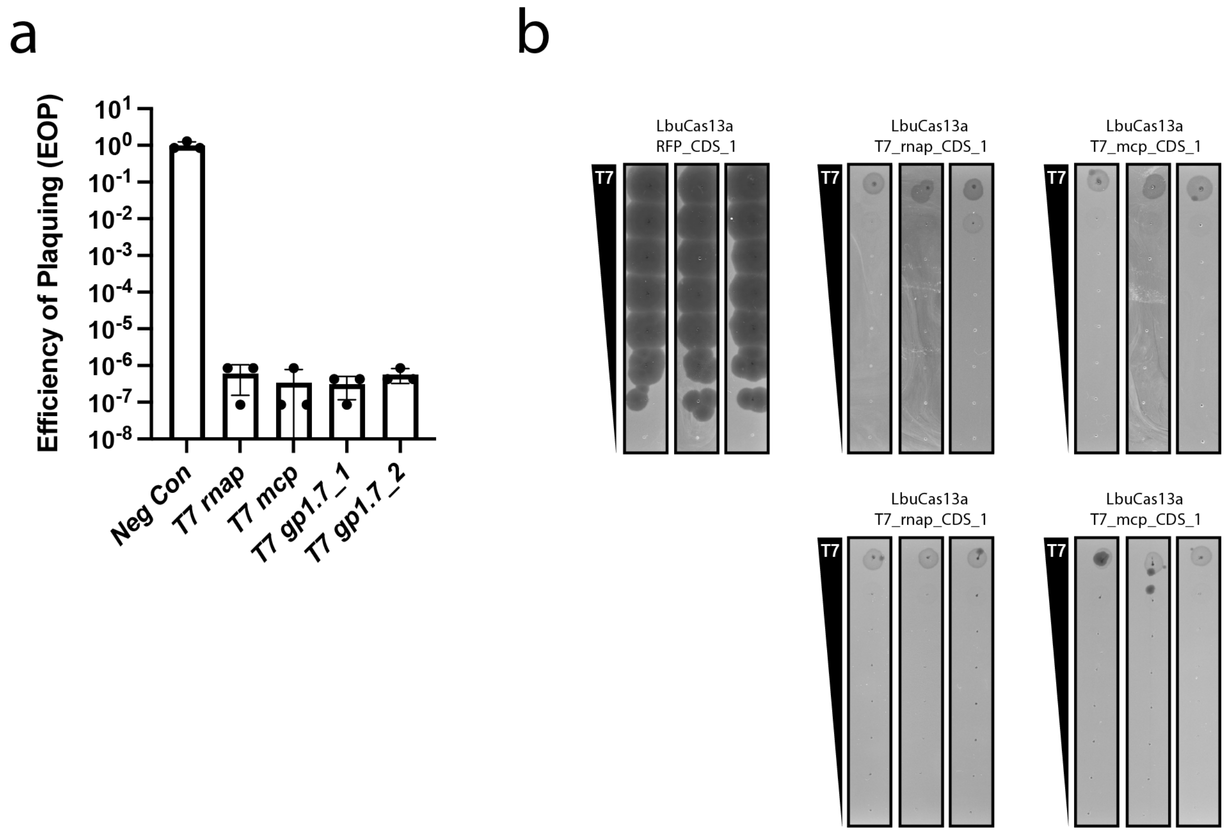

Supplementary Fig. 15. Summary of results for LbuCas13a interference against phage T7. (a) EOP estimation and (b) plaque assay results for T7 infection against all crRNAs tested at 5 nM aTc induction of LbuCas13a.“Neg Con” in EOP graphs refers to non-targeting spacer control RFP_CDS_1. For EOP graphs, results are represented as mean values ± SD. All assays were performed in biological triplicate.

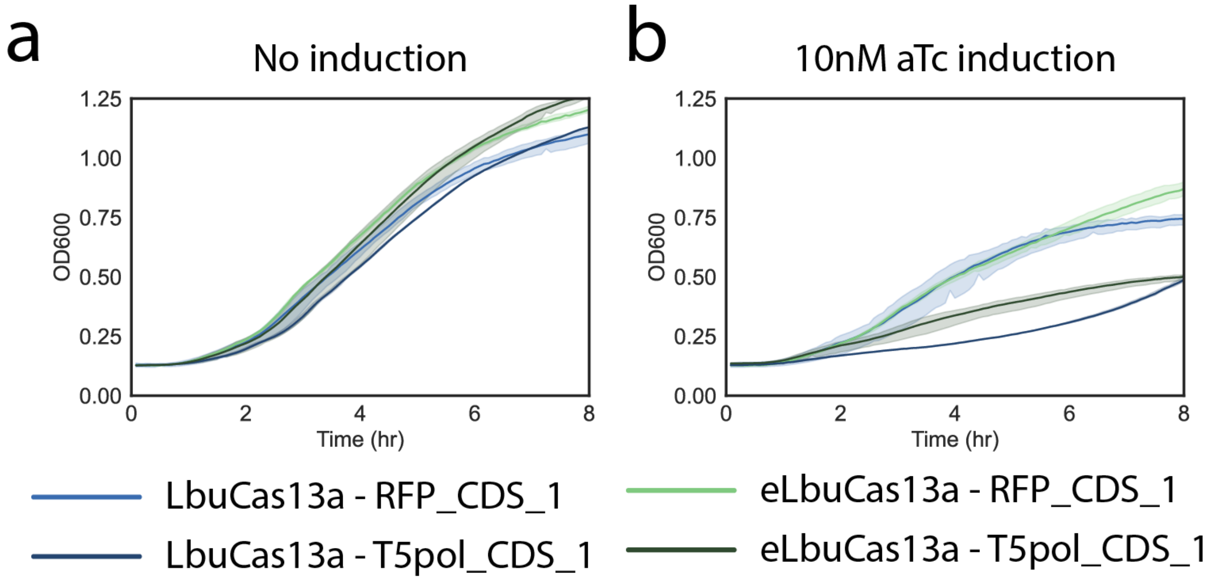

Supplementary Fig. 16. Restoration of LbuCas13a interference against T5*pol* with eLbuCas13a due to reduced autotoxicity. Growth curves of *E. coli* strains expressing LbuCas13a or eLbuCas13a with crRNAs targeting either RFP or T5*pol* at (a) 0 or (b) 10nM aTc induction and in the absence of phage infection. A given spacer target reflects the same sequence between LbuCas13a and eLbuCas13a. All assays were performed with 3 biological replicates. Data are plotted using mean values surrounded by the 95%CI.

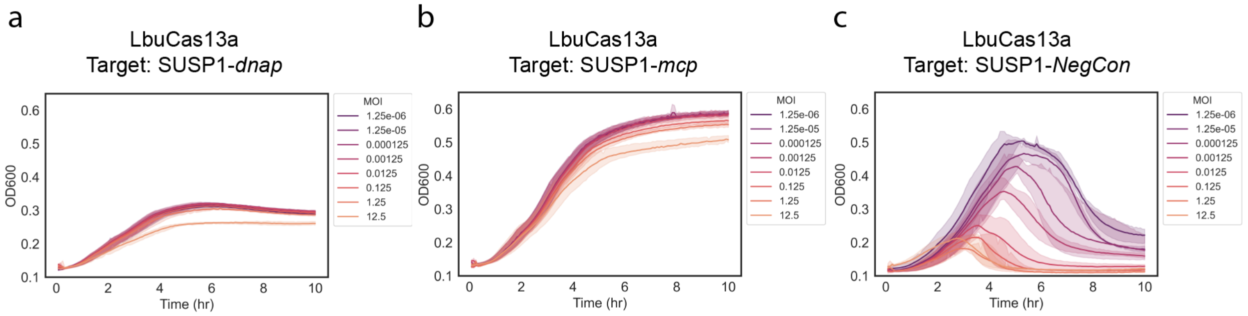

Supplementary Fig. 17. SUSP1 displays sensitivity to LbuCas13a targeting during liquid infection. Liquid infection experiments for phage SUSP1 against *E. coli* expressing LbuCas13a and a crRNA targeting (a) SUSP1*dnap*, (b) SUSP1*mcp*, and (c) a non-targeting crRNA across a range of MOIs. All assays were performed at 10 nM aTc induction with 3 biological replicates. Data are plotted using mean values surrounded by the 95%CI.

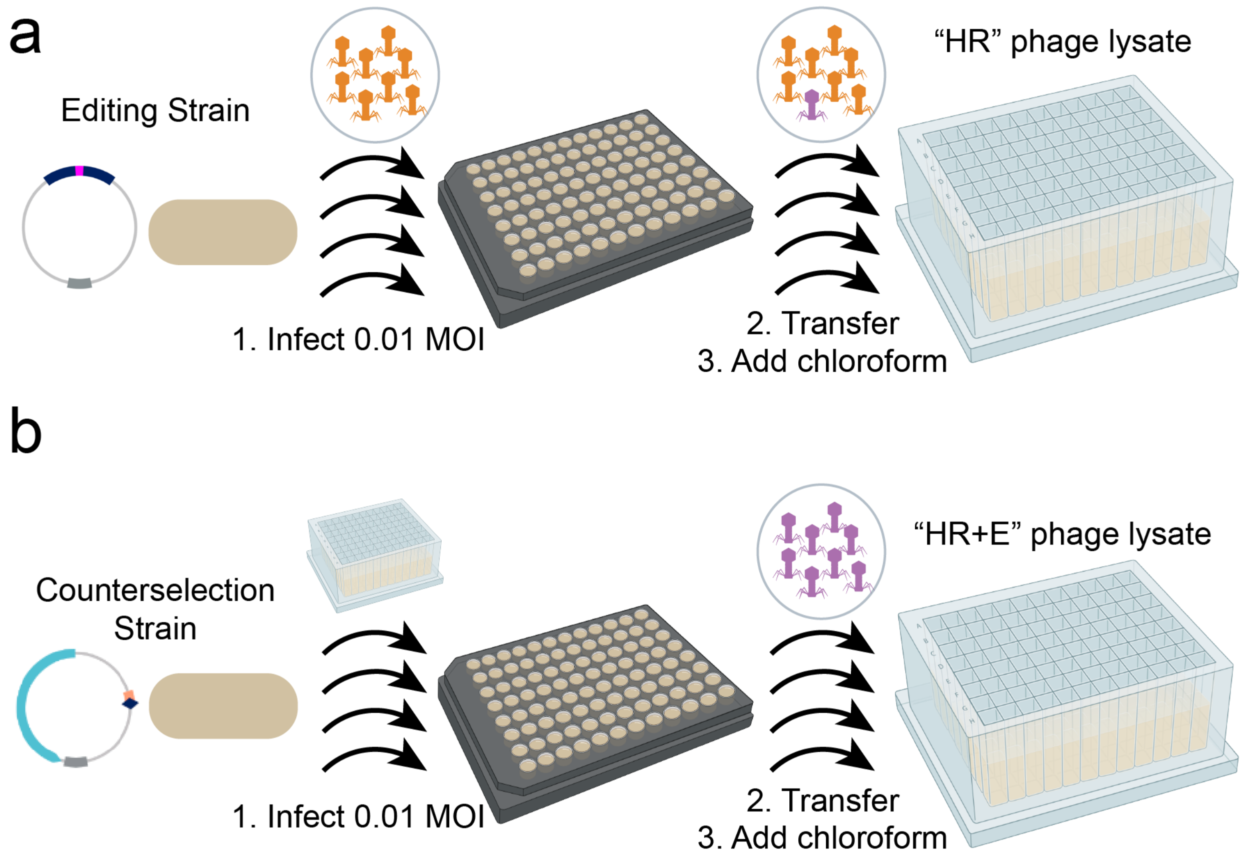

Supplementary Fig. 18. Method overview for Cas13a Phage Editing. (a) Homologous Recombination edited (HR) phage lysate is created by infection of an editing strain at 0.01 MOI. After lysis is observed phage lysates comprising a mixture of edited (purple) and wildtype (orange) phage are transferred to a deep well block and a drop of chloroform is added. These are the "HR" phage lysates. (b) Enriched phage lysates are created by infection of an counterselection strain at 0.01 MOI from an "HR" phage lysate. After lysis is observed phage lysates comprising primarily of edited (purple) phage are transferred to a deep well block and a drop of chloroform is added. These are the "HR+E" phage lysates.

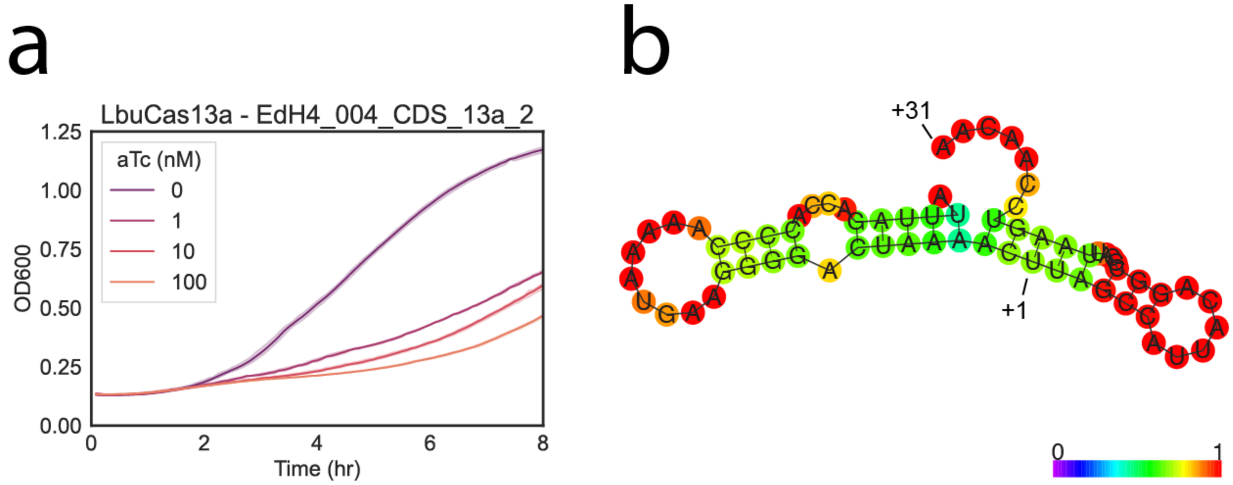

Supplementary Fig. 19. Elevated auto-toxicity observed with EdH4_gp004_CDS_2 spacer. (a) Growth curves of *E. coli* strains expressing LbuCas13a with crRNAs targeting EdH4_gp004_CDS_2 at varying levels of induction (0nM, 1nM, 10nM, 100nM aTc. All assays were performed with 3 biological replicates. (b) RNAfold on the crRNA used reveals extensive secondary structure early in the mature spacer. +1 site and +31 site of the spacer is noted. Base pairing probabilities of the centroid structure produced by ViennaRNA are shown. Data are plotted using mean values surrounded by the 95%CI.

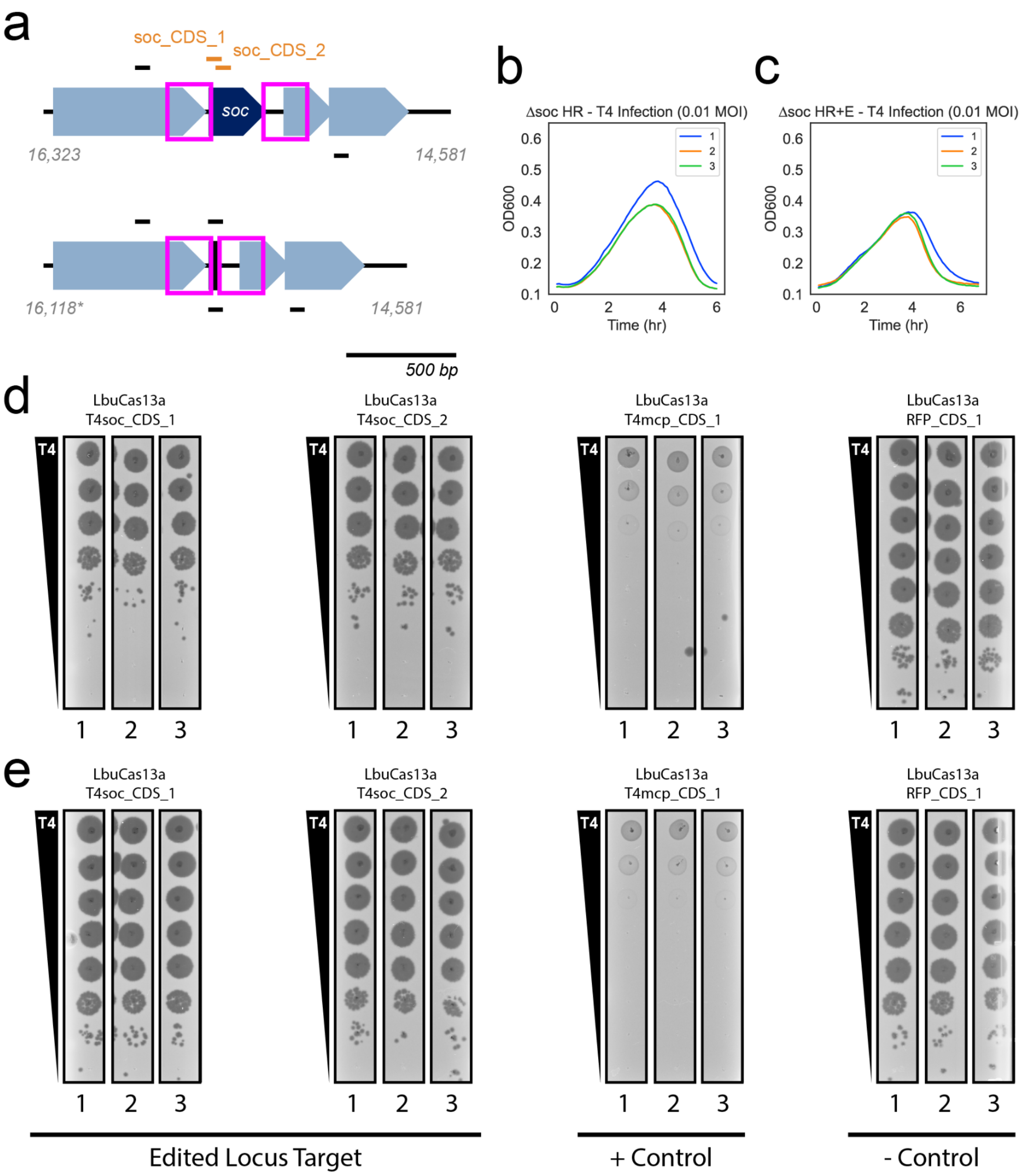

Supplementary Fig. 20. Overview of phenotypic results for editing attempt T4Δ*soc*. (a) Gene diagram overview of T4*soc* wildtype locus (top) and edited locus (bottom). *soc* gene is highlighted in dark blue. Homology arms are outlined in magenta. Approximate locations of counterselection protospacers are shown in orange. Primer binding sites for PCR verification

are shown in black. Gene coordinates that change between wildtype and edited phage are marked with an asterisk. (b) Growth curves for the homologous recombination editing step for T4Δ*soc*. (c) Growth curves for the Cas13a counterselection step for T4Δ*soc*. (d) Plaquing assay for HR lysates against *E. coli* expressing LbuCas13a and crRNA targeting *soc* (left, mid-left), *mcp* (mid-right), or RFP (right). (e) Plaquing assay for HR+E lysates against *E. coli* expressing LbuCas13a and crRNA targeting *soc* (left, mid-left), *mcp* (mid-right), or RFP (right). For all liquid culture assays, induction was performed with 10 nM aTc. For all plate-based assays shown using Cas13a, induction was performed with 5 nM aTc. Labels 1-3 correspond to unique parallel editing workflows.

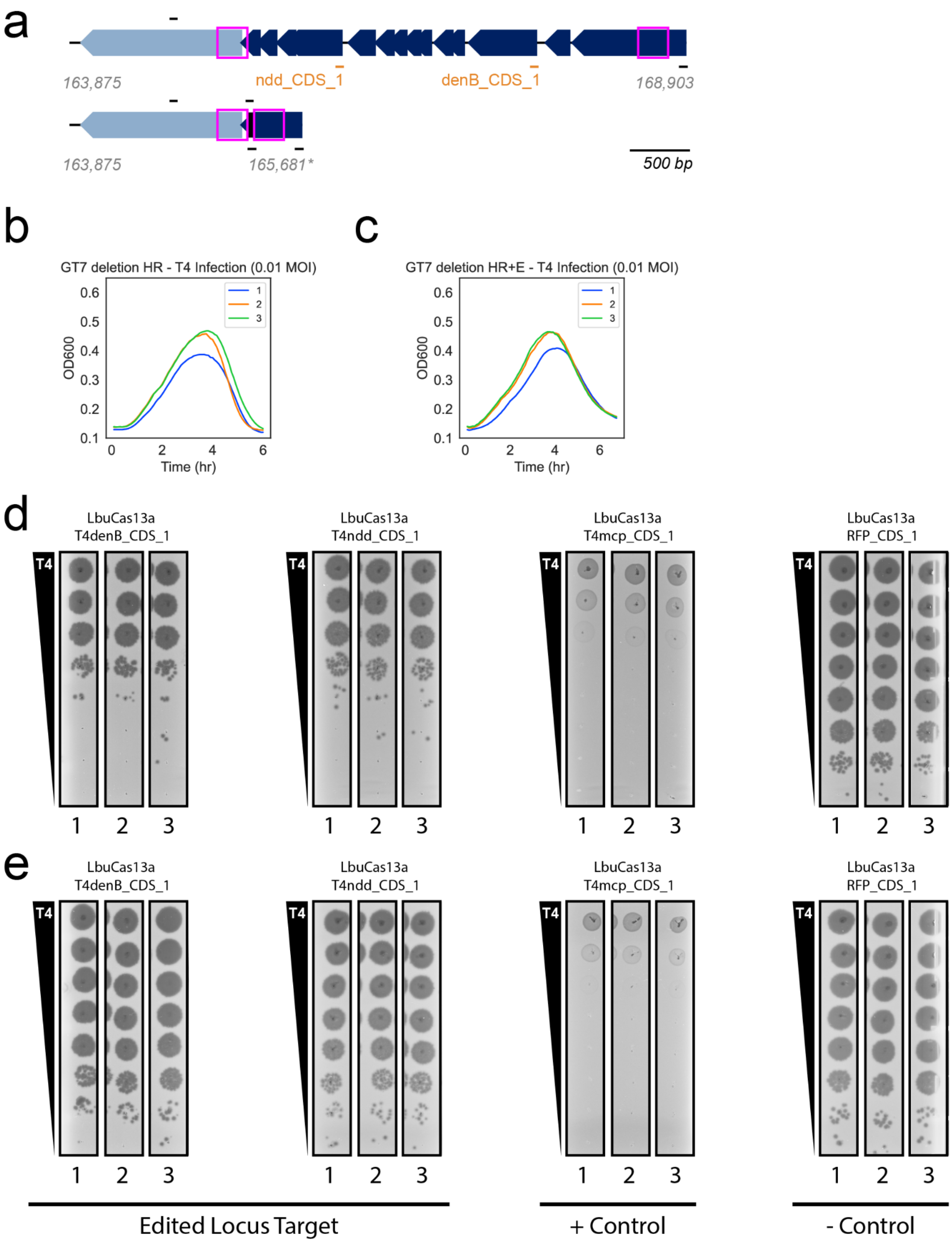
a
163,875
ndd_CDS_1
denB_CDS_1
168,903
163,875
165,681*
500 bp
b
GT7 deletion HR - T4 Infection (0.01 MOI)
OD600
Time (hr)
c
GT7 deletion HR+E - T4 Infection (0.01 MOI)
OD600
Time (hr)
d
LbuCas13a
T4denB_CDS_1
LbuCas13a
T4ndd_CDS_1
LbuCas13a
T4mcp_CDS_1
LbuCas13a
RFP_CDS_1
T4
e
LbuCas13a
T4denB_CDS_1
LbuCas13a
T4ndd_CDS_1
LbuCas13a
T4mcp_CDS_1
LbuCas13a
RFP_CDS_1
T4
Edited Locus Target
+ Control
- Control

Supplementary Fig. 21. Overview of phenotypic results for editing attempt T4wtGT7. (a) Gene diagram overview of T4 wildtype locus spanning *gp52.1* to *rIIB* (top) and edited "GT7" locus (bottom). All genes fully deleted or disrupted are highlighted in dark blue. Homology arms are outlined in magenta. Approximate locations of counterselection protospacers are shown in orange. Primer binding sites for PCR verification are shown in black. Gene coordinates that change between wildtype and edited phage are marked with an asterisk. (b) Growth curves for the homologous recombination editing step for T4wtGT7. (c) Growth curves for the Cas13a counterselection step for T4wtGT7. (d) Plaquing assay for HR lysates against *E. coli* expressing LbuCas13a and crRNA targeting *denB* (left), *ndd* (mid-left), *mcp* (mid-right), or RFP (right). (e) Plaquing assay for HR+E lysates against *E. coli* expressing LbuCas13a and crRNA targeting *denB* (left), *ndd* (mid-left), *mcp* (mid-right), or RFP (right). For all liquid culture assays, induction was performed with 10 nM aTc. For all plate-based assays shown using Cas13a, induction was performed with 5 nM aTc. Labels 1-3 correspond to unique parallel editing workflows.

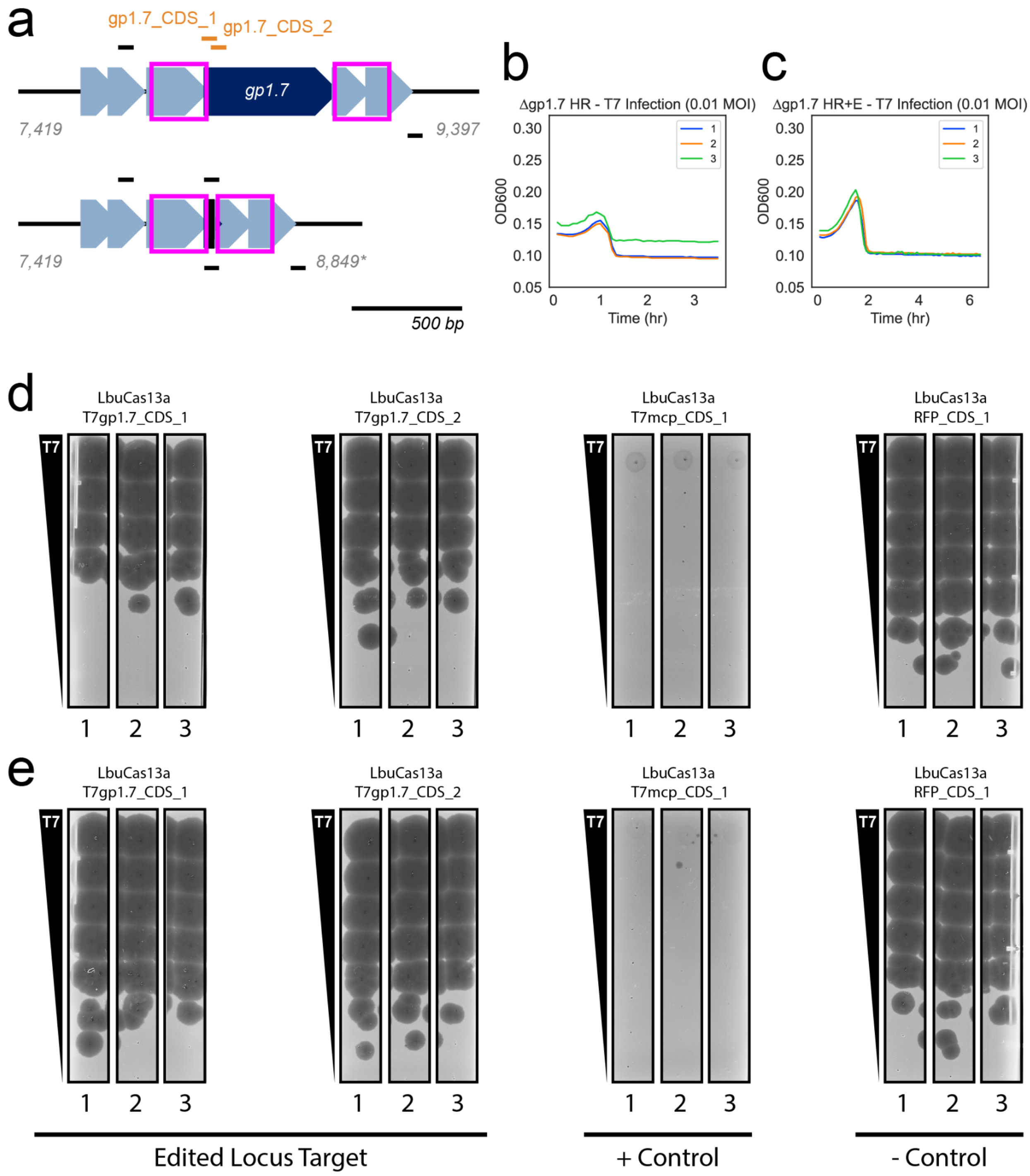

Supplementary Fig. 22. Overview of phenotypic results for editing attempt T7Δ*gp1.7*. (a) Gene diagram overview of T7*gp1.7* wildtype locus (top) and edited locus (bottom). *gp1.7* gene is highlighted in dark blue. Homology arms are outlined in magenta. Approximate locations of counterselection protospacers are shown in orange. Primer binding sites for PCR verification

are shown in black. Gene coordinates that change between wildtype and edited phage are marked with an asterisk. (b) Growth curves for the homologous recombination editing step for T7Δ*gp1.7*. (c) Growth curves for the Cas13a counterselection step for T7*gp1.7*. (d) Plaquing assay for HR lysates against *E. coli* expressing LbuCas13a and crRNA targeting *gp1.7* (left, mid-left), *mcp* (mid-right), or RFP (right). (e) Plaquing assay for HR+E lysates against *E. coli* expressing LbuCas13a and crRNA targeting *gp1.7* (left, mid-left), *mcp* (mid-right), or RFP (right). For all liquid culture assays, induction was performed with 10 nM aTc. For all plate-based assays shown using Cas13a, induction was performed with 5 nM aTc. Labels 1-3 correspond to unique parallel editing workflows.

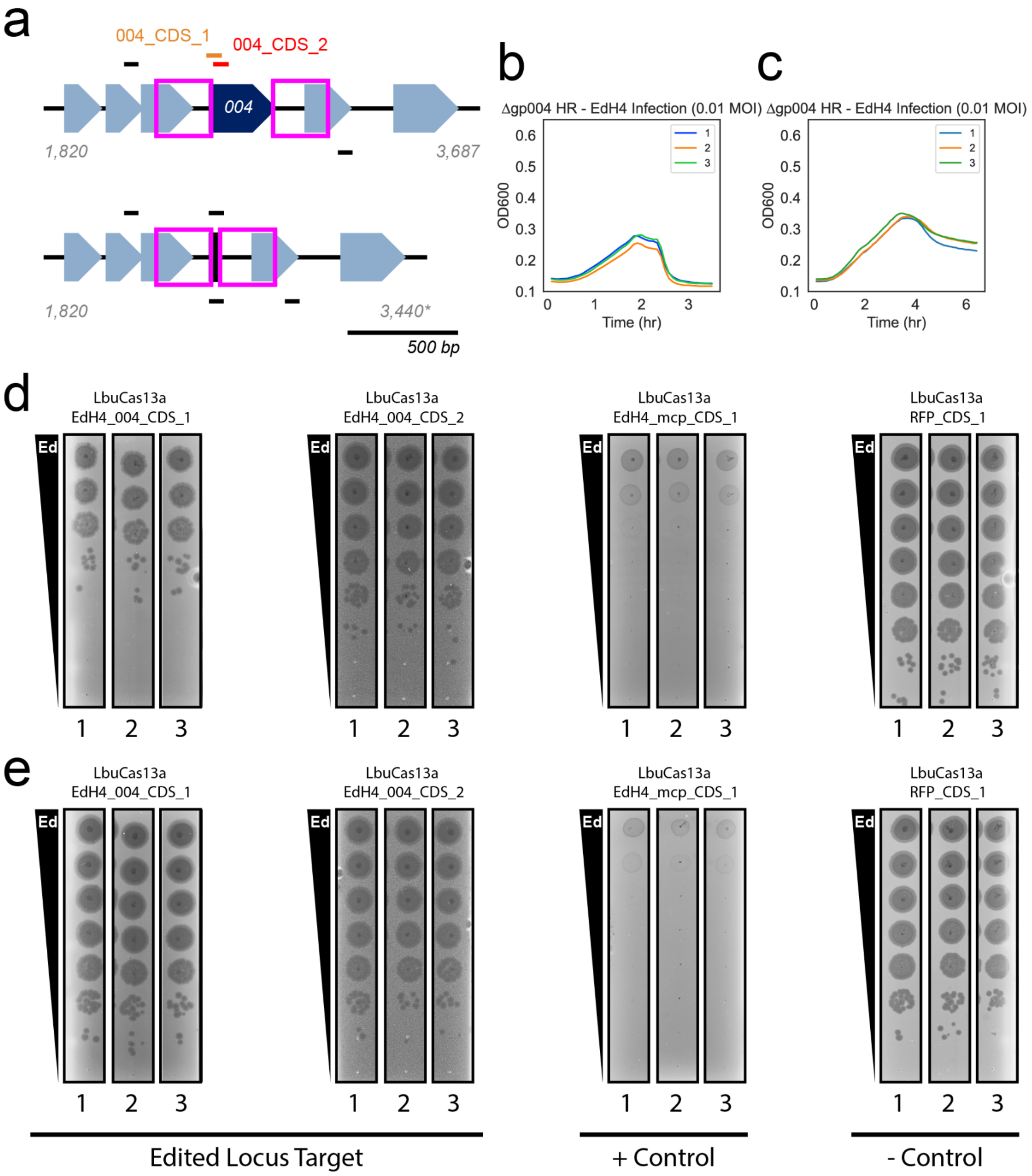

Supplementary Fig. 23. Overview of phenotypic results for editing attempt EdH4Δ*gp004*. (a) Gene diagram overview of EdH4Δ*gp004* wildtype locus (top) and edited locus (bottom). *gp004* gene is highlighted in dark blue. Homology arms are outlined in magenta. Approximate locations of counterselection protospacers are shown in orange. Auto-toxic spacer EdH4_gp004_CDS_2

is highlighted in red. Primer binding sites for PCR verification are shown in black. Gene coordinates that change between wildtype and edited phage are marked with an asterisk. (b) Growth curves for the homologous recombination editing step for EdH4Δ*gp004*. (c) Growth curves for the Cas13a counterselection step for EdH4Δ*gp004*. (d) Plaquing assay for HR lysates against *E. coli* expressing LbuCas13a and crRNA targeting *gp004* (left, mid-left), *mcp* (mid-right), or RFP (right). (e) Plaquing assay for HR+E lysates against *E. coli* expressing LbuCas13a and crRNA targeting *gp004* (left, mid-left), *mcp* (mid-right), or RFP (right). For all liquid culture assays, induction was performed with 10 nM aTc. For all plate-based assays shown using Cas13a, induction was performed with 5 nM aTc. Labels 1-3 correspond to unique parallel editing workflows.

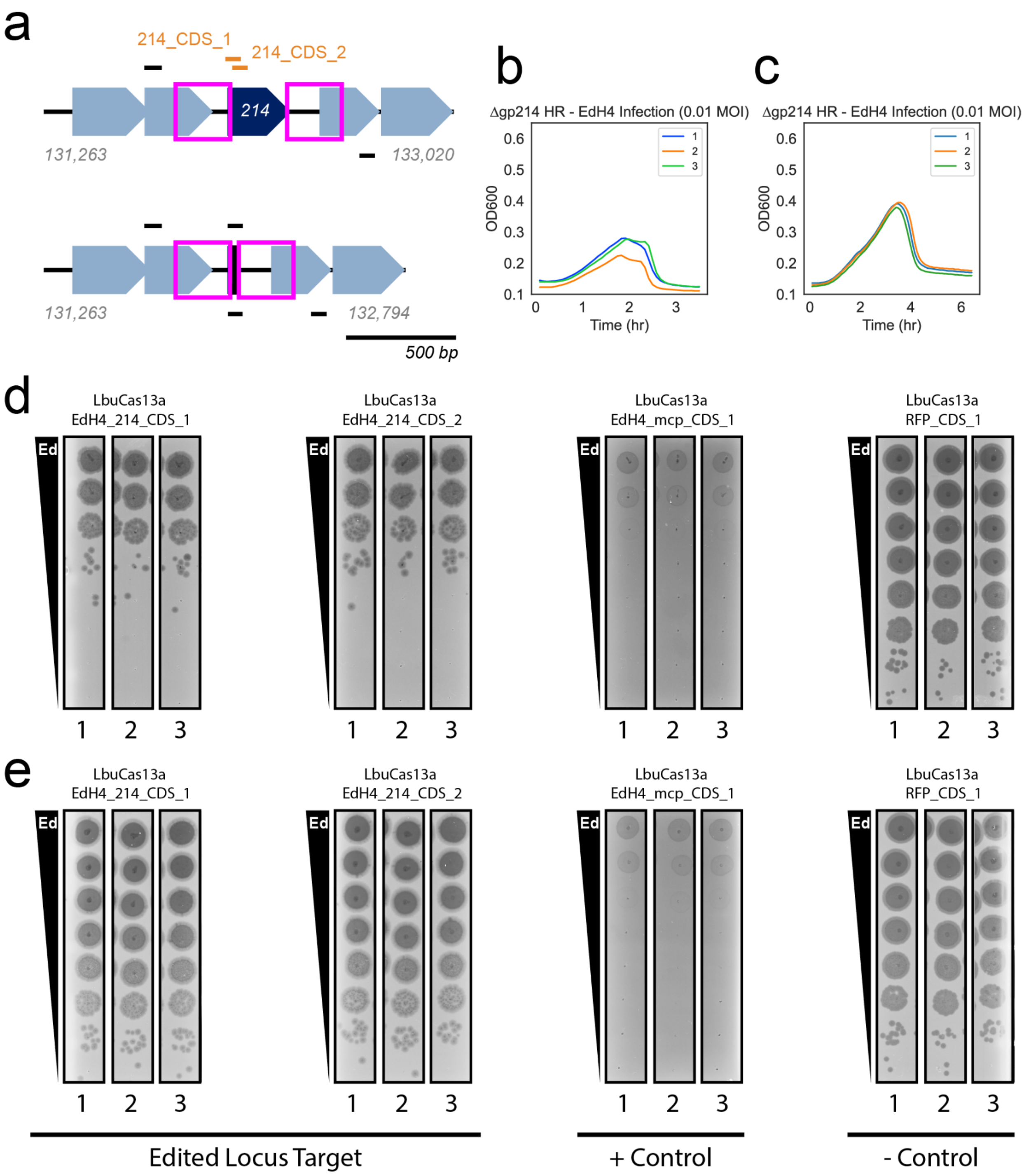

Supplementary Fig. 24. Overview of phenotypic results for editing attempt EdH4Δ*gp214*. (a) Gene diagram overview of EdH4Δ*gp214* wildtype locus (top) and edited locus (bottom). *gp0214* gene is highlighted in dark blue. Homology arms are outlined in magenta. Approximate locations of counterselection protospacers are shown in orange. Primer binding sites for PCR verification

are shown in black. Gene coordinates that change between wildtype and edited phage are marked with an asterisk. (b) Growth curves for the homologous recombination editing step for EdH4Δ*gp0214*. (c) Growth curves for the Cas13a counterselection step for EdH4Δ*gp004*. (d) Plaquing assay for HR lysates against *E. coli* expressing LbuCas13a and crRNA targeting *gp0214* (left, mid-left), *mcp* (mid-right), or RFP (right). (e) Plaquing assay for HR+E lysates against *E. coli* expressing LbuCas13a and crRNA targeting *gp0214* (left, mid-left), *mcp* (mid-right), or RFP (right). For all liquid culture assays, induction was performed with 10 nM aTc. For all plate-based assays shown using Cas13a, induction was performed with 5 nM aTc. Labels 1-3 correspond to unique parallel editing workflows.

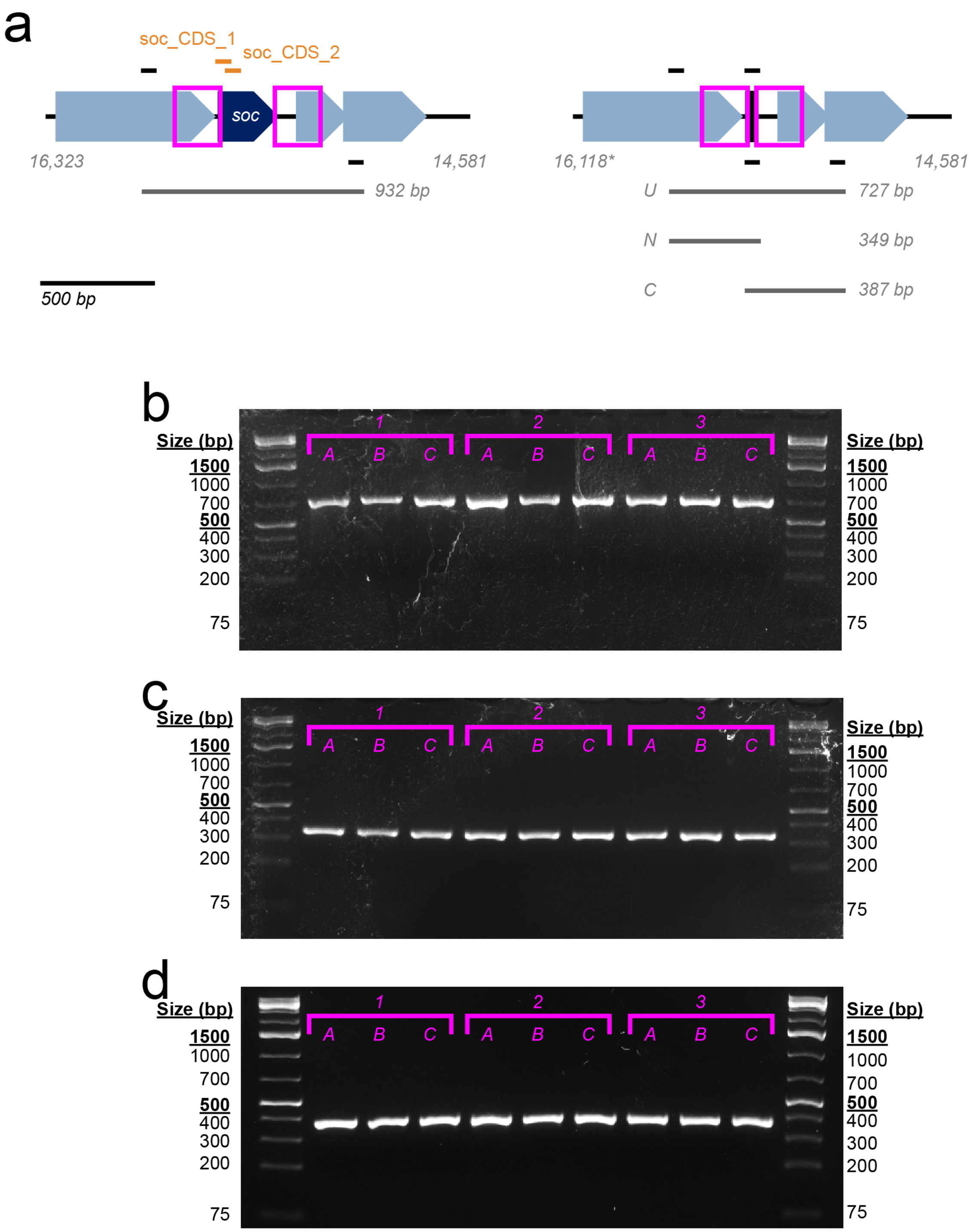

Supplementary Fig. 25. Overview of genotyping results for editing attempt T4Δ*soc*. (a) Gene diagram overview of T4*soc* wildtype locus (left) and edited locus (right). *soc* gene is highlighted in dark blue. Homology arms are outlined in magenta. Approximate locations of counterselection protospacers are shown in orange. Primer binding sites for PCR verification are shown in black.

Gene coordinates that change between wildtype and edited phage are marked with an asterisk. PCR product lengths for unbiased (U), N-terminal (N), and C-terminal (C) PCRs are shown in grey. (b, c, d) PCR products shown for (b) unbiased, (c) N-terminal, and (d) C-terminal PCRs. PCR results are produced from three individual plaques (A, B, C) from selective plaquing at the end of three independent editing processes (1, 2, 3).

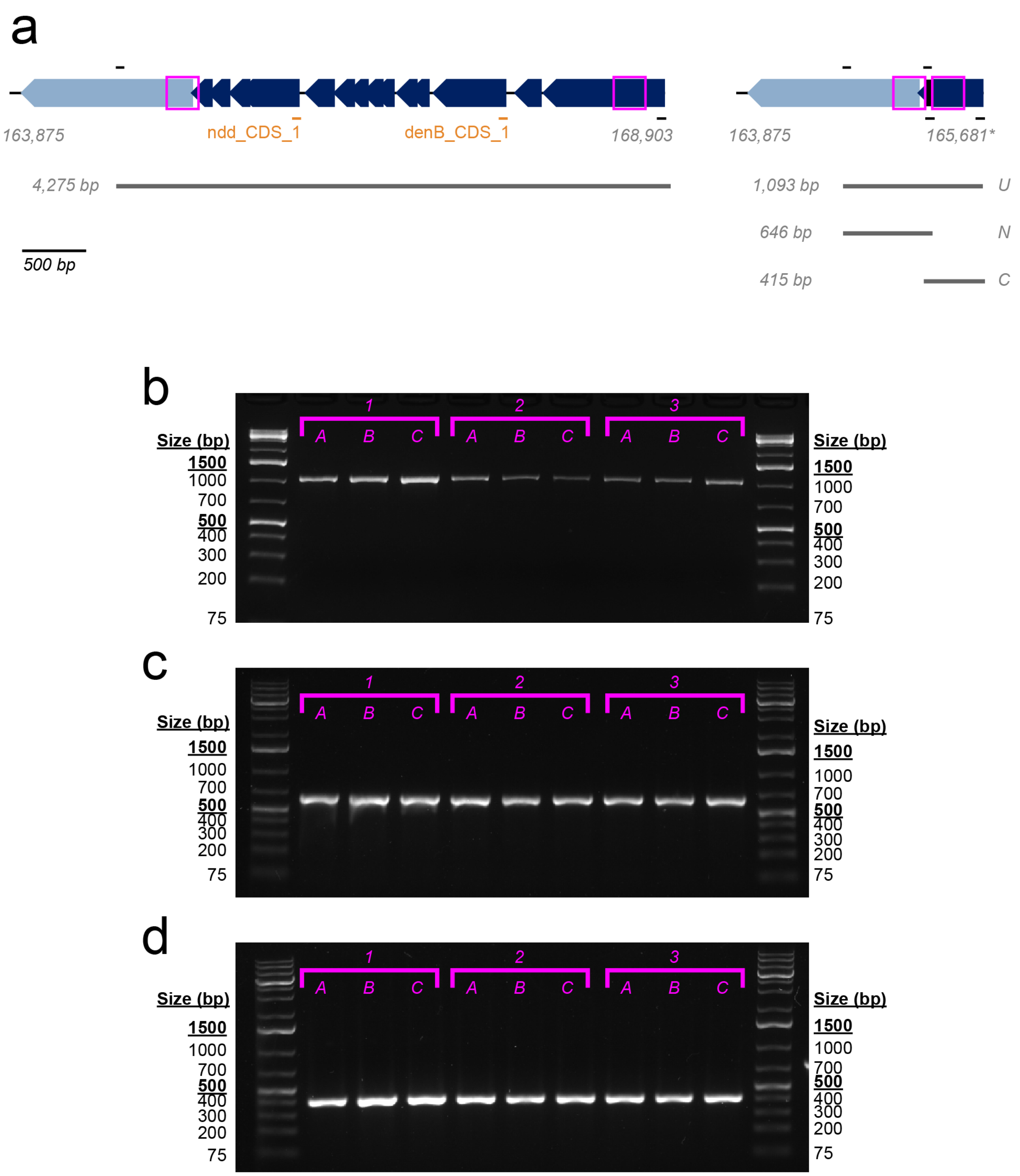

Supplementary Fig. 26. Overview of genotyping results for editing attempt T4wtGT7. (a) Gene diagram overview of T4wtGT7 wildtype locus (left) and edited locus (right). All genes fully

deleted or disrupted are highlighted in dark blue. Homology arms are outlined in magenta. Approximate locations of counterselection protospacers are shown in orange. Primer binding sites for PCR verification are shown in black. Gene coordinates that change between wildtype and edited phage are marked with an asterisk. PCR product lengths for unbiased (U), N-terminal (N), and C-terminal (C) PCRs are shown in grey. (b, c, d) PCR products shown for (b) unbiased, (c) N-terminal, and (d) C-terminal PCRs. PCR results are produced from three individual plaques (A, B, C) from selective plaquing at the end of three independent editing processes (1, 2, 3).

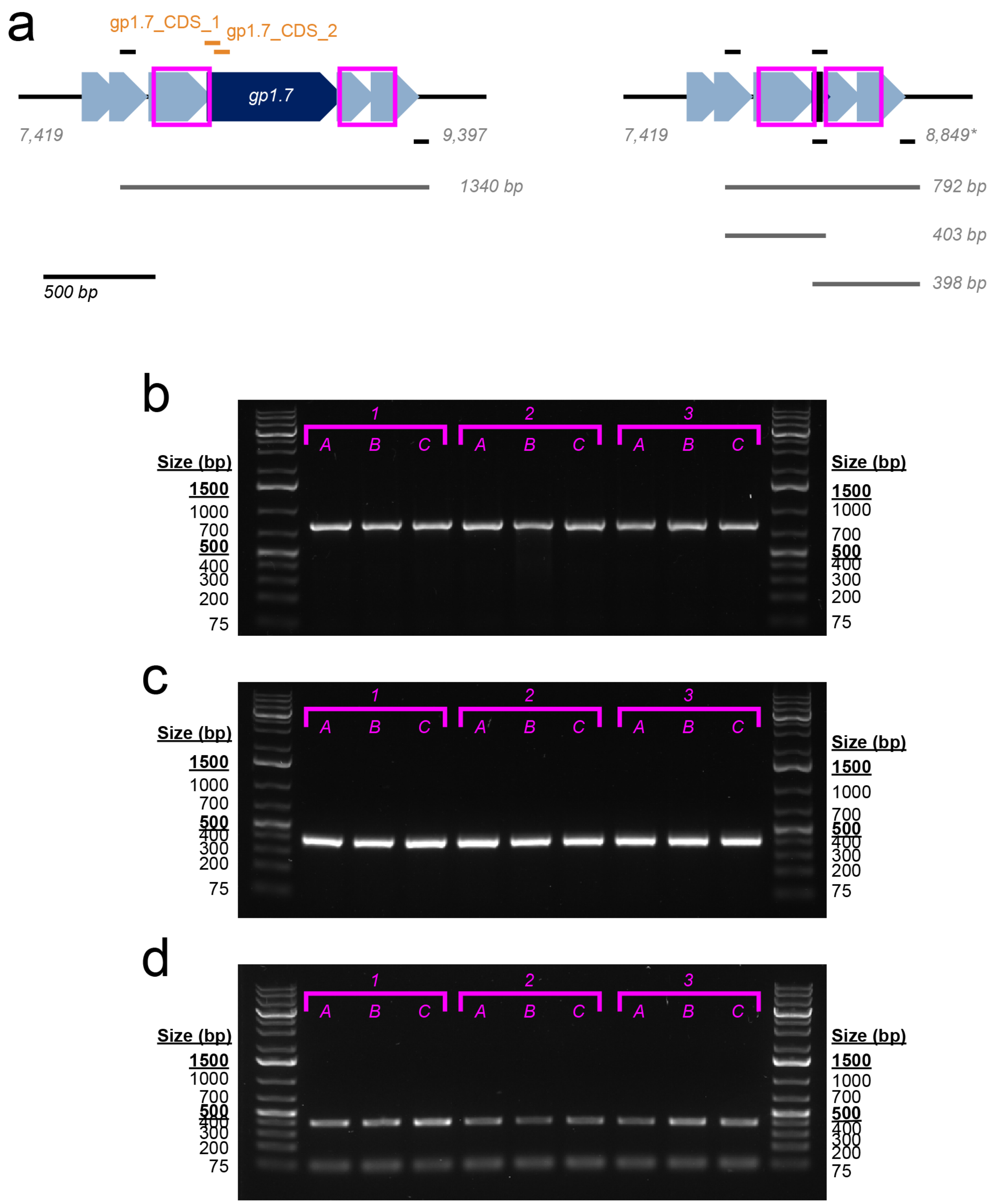

Supplementary Fig. 27. Overview of genotyping results for editing attempt T7Δ*gp1.7*. (a) Gene diagram overview of T7*gp1.7* wildtype locus (left) and edited locus (right). All genes fully deleted or disrupted are highlighted in dark blue. Homology arms are outlined in magenta. Approximate locations of counterselection protospacers are shown in orange. Primer binding sites for PCR

verification are shown in black. Gene coordinates that change between wildtype and edited phage are marked with an asterisk. PCR product lengths for unbiased (U), N-terminal (N), and C-terminal (C) PCRs are shown in grey. (b, c, d) PCR products shown for (b) unbiased, (c) N-terminal, and (d) C-terminal PCRs. PCR results are produced from three individual plaques (A, B, C) from selective plaquing at the end of three independent editing processes (1, 2, 3). For T7 experiments, plaques for genotyping were picked before they fully matured to avoid picking multiple plaques.

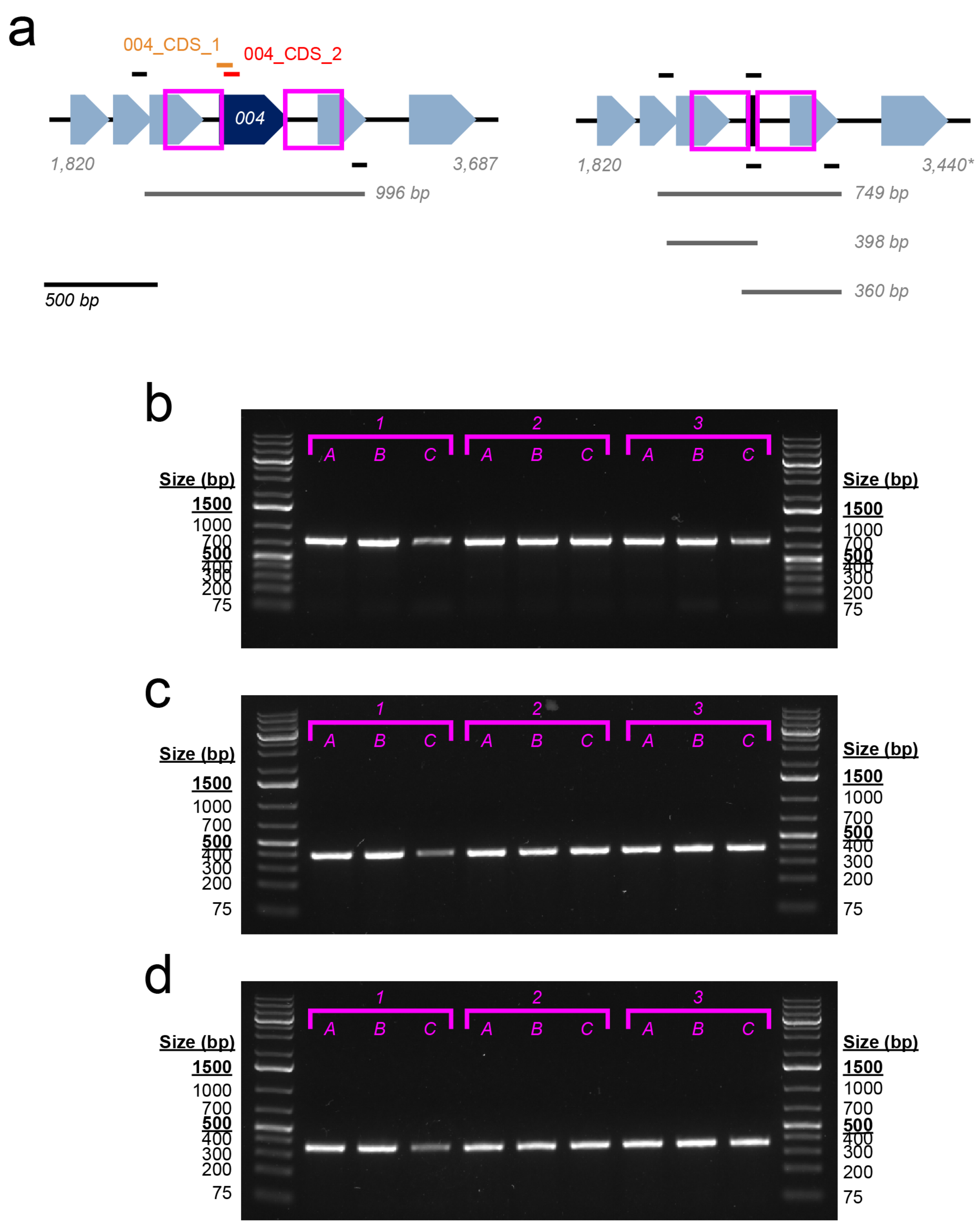

Supplementary Fig. 28. Overview of genotyping results for editing attempt EdH4Δ*gp004*. (a) Gene diagram overview of EdH4*gp004* wildtype locus (left) and edited locus (right). All genes fully deleted or disrupted are highlighted in dark blue. Homology arms are outlined in magenta. Approximate locations of counterselection protospacers are shown in orange. Primer binding

sites for PCR verification are shown in black. Gene coordinates that change between wildtype and edited phage are marked with an asterisk. PCR product lengths for unbiased (U), N-terminal (N), and C-terminal (C) PCRs are shown in grey. (b, c, d) PCR products shown for (b) unbiased, (c) N-terminal, and (d) C-terminal PCRs. PCR results are produced from three individual plaques (A, B, C) from selective plaquing at the end of three independent editing processes (1, 2, 3).

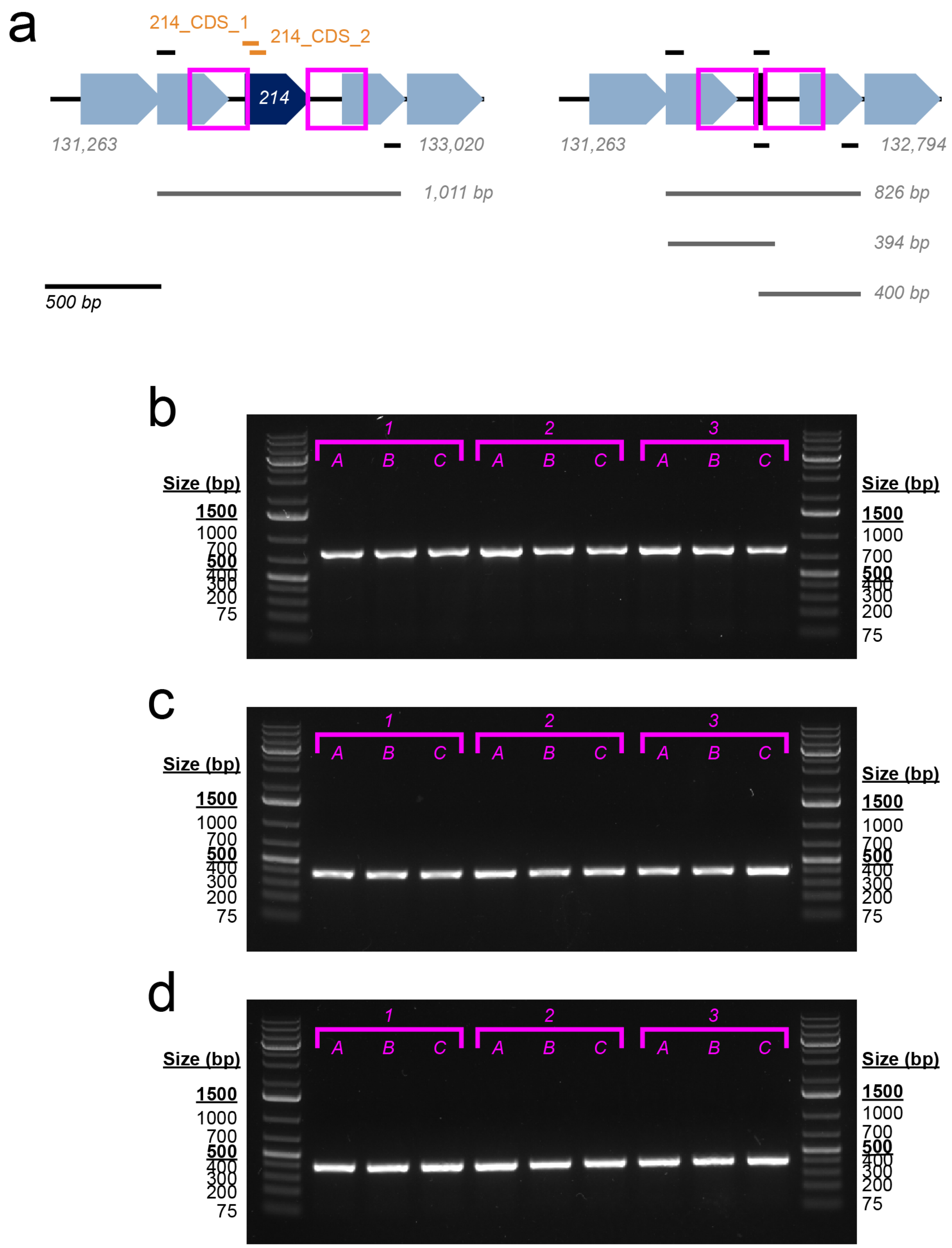

Supplementary Fig. 29. Overview of genotyping results for editing attempt EdH4Δ*gp214*. (a) Gene diagram overview of EdH4*gp214* wildtype locus (left) and edited locus (right). All genes fully deleted or disrupted are highlighted in dark blue. Homology arms are outlined in magenta. Approximate locations of counterselection protospacers are shown in orange. Primer binding

sites for PCR verification are shown in black. Gene coordinates that change between wildtype and edited phage are marked with an asterisk. PCR product lengths for unbiased (U), N-terminal (N), and C-terminal (C) PCRs are shown in grey. (b, c, d) PCR products shown for (b) unbiased, (c) N-terminal, and (d) C-terminal PCRs. PCR results are produced from three individual plaques (A, B, C) from selective plaquing at the end of three independent editing processes (1, 2, 3).

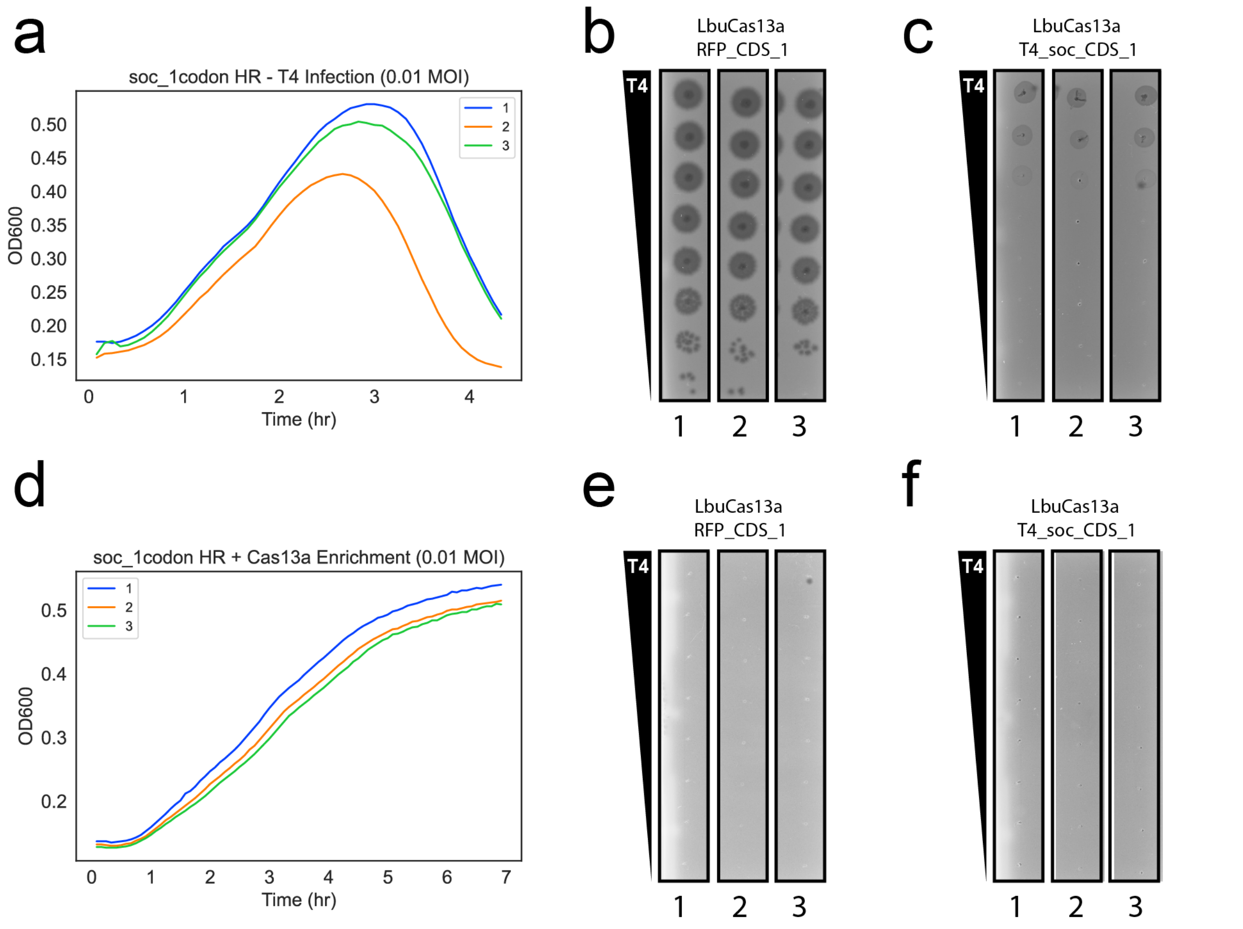

Supplementary Fig. 30. Overview of phenotypic results for editing attempt *soc*-C. (a) Growth curves for the homologous recombination editing step for *soc*-C. (b) Non-selective plaquing assay for lysates from (a) on a non-targeting crRNA. (c) Selective plaquing assay for lysates from (a) using the corresponding *soc*-targeting crRNA. (d) Growth curves for the enrichment step for lysates harboring, but not enriched for the *soc*-C edit. (e) Non-selective plaquing assay for lysates from (d) on a non-targeting crRNA. (f) Selective plaquing assay for lysates from (d) using the corresponding *soc*-targeting crRNA. For all liquid culture and plate-based assays shown using Cas13a, induction was performed with 10 nM aTc. Labels 1-3 correspond to unique parallel editing workflows.

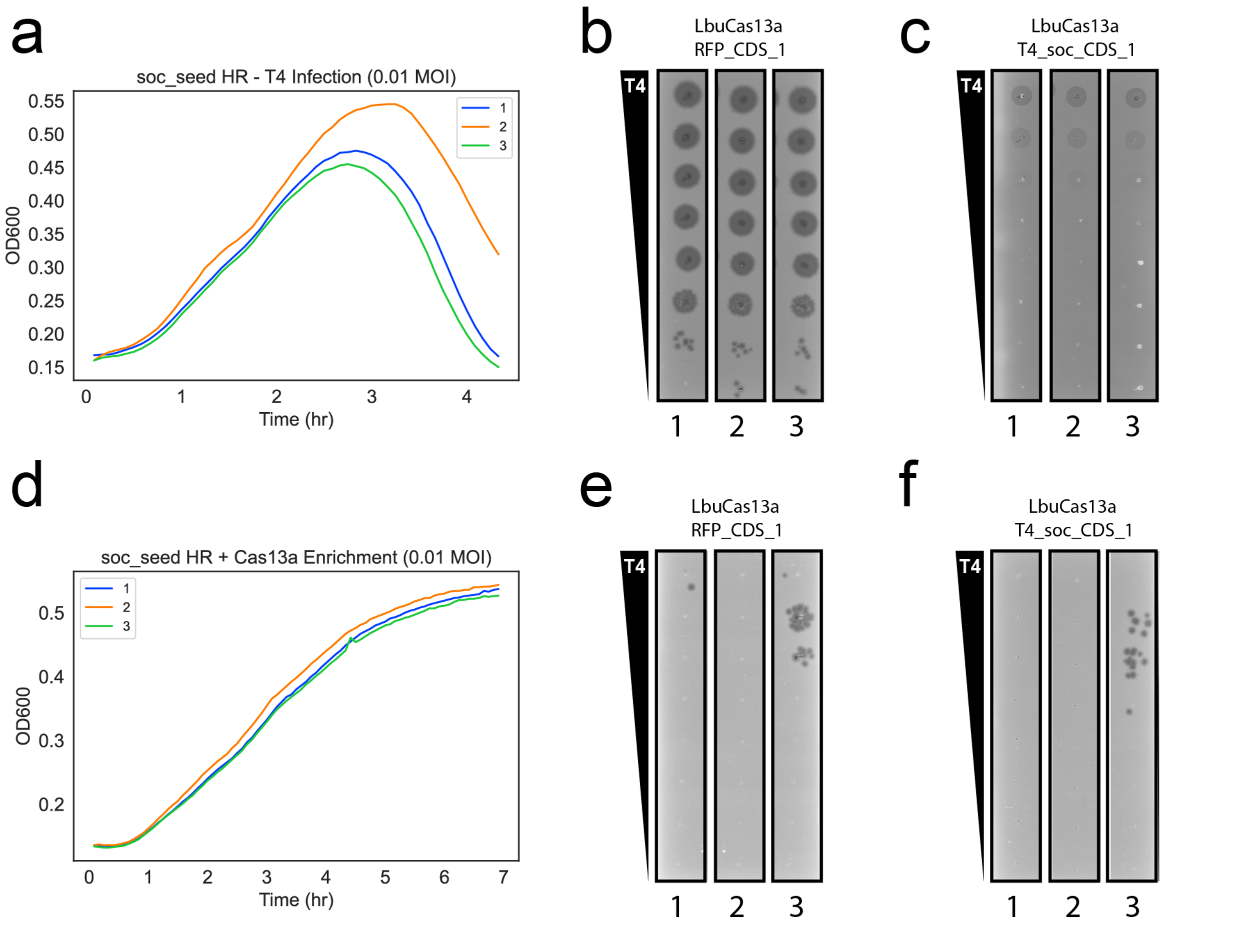

Supplementary Fig. 31. Overview of phenotypic results for editing attempt *soc*-S. (a) Growth curves for the homologous recombination editing step for *soc*-S. (b) Non-selective plaquing assay for lysates from (a) on a non-targeting crRNA. (c) Selective plaquing assay for lysates from (a) using the corresponding *soc*-targeting crRNA. (d) Growth curves for the enrichment step for lysates harboring, but not enriched for the *soc*-S edit. (e) Non-selective plaquing assay for lysates from (d) on a non-targeting crRNA. (f) Selective plaquing assay for lysates from (d) using the corresponding *soc*-targeting crRNA. For all liquid culture and plate-based assays shown using Cas13a, induction was performed with 10 nM aTc. Labels 1-3 correspond to unique parallel editing workflows.

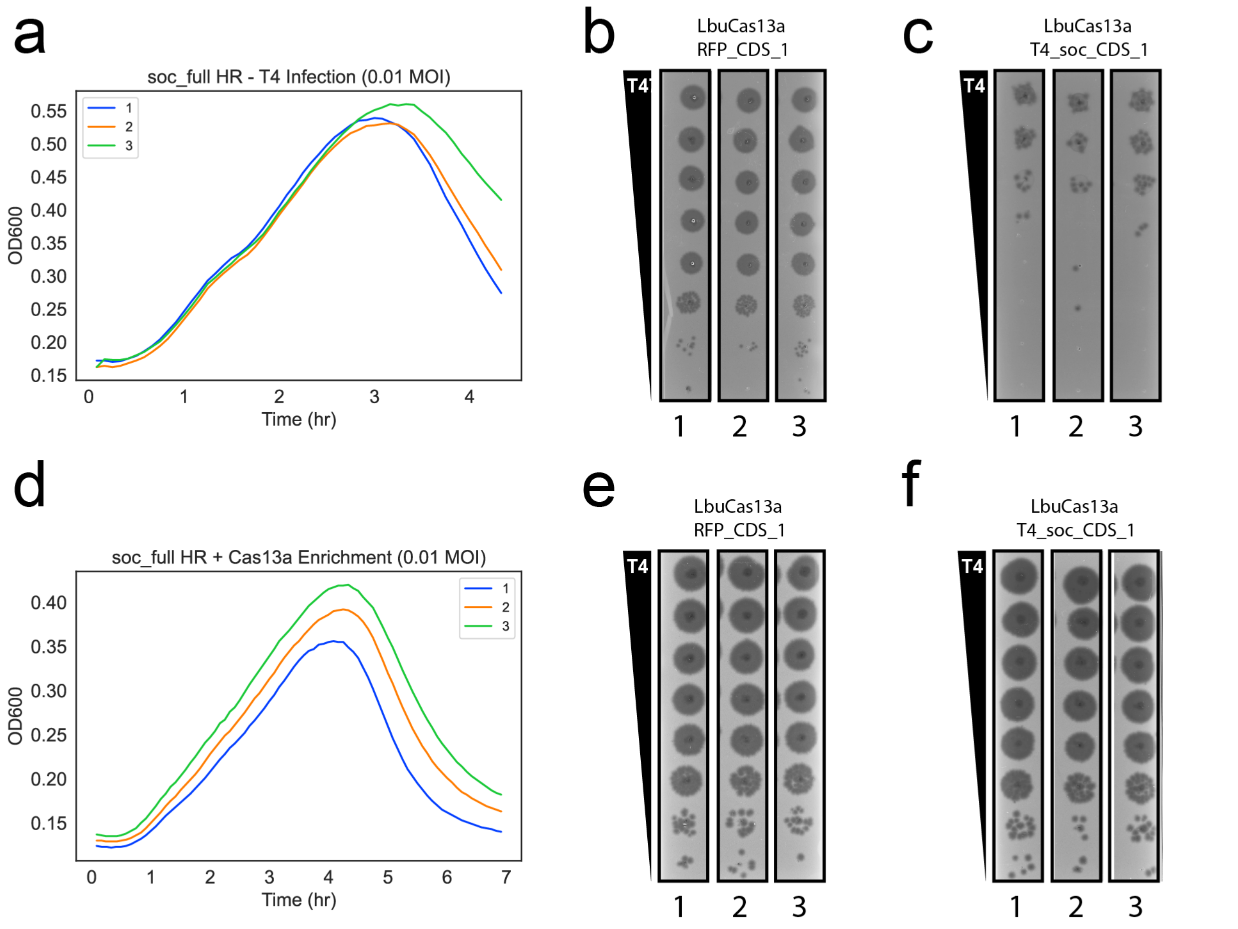

Supplementary Fig. 32. Overview of phenotypic results for editing attempt *soc*-F. (a) Growth curves for the homologous recombination editing step for *soc*-F. (b) Non-selective plaquing assay for lysates from (a) on a non-targeting crRNA. (c) Selective plaquing assay for lysates from (a) using the corresponding *soc*-targeting crRNA. (d) Growth curves for the enrichment step for lysates harboring, but not enriched for the *soc*-F edit. (e) Non-selective plaquing assay for lysates from (d) on a non-targeting crRNA. (f) Selective plaquing assay for lysates from (d) using the corresponding *soc*-targeting crRNA. For all liquid culture and plate-based assays shown using Cas13a, induction was performed with 10 nM aTc. Labels 1-3 correspond to unique parallel editing workflows.

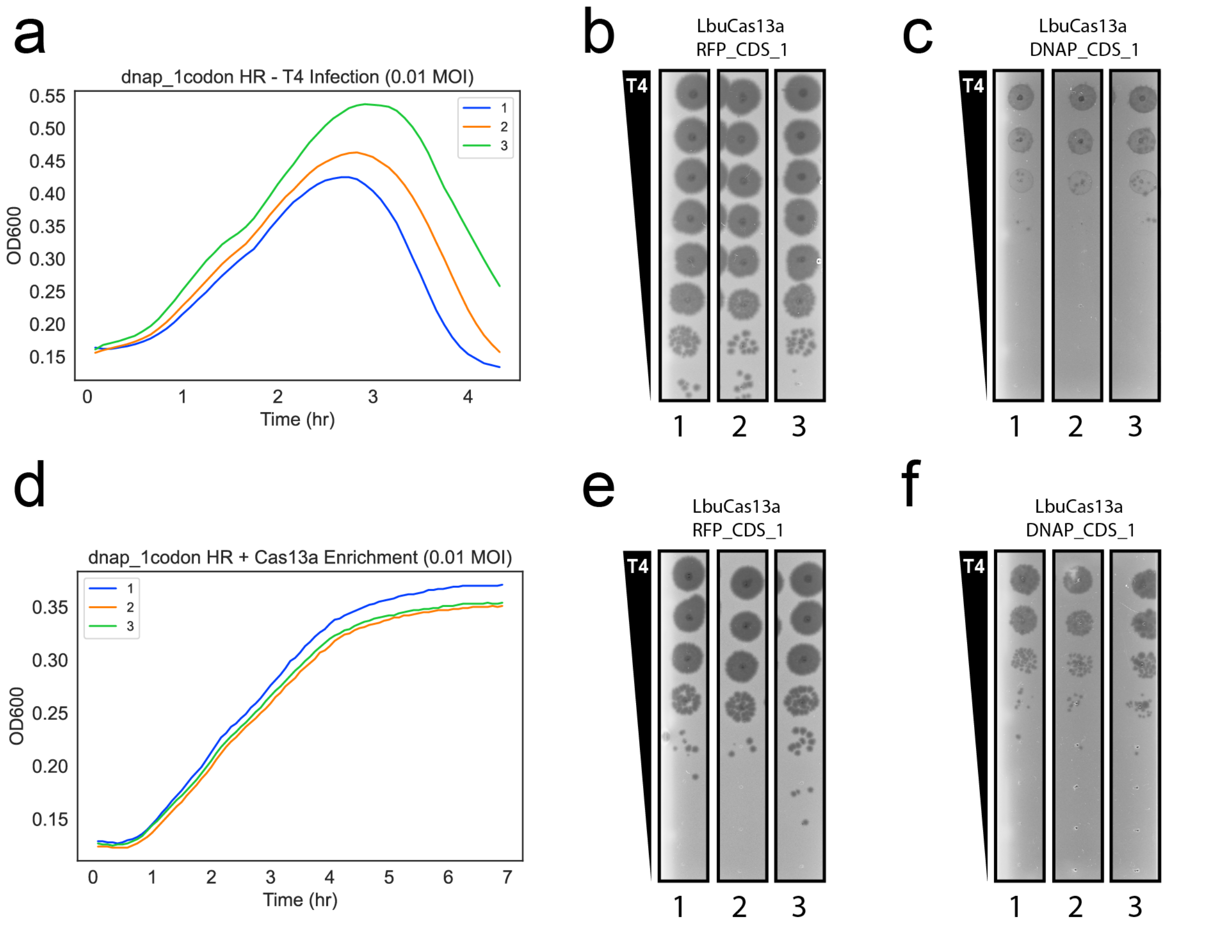

Supplementary Fig. 33. Overview of phenotypic results for editing attempt *dnap*-C. (a) Growth curves for the homologous recombination editing step for *dnap*-C. (b) Non-selective plaquing assay for lysates from (a) on a non-targeting crRNA. (c) Selective plaquing assay for lysates from (a) using the corresponding *dnap*-targeting crRNA. (d) Growth curves for the enrichment step for lysates harboring, but not enriched for the *dnap*-C edit. (e) Non-selective plaquing assay for lysates from (d) on a non-targeting crRNA. (f) Selective plaquing assay for lysates from (d) using the corresponding *dnap*-targeting crRNA. For all liquid culture assays shown using Cas13a, induction was performed with 10 nM aTc. For all plaquing assays shown using Cas13a, induction was performed with 5 nM aTc. Labels 1-3 correspond to unique parallel editing workflows.

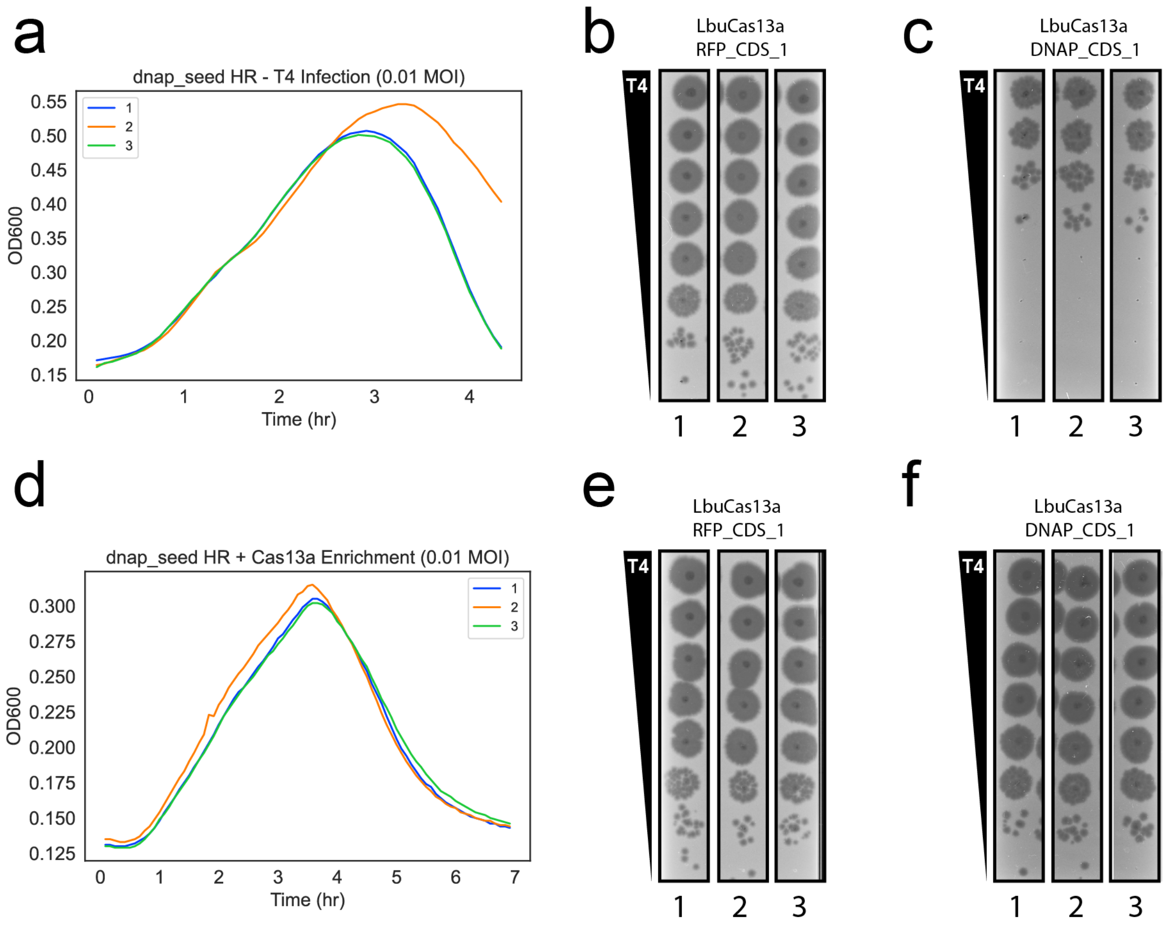

Supplementary Fig. 34. Overview of phenotypic results for editing attempt *dnap*-S. (a) Growth curves for the homologous recombination editing step for *dnap*-S. (b) Non-selective plaquing assay for lysates from (a) on a non-targeting crRNA. (c) Selective plaquing assay for lysates from (a) using the corresponding *dnap*-targeting crRNA. (d) Growth curves for the enrichment step for lysates harboring, but not enriched for the *dnap*-S edit. (e) Non-selective plaquing assay for lysates from (d) on a non-targeting crRNA. (f) Selective plaquing assay for lysates from (d) using the corresponding *dnap*-targeting crRNA. For all liquid culture assays shown using Cas13a, induction was performed with 10 nM aTc. For all plaquing assays shown using Cas13a, induction was performed with 5 nM aTc. Labels 1-3 correspond to unique parallel editing workflows.

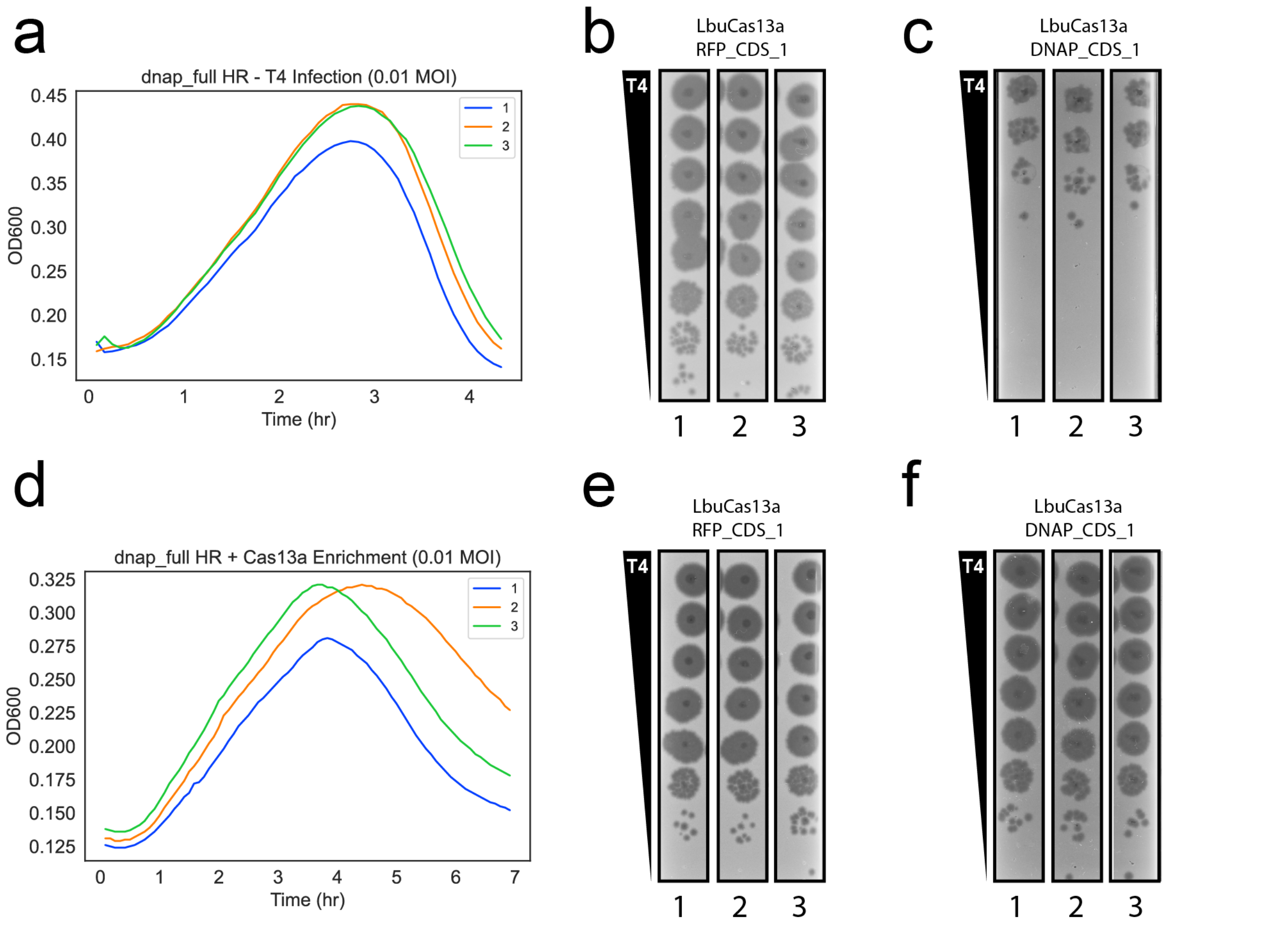

Supplementary Fig. 35. Overview of phenotypic results for editing attempt *dnap*-F. (a) Growth curves for the homologous recombination editing step for *dnap*-F. (b) Non-selective plaquing assay for lysates from (a) on a non-targeting crRNA. (c) Selective plaquing assay for lysates from (a) using the corresponding *dnap*-targeting crRNA. (d) Growth curves for the enrichment step for lysates harboring, but not enriched for the *dnap*-F edit. (e) Non-selective plaquing assay for lysates from (d) on a non-targeting crRNA. (f) Selective plaquing assay for lysates from (d) using the corresponding *dnap*-targeting crRNA. For all liquid culture assays shown using Cas13a, induction was performed with 10 nM aTc. For all plaquing assays shown using Cas13a, induction was performed with 5 nM aTc. Labels 1-3 correspond to unique parallel editing workflows.

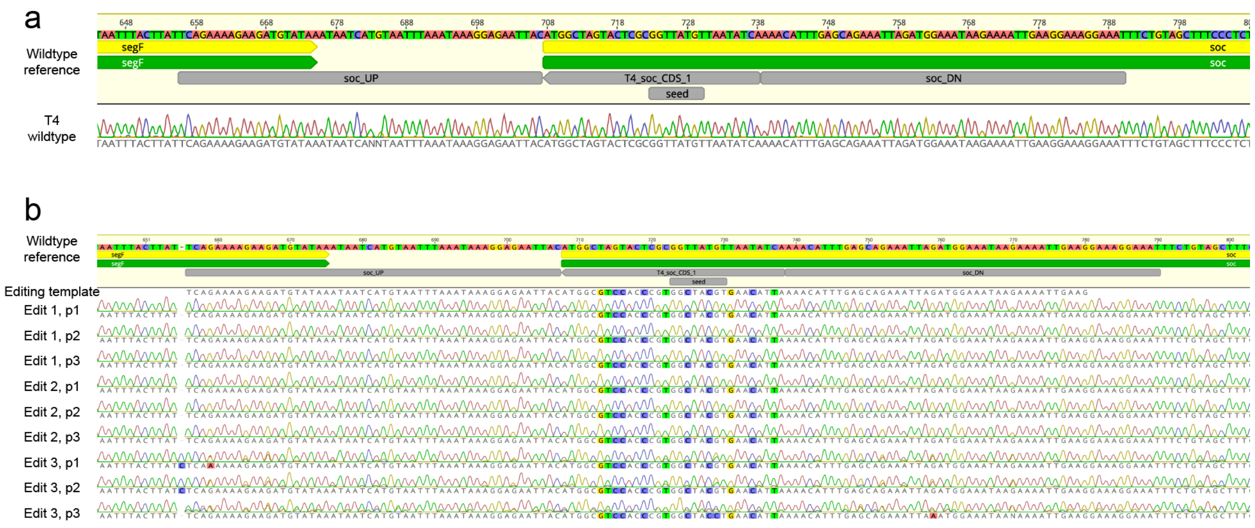

Supplementary Fig. 36. Overview of genotyping results for *soc* editing attempts. (a) Sanger sequencing trace from a PCR at the wildtype T4 *soc* locus. (b) Sanger sequencing trace from unbiased PCRs on individual plaques at the *soc* locus for *soc-F* edits. Results for three individual plaques (p1, p2, p3) are shown for enriched, selective plaquing from three independent editing replicates (“Edit 1”, “Edit 2”, “Edit 3”) (for instance 3 plaques from each of the dilutions shown in Supplementary Figure 32f). The wildtype T4*soc* locus and the editing template is shown as a reference. Mutations relative to the wildtype locus are highlighted.

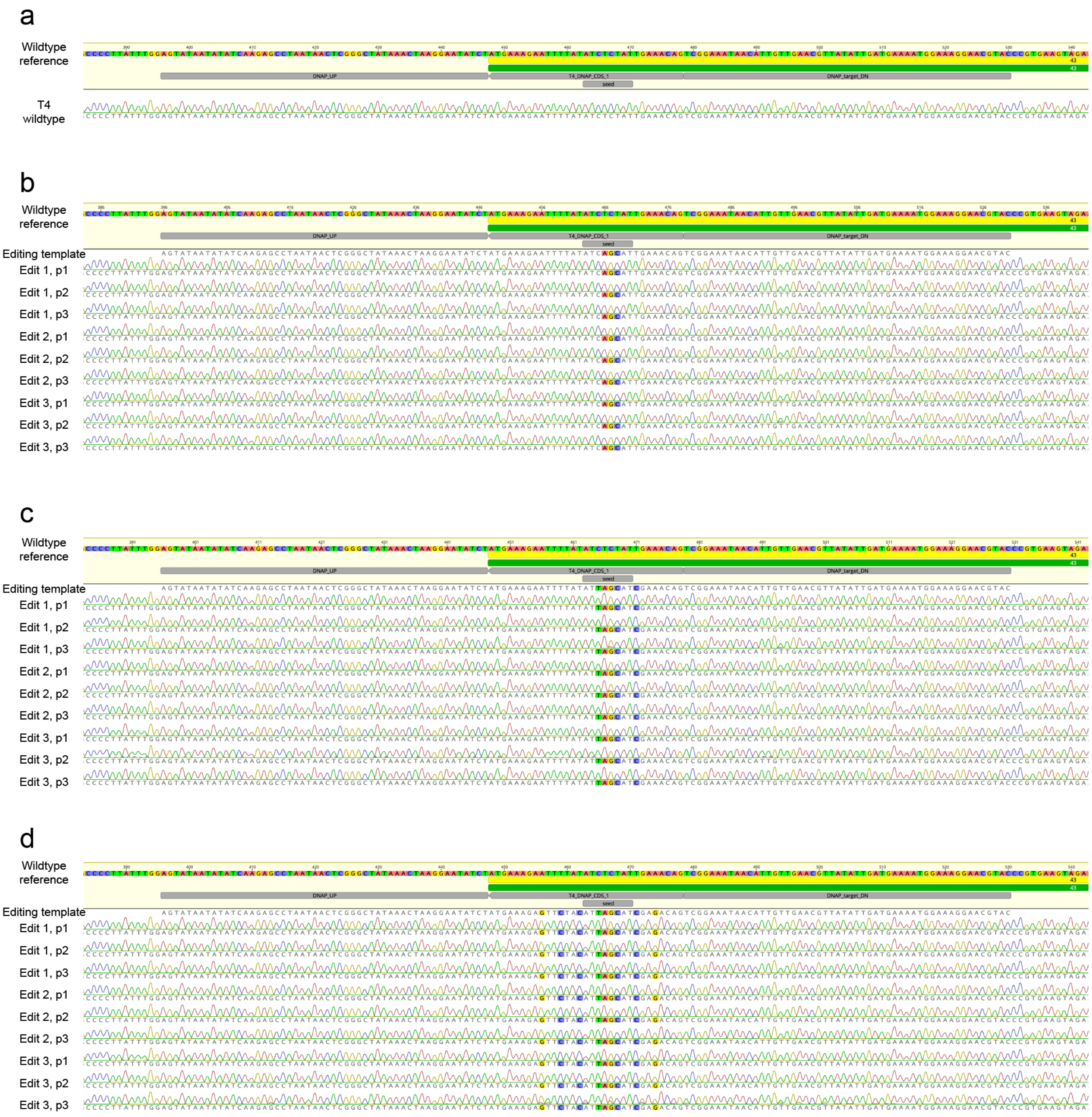

Supplementary Fig. 37. Overview of genotyping results for *dnap* editing attempts. (a) Sanger sequencing trace from a PCR at the wildtype T4 *dnap* locus. (b-d) Sanger sequencing trace from unbiased PCRs on individual plaques at the *dnap* locus for (b) *dnap*-C, (c) *dnap*-S, and (d) *dnap*-F edits. Results for three individual plaques (p1, p2, p3) are shown for enriched, selective plaquing from three independent editing replicates (“Edit 1”, “Edit 2”, “Edit 3”) (for instance 3 plaques from each of the dilutions shown in Supplementary Figure 33f, 34f, and 35f for *dnap*-C,

dnap-S, and *dnap*-F respectively). The wildtype T4*dnap* locus and the editing template is shown as a reference. Mutations relative to the wildtype locus are highlighted.
