# Supplementary material for: Broad-spectrum CRISPR-Cas13a enables efficient phage genome editing
Source: Nat Microbiol. 2022 Oct 31;7(12):1967–79. doi: 10.1038/s41564-022-01258-x (PMC9712115; doi:10.1038/s41564-022-01258-x)
Supplement: Supplementary file 2 — Reporting Summary [file 41564_2022_1258_MOESM2_ESM.pdf]

nature portfolio

Corresponding author(s): Jennifer Doudna

Last updated by author(s): Aug 4, 2022

Reporting Summary

Nature Portfolio wishes to improve the reproducibility of the work that we publish. This form provides structure for consistency and transparency in reporting. For further information on Nature Portfolio policies, see our Editorial Policies and the Editorial Policy Checklist.

Statistics

For all statistical analyses, confirm that the following items are present in the figure legend, table legend, main text, or Methods section.

n/a	Confirmed	
☐	☒	The exact sample size (n) for each experimental group/condition, given as a discrete number and unit of measurement
☐	☒	A statement on whether measurements were taken from distinct samples or whether the same sample was measured repeatedly
☒	☐	The statistical test(s) used AND whether they are one- or two-sided<br>*Only common tests should be described solely by name; describe more complex techniques in the Methods section.*
☐	☒	A description of all covariates tested
☐	☒	A description of any assumptions or corrections, such as tests of normality and adjustment for multiple comparisons
☐	☒	A full description of the statistical parameters including central tendency (e.g. means) or other basic estimates (e.g. regression coefficient) AND variation (e.g. standard deviation) or associated estimates of uncertainty (e.g. confidence intervals)
☒	☐	For null hypothesis testing, the test statistic (e.g. F, t, r) with confidence intervals, effect sizes, degrees of freedom and P value noted<br>*Give P values as exact values whenever suitable.*
☒	☐	For Bayesian analysis, information on the choice of priors and Markov chain Monte Carlo settings
☒	☐	For hierarchical and complex designs, identification of the appropriate level for tests and full reporting of outcomes
☒	☐	Estimates of effect sizes (e.g. Cohen's d, Pearson's r), indicating how they were calculated

Our web collection on statistics for biologists contains articles on many of the points above.

Software and code

Policy information about availability of computer code

Data collection	No software was used for data collection.
Data analysis	The following software was used: Python 3.7.3, Graphpad Prism 9, vContact2, Geneious2022.2.1, IQ-TREE v1.6.12, CD-HIT v4.8.1, MUSCLE v3.8.31

For manuscripts utilizing custom algorithms or software that are central to the research but not yet described in published literature, software must be made available to editors and reviewers. We strongly encourage code deposition in a community repository (e.g. GitHub). See the Nature Portfolio guidelines for submitting code & software for further information.

Data

Policy information about availability of data

All manuscripts must include a data availability statement. This statement should provide the following information, where applicable:
- Accession codes, unique identifiers, or web links for publicly available datasets
- A description of any restrictions on data availability
- For clinical datasets or third party data, please ensure that the statement adheres to our policy

Cas13 entry, Cas13 negative control, and homologous recombination plasmids are available from Addgene (addgene.org) (Addgene #186235-#186247, 189580, 189582, 189584, 189587, 189589). All phage genome sequences, plasmids, oligonucleotides, gene fragments, and DNA sequences can be found in Supplementary Tables 2-6, respectively.

Field-specific reporting

Please select the one below that is the best fit for your research. If you are not sure, read the appropriate sections before making your selection.

[x] Life sciences [ ] Behavioural & social sciences [ ] Ecological, evolutionary & environmental sciences

For a reference copy of the document with all sections, see nature.com/documents/nr-reporting-summary-flat.pdf

Life sciences study design

All studies must disclose on these points even when the disclosure is negative.

Sample size	We performed experiments in biological triplicate (N=3) to facilitate measurements of both mean and dispersion.<br><br>For phage-genome editing workflows, there is not an established reproducibility framework. We adopted practices used in phage infection reproducibility and performed editing experiments in biological triplicate (N=3).
Data exclusions	For bioinformatic analysis shown in Fig.1, some samples were excluded due to incomplete coding sequence content (ie incomplete genes) or fusions. Redundant Cas13 variants were de-replicated with CD-HIT as described in Methods.<br><br>No experimental samples were excluded in this study.
Replication	All experiments were performed in biological triplicate. All phage genome editing experiments were performed in biological triplicate, followed by sequence confirmation across both biological and technical triplicates. All experiments were successful unless otherwise noted in the manuscript.
Randomization	This study does not involve subjects that require randomization
Blinding	This study does not involve subjects that require blinding

Reporting for specific materials, systems and methods

We require information from authors about some types of materials, experimental systems and methods used in many studies. Here, indicate whether each material, system or method listed is relevant to your study. If you are not sure if a list item applies to your research, read the appropriate section before selecting a response.

Materials & experimental systems

n/a	Involved in the study	
[x]	[ ]	Antibodies
[x]	[ ]	Eukaryotic cell lines
[x]	[ ]	Palaeontology and archaeology
[x]	[ ]	Animals and other organisms
[x]	[ ]	Human research participants
[x]	[ ]	Clinical data
[x]	[ ]	Dual use research of concern

Methods

n/a	Involved in the study	
[x]	[ ]	ChIP-seq
[x]	[ ]	Flow cytometry
[x]	[ ]	MRI-based neuroimaging
