# Supplementary figures and images for: Broad-spectrum CRISPR-Cas13a enables efficient phage genome editing
Source: Nat Microbiol. 2022 Oct 31;7(12):1967–79. doi: 10.1038/s41564-022-01258-x (PMC9712115; doi:10.1038/s41564-022-01258-x)

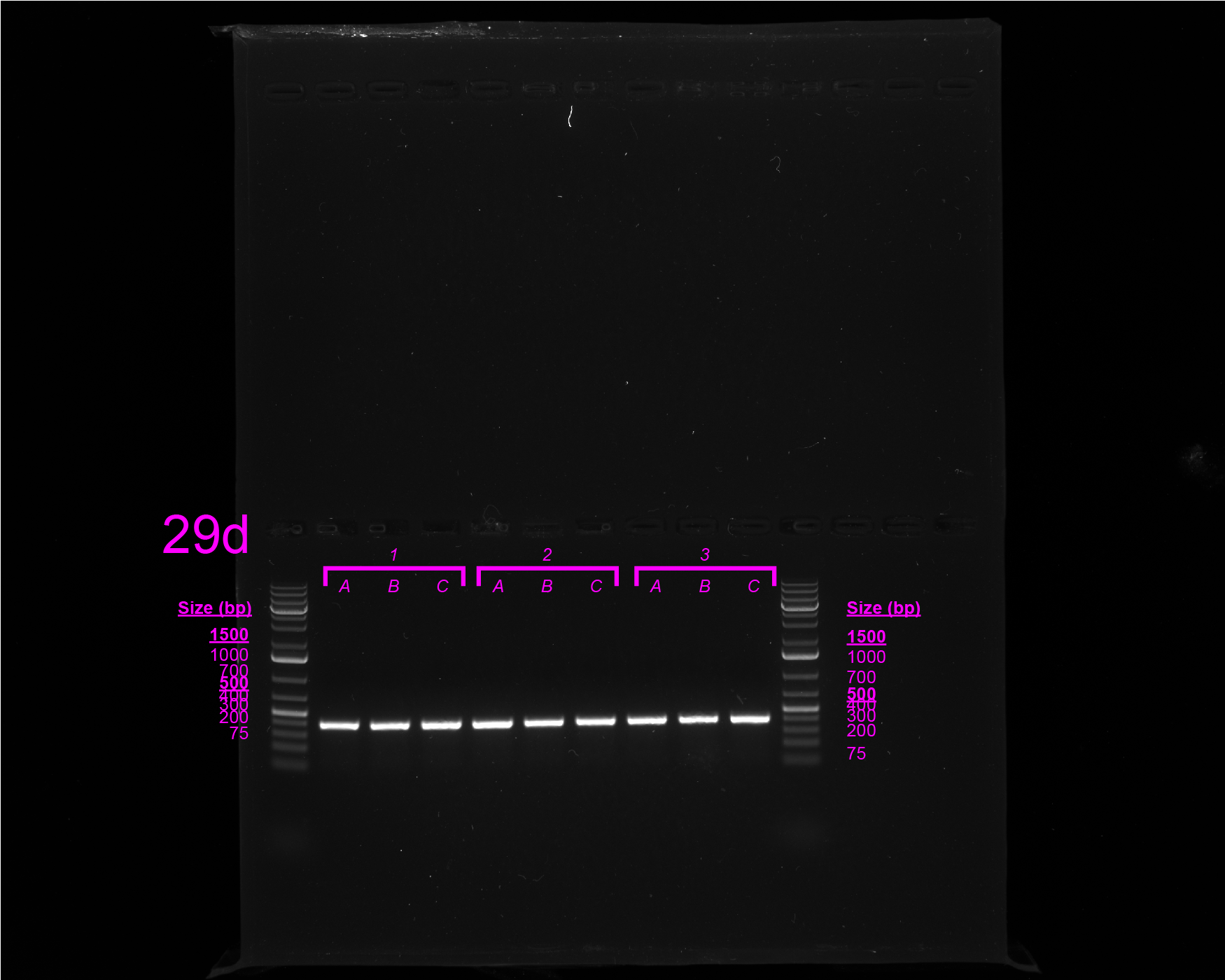

Supplement: Supplementary file 4 — Source Data for Supplementary figures. Supplementary Figs. 1, 25, 26, 27, 28, 29, 36, 37 contain Excel-incompatible data formats. [file 41564_2022_1258_MOESM4_ESM.zip › Source Data for Supplementary Figures/SuppFig29/SuppFig29d.png]

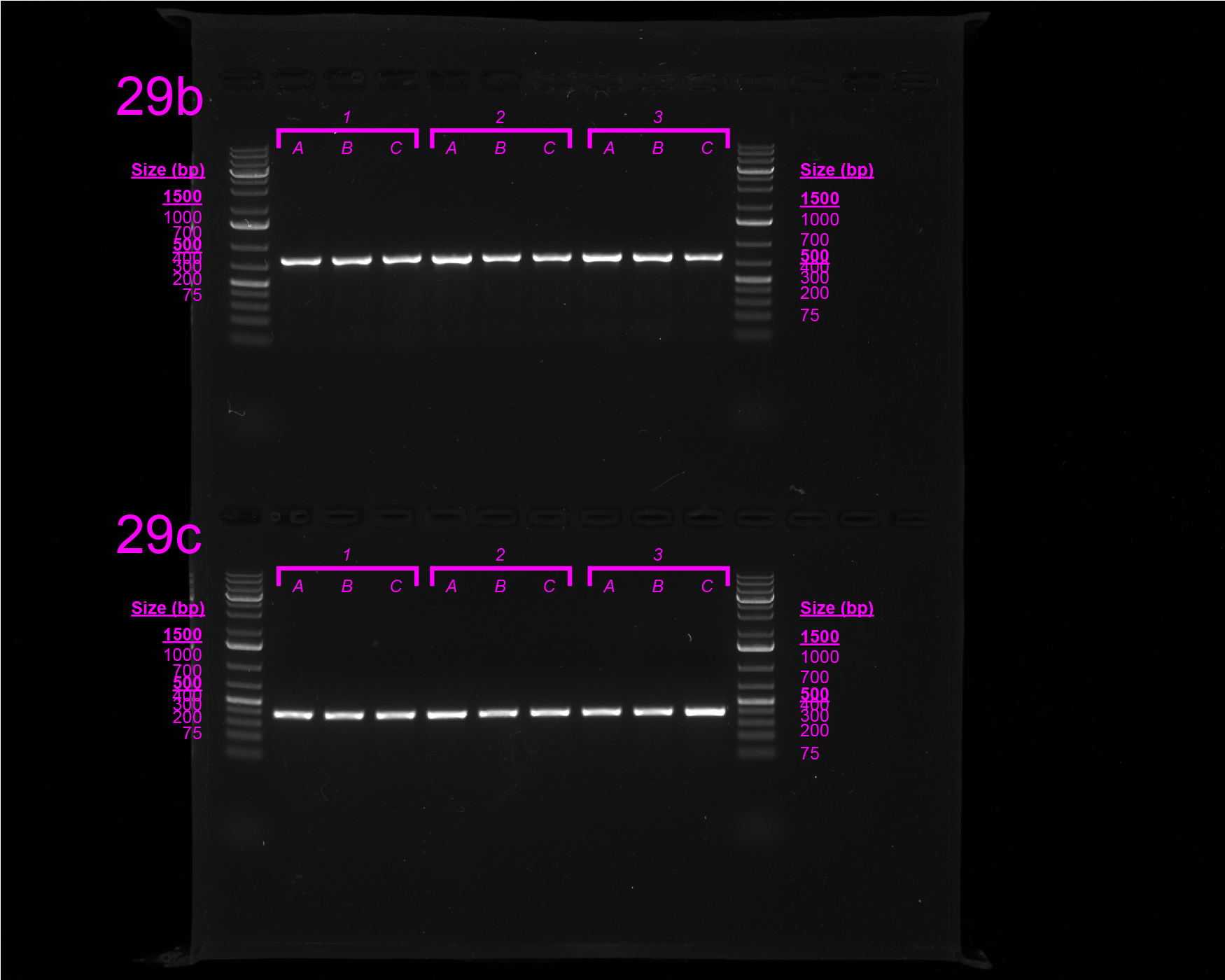

Supplement: Supplementary file 4 — Source Data for Supplementary figures. Supplementary Figs. 1, 25, 26, 27, 28, 29, 36, 37 contain Excel-incompatible data formats. [file 41564_2022_1258_MOESM4_ESM.zip › Source Data for Supplementary Figures/SuppFig29/SuppFig29bc.png]

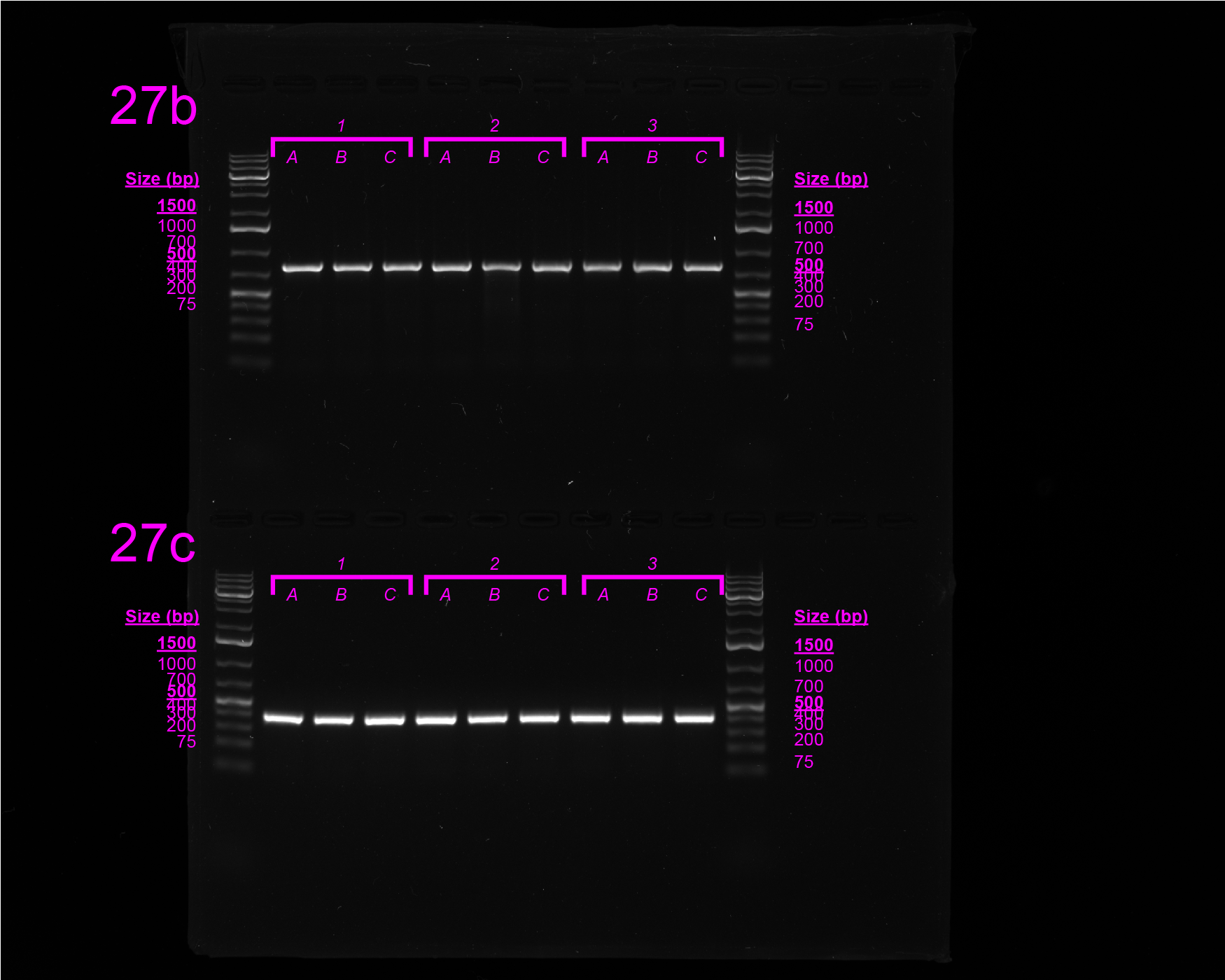

Supplement: Supplementary file 4 — Source Data for Supplementary figures. Supplementary Figs. 1, 25, 26, 27, 28, 29, 36, 37 contain Excel-incompatible data formats. [file 41564_2022_1258_MOESM4_ESM.zip › Source Data for Supplementary Figures/SuppFig27/SuppFig27bc.png]

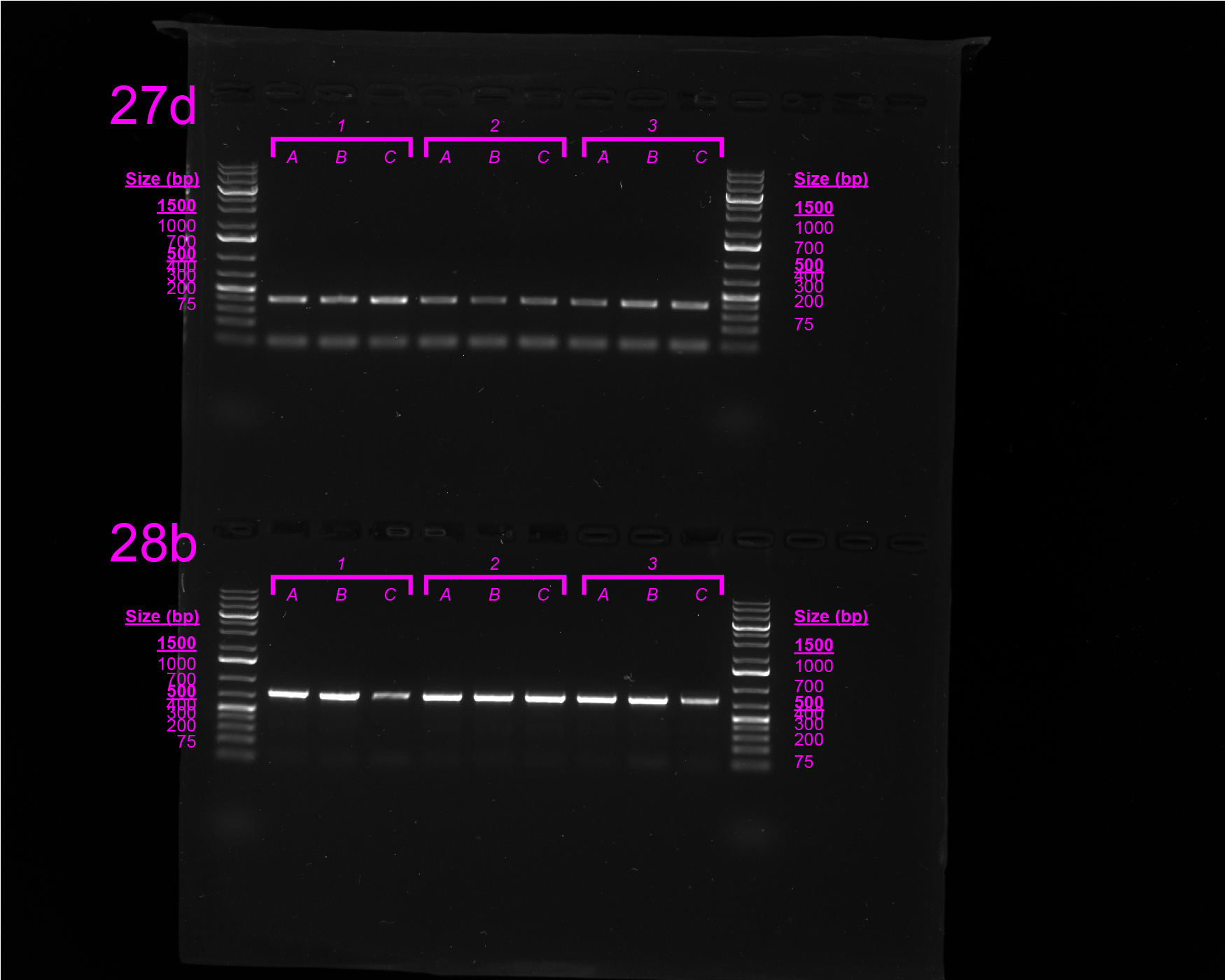

Supplement: Supplementary file 4 — Source Data for Supplementary figures. Supplementary Figs. 1, 25, 26, 27, 28, 29, 36, 37 contain Excel-incompatible data formats. [file 41564_2022_1258_MOESM4_ESM.zip › Source Data for Supplementary Figures/SuppFig27/SuppFig27d_SuppFig28b.png]

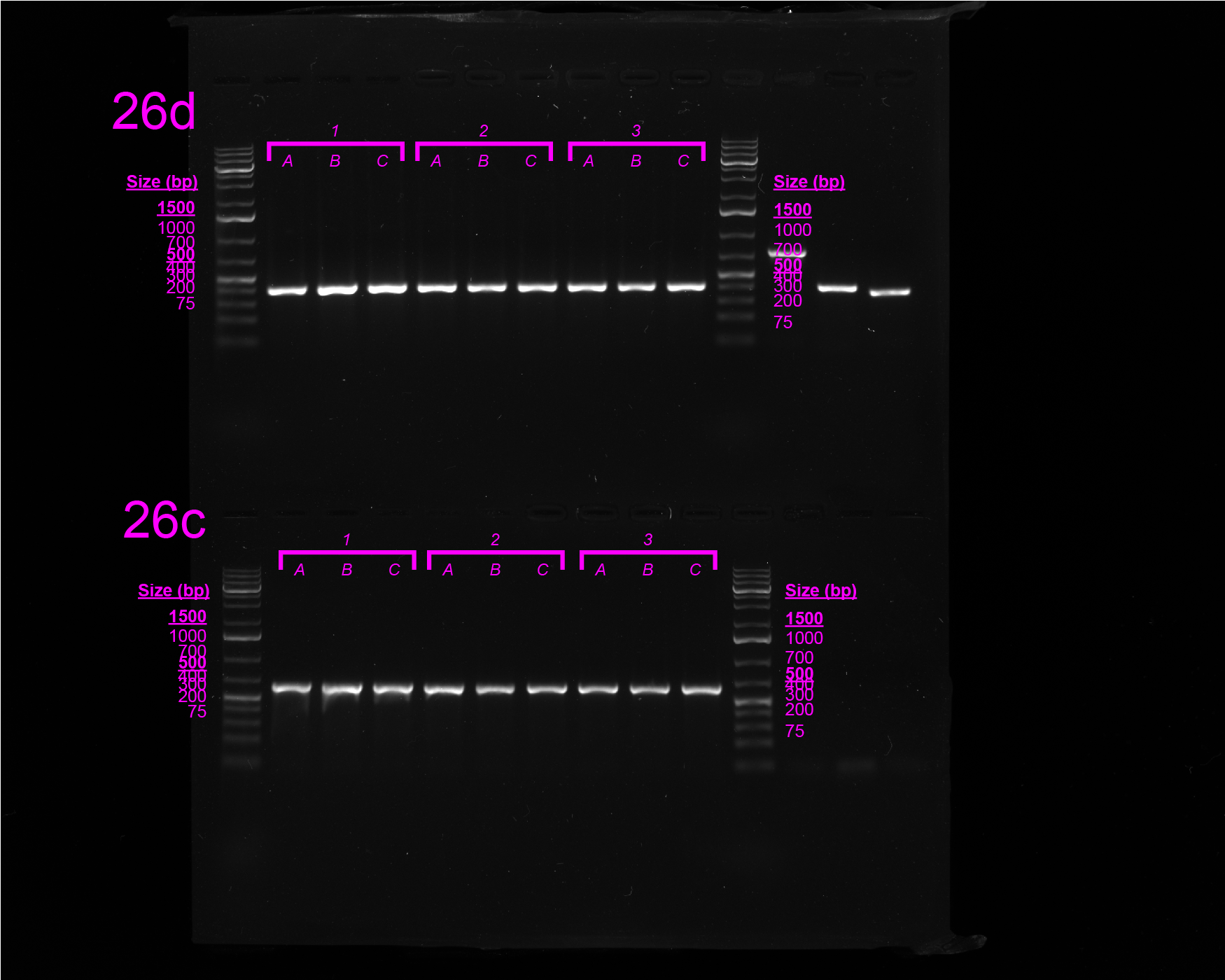

Supplement: Supplementary file 4 — Source Data for Supplementary figures. Supplementary Figs. 1, 25, 26, 27, 28, 29, 36, 37 contain Excel-incompatible data formats. [file 41564_2022_1258_MOESM4_ESM.zip › Source Data for Supplementary Figures/SuppFig26/SuppFig26cd.png]

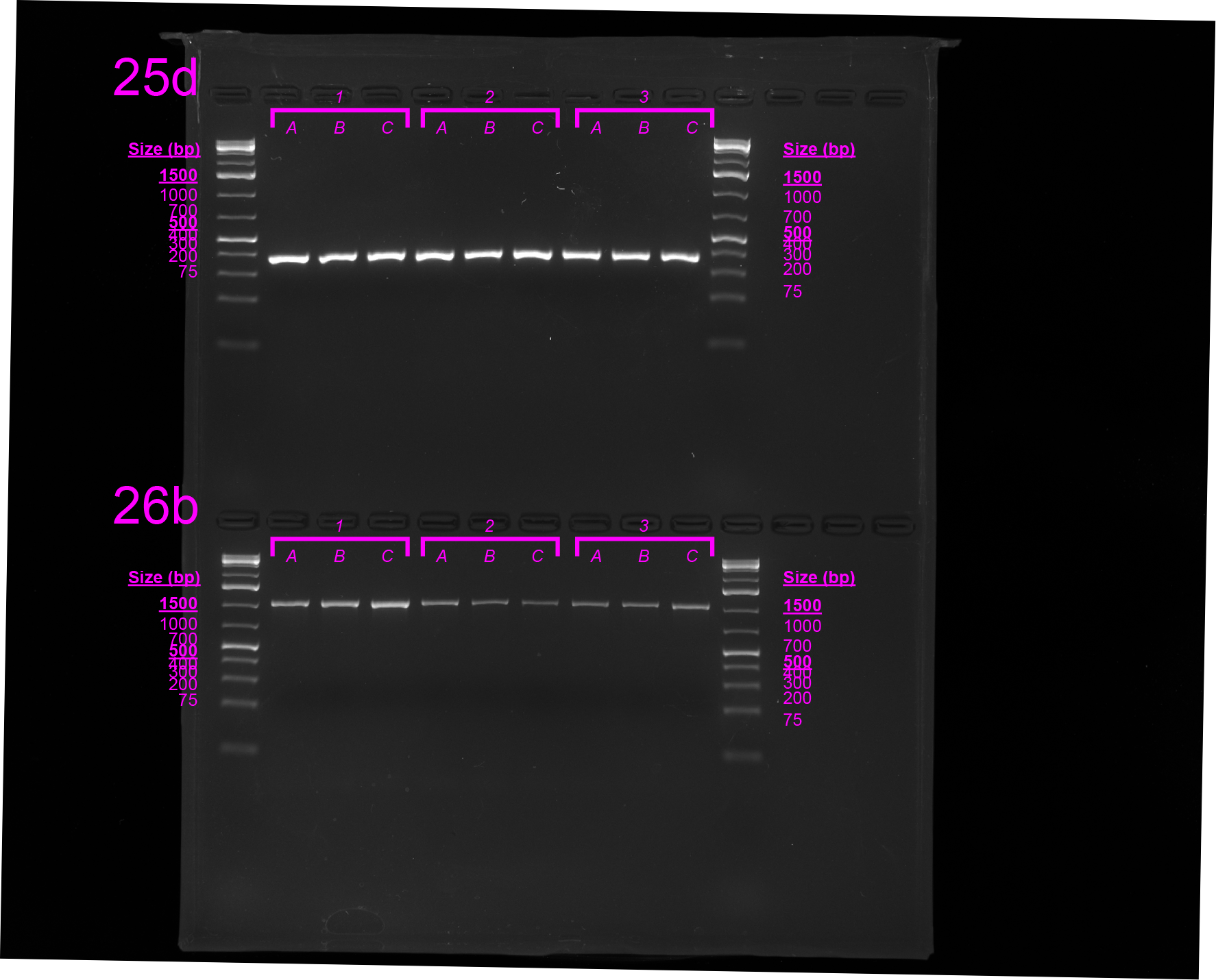

Supplement: Supplementary file 4 — Source Data for Supplementary figures. Supplementary Figs. 1, 25, 26, 27, 28, 29, 36, 37 contain Excel-incompatible data formats. [file 41564_2022_1258_MOESM4_ESM.zip › Source Data for Supplementary Figures/SuppFig26/SuppFig25d_SuppFig26b.png]

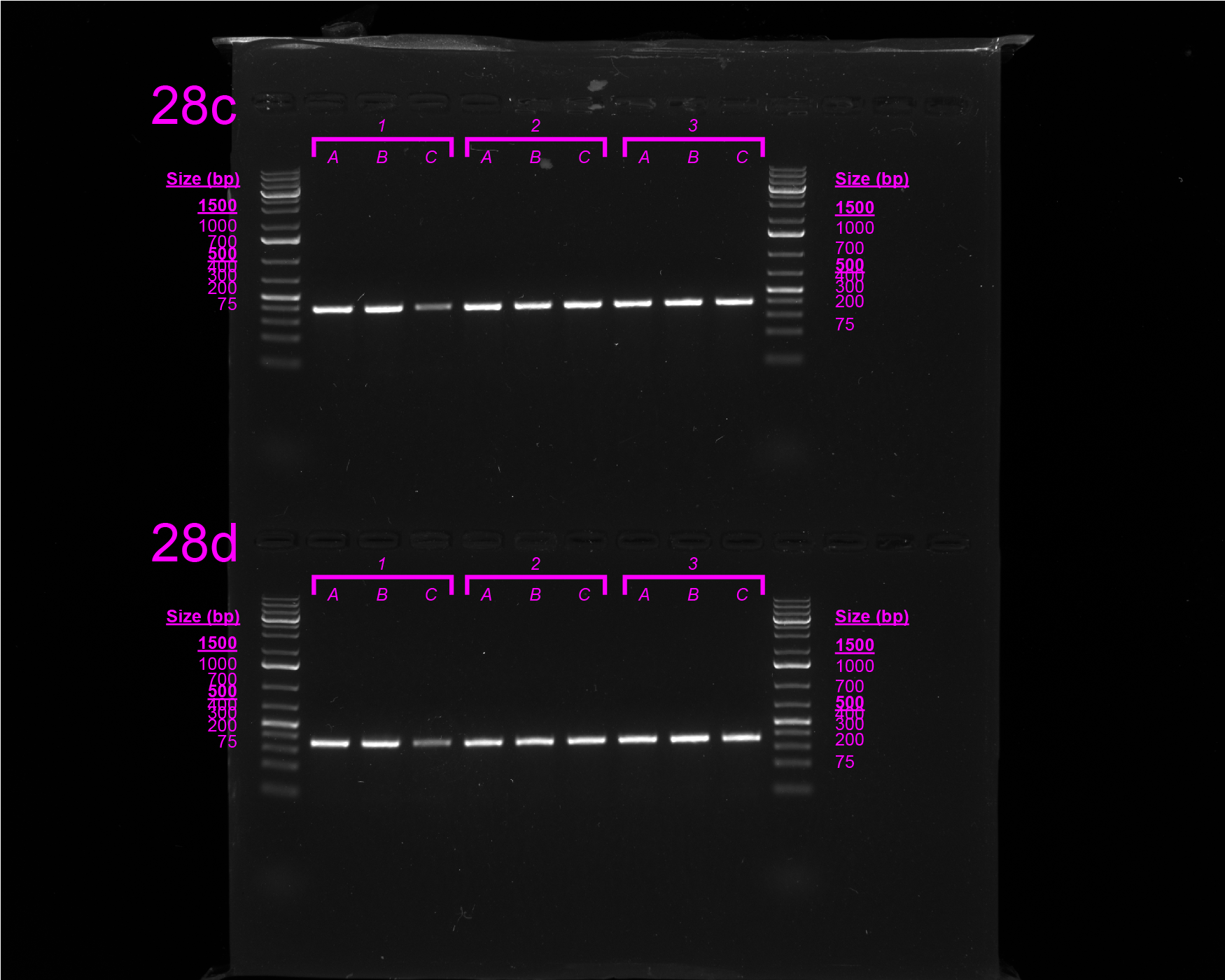

Supplement: Supplementary file 4 — Source Data for Supplementary figures. Supplementary Figs. 1, 25, 26, 27, 28, 29, 36, 37 contain Excel-incompatible data formats. [file 41564_2022_1258_MOESM4_ESM.zip › Source Data for Supplementary Figures/SuppFig28/SuppFig28cd.png]

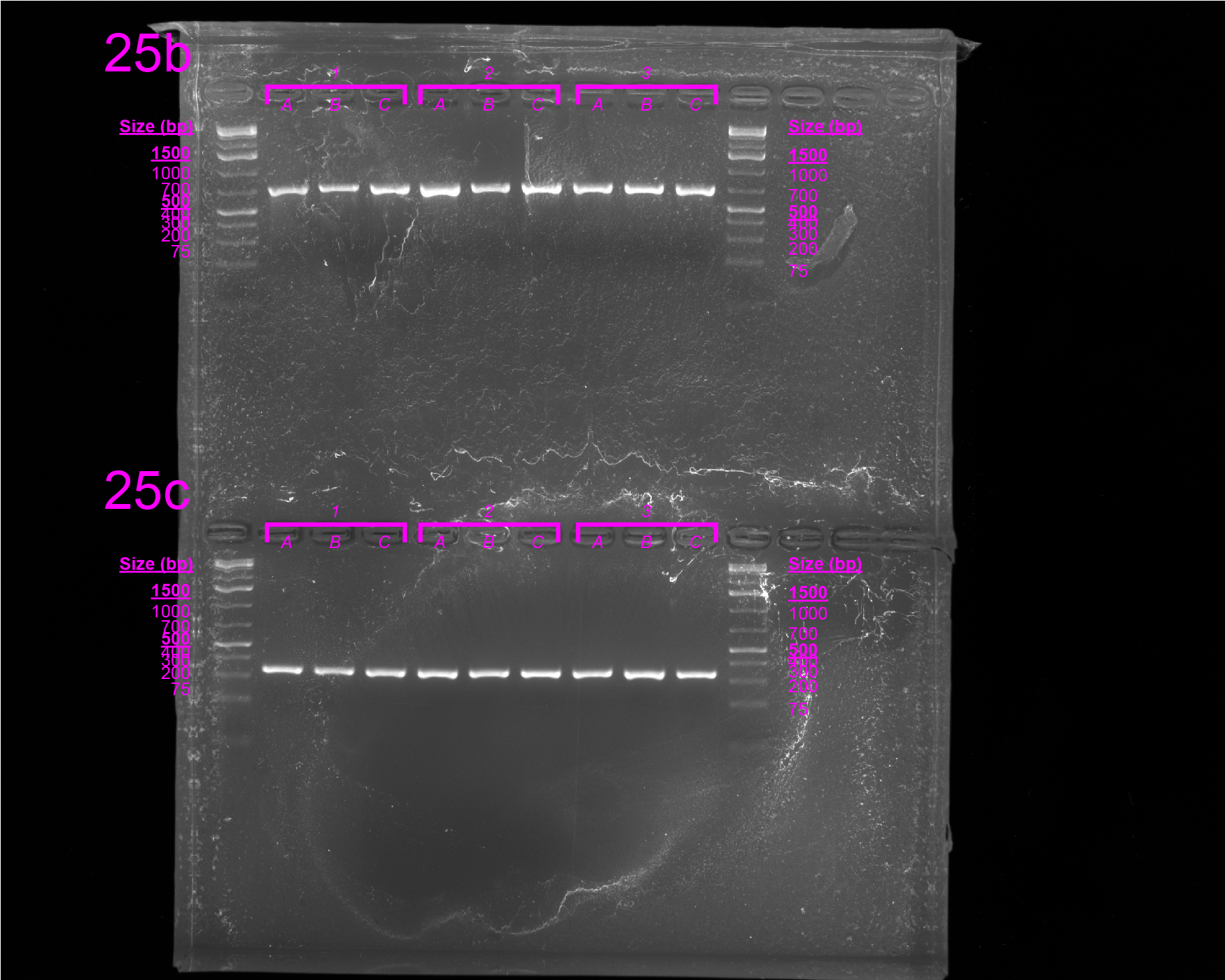

Supplement: Supplementary file 4 — Source Data for Supplementary figures. Supplementary Figs. 1, 25, 26, 27, 28, 29, 36, 37 contain Excel-incompatible data formats. [file 41564_2022_1258_MOESM4_ESM.zip › Source Data for Supplementary Figures/SuppFig25/SuppFig25bc.png]
